# Structure–Property Relationships of Near-Infrared Cyanine Dyes: Chalcogen-Driven Singlet Oxygen Generation with High Fluorescence Efficiency

**DOI:** 10.1021/acsomega.5c10499

**Published:** 2026-01-12

**Authors:** Shufan Yang, Ewan Forsyth, Wuyang Lin, Kerry Setchfield, Rachel Crespo-Otero, Devis Di Tommaso, Annamaria Lilienkampf, Amanda Wright, Mark Bradley

**Affiliations:** † School of Chemistry, 3124 University of Edinburgh, David Brewster Road, EH9 3FJ Edinburgh, U.K.; ‡ Precision Healthcare University Research Institute, Queen Mary University of London, Empire House, 67 New Road, E1 1HH London, U.K.; § Department of Chemistry, University of Oxford, Mansfield Rd, OX1 3TA Oxford, U.K.; ∥ School of Physical and Chemical Sciences, 4617 Queen Mary University of London, 327 Mile End Road, London E1 4NS, U.K.; ⊥ Electrical and Electronic Engineering, 121171 University of Nottingham, Life Sciences Building, Nottingham NG7 2RD, U.K.; # Department of Chemistry, 4919 University College London, 20 Gordon St, London WC1H 0AJ, U.K.; ∇ Digital Environment Research Institute, Queen Mary University of London, Empire House, 67 New Road, London E1 1HH, U.K.

## Abstract

We report the design, synthesis, and optical characterisations of eight novel near-infrared (NIR) cyanine dyes incorporating different chalcogens (O, S, and Se). These dyes exhibited excellent deep-NIR absorption (λ_max_ = 767–833 nm) and emission (λ_max_ = 784–859 nm) profiles. TDDFT calculations matched well the experimental trends and data. All compounds exhibited high extinction coefficients (178,000–267,000 cm^–1^ M^–1^) and good fluorescence quantum yields, resulting in high overall brightnesses. Remarkably, the selenium-containing dyes featuring terminal indole and benzoindole-type units exhibited impressive singlet oxygen quantum yields of around 13%, a standout performance in the deep-NIR region. These values are particularly promising and highlights the potential of these dyes for deep-NIR imaging and photodynamic applications.

## Graphical abstract

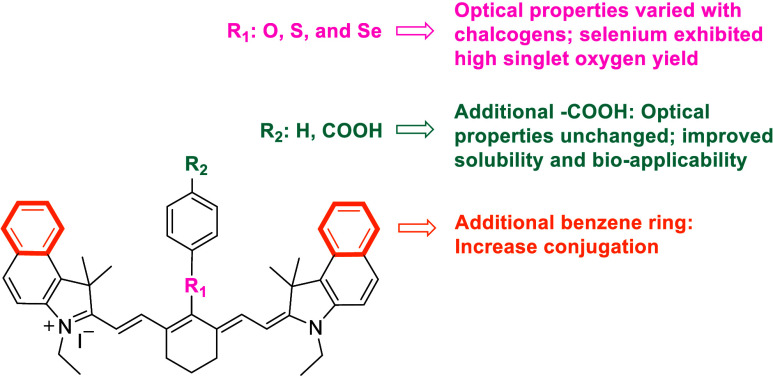

## Introduction

Near-infrared-II (NIR-II) and short-wave infrared (SW-IR) fluorescent imaging have emerged as powerful techniques with broad potential in biological and medical applications.^1–5^ This spectral range is advantageous for biological imaging due to reduced light scattering by tissue at these longer wavelengths, and minimal autofluorescence from biological tissues, allowing deeper tissue penetration.^6–8^ Indeed, ‘Deep-NIR cyanine dyes’’ (those with wavelengths greater than 750 nm) are prominent in this area, offering enhanced signal-to-noise ratios and improved depth penetration, making them attractive for a range of biomedical applications.^9^ There is also increasing demand for deep-NIR dyes that can also generate singlet oxygen - key for photodynamic therapy (PDT).^10–14^ PDT is a minimally invasive therapeutic approach that uses light-activated photosensitizers to produce cytotoxic reactive oxygen species, offering spatial and temporal control of treatment, killing when and where illumination occurs.^15^ The combination of imaging and therapeutic capabilities offers significant appliction and future promise for the treatment of cancer and other localized diseases.^16^

The broad biocompatibility, tunable photophysical properties and ease of chemical modification of the cyanine dyes has driven their utility. These dyes can be tailored, such as by the addition of solubilizing groups via sulfonylation, or the attachment of ammonium groups or PEG’s, or the provision of chemical handles to allow the attachment of ligands enabling the targeted imaging of specific tissues/cells/receptors.^17–19^ Of specific note is Indocyanine Green (ICG), a FDA-approved, near-infrared (NIR) fluorescent dye, that is widley used for medical diagnostics, such as measurement of cardiac output and liver function assessment.^20,21^ It absorbs (784 nm) and emits in the deep NIR (807 nm) allowing for deep tissue imaging, making it valuable in cancer diagnostics and surgery guidance.^22,23^

Many NIR cyanine dyes that have been reported.^24^ For example NIR-780, which is hydrophobic, shows preferential accumulation in tumors, which is useful for both imaging and so-called photothermal therapy (conversion of NIR light into heat to destroy cancer cells).^25,26^ NIR-783 is a commercial NIR heptamethine cyanine dye carrying a chemically reactive chloro-group on a cyclohexenyl ring (similar in function to the cell tracker dyes) and has been reported to be selective for imaging various cancer types, including breast and brain, perhaps via covalent albumin conjugation.^27,28^ The ability of NIR-783 to preferentially accumulate in tumor cells has been reported to enable both precise imaging and photothermal therapy.^19^ Structurally related is the more hydrophobic NIR-786, that also complexes to proteins such as albumin. It has been found to show tumor accumulation, making it effective in photothermal cancer treatment.^24^ Also in this family are CA-800Cl (here named NIR-800 to be consistent) and NIR-808 (also referred to as MHI-148),^24^ that exhibit similar optical properties, both with the incorporation of two carboxylic acid moieties. The later (NIR-808) has been applied in optical imaging, benefiting from its inherent tumor-targeting ability (via organic anion transporters) without requiring conjugation to targeting peptides.^29^

Recent studies have reported the synthesis of dual-modal imaging agents that combine NIR-II cyanine dyes with magnetic resonance imaging (MRI) contrast agents, offering a more comprehensive tool for tumor detection and surgical navigation.^30,31^ Polymer-conjugated cyanine dyes have also been reported, with cyanine dyes attached to biodegradable polymers to improve their circulation time in the bloodstream, making them more effective for long-term imaging and targeted delivery applications.^32,33^

Therefore, given their significant potential, new NIR cyanine dyes occupy an important position and yet still require development to promote chemical/photostability and the ability to offer therapeutic efficacy in the form of photodynamic therapy.^34^

A significant area of research within the realm of dyes/fluorophores involves the design and synthesis of dyes incorporating group 14 elements (beyond carbon) (crystallogens), group 15 elements (pnictogens), and group 16 elements (chalcogens). For example, Koide et al., explored how the integration of Group 14 elements (silicon, germanium, or tin) into rhodamine dyes changed their optical properties.^35^ By replacing oxygen atoms in traditional rhodamines with these elements, the dyes exhibited a red-shift in fluorescence emission, moving them into the near-infrared (NIR) range. As for group 15 elements (pnictogens), nitrogen is frequently incorporated into NIR dyes (in place of carbon), such as aza-BODIPY (Figure 1) and aminocyanines.^36,37^ Aza-BODIPY dye (Figure 1) exhibits strong absorption in the NIR region (λ_abs_ = 650 nm) following the introduction of additional nitrogen atoms into the linking backbone.^36,37^ These modifications shifting the absorption spectra toward longer wavelengths, enhances their flexibility for deep tissue imaging.^37^ Other group 15 elements are much less commonly incorporated e.g phosphorus or arsenic due to chemistry challenges and toxicity concerns, although, Adams et al., introduced a method for the fluorescent labeling of proteins with a Cys-Cys-Xaa-Xaa-Cys-Cys motif, binding so-called FlAsH (fluorescein with two As­(III) substituents).^38^

**Figure 1:**
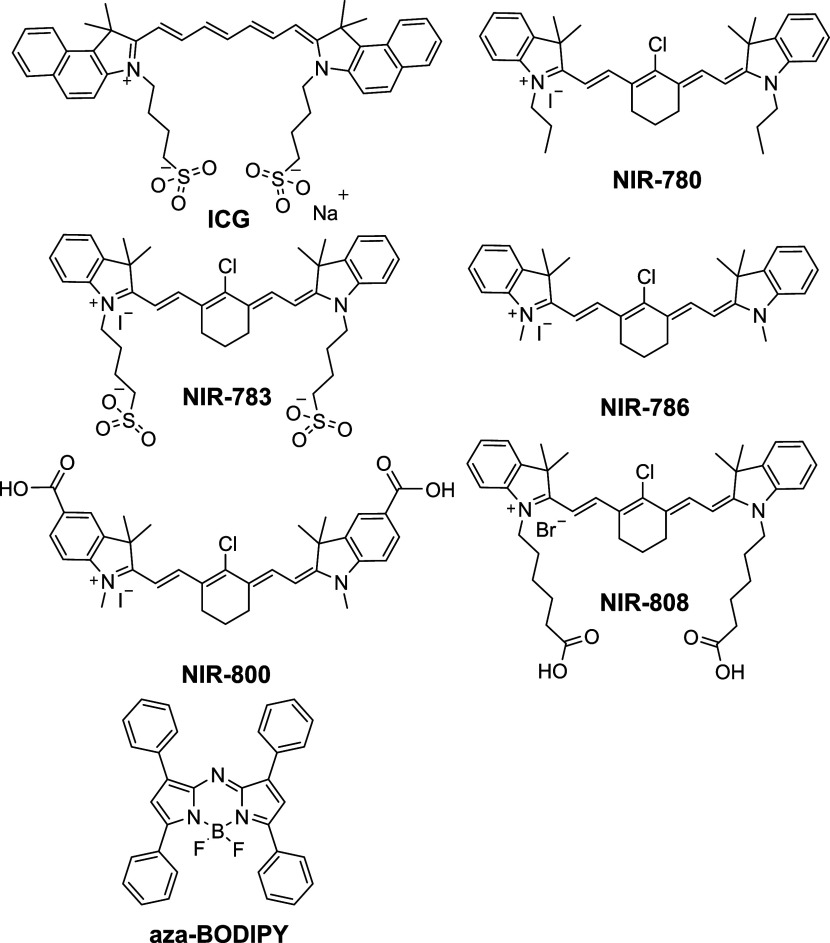
Chemical structures of ICG, NIR-780, NIR-783, NIR-786, NIR-808, NIR-800 and tetraphenyl substituted aza-BODIPY (with a formal zwitterionic structure).

In terms of group 16 (chalcogens), oxygen, sulfur, and selenium, can significantly influence the electronic structure and, consequently, the optical properties of the dyes.^39^ For example, replacing oxygen with sulfur or selenium can lead to red-shifted absorption and emission wavelengths, altered quantum yields, and changes in photostabilities.^40–42^ Liu et al. demonstrated this by modifying heptamethine cyanine dyes with sulphur or selenium, modifying the two indole (indol-1-ium) rings with thiazoles or selenazoles. The *bis*-seleno variant showed a strong redshift (∼840 nm) and a highly efficient singlet oxygen generation.^12^ Understanding the structure–property relationships of these chalcogen-containing dyes and their biological sensitivity and compatibility is important for biological application.

Here we explored the design and synthesis of novel near-infrared cyanine dyes with various chalcogens (O, S, and Se), examining their optical properties and structure–property relationships as well as their ability to generate reactive oxygen species. Increased conjugation at the terminal group altered the optical properties, causing a red shift in the spectrum. We also successfully introduced a −COOH group to some dyes, which preserved the optical properties while enhancing solubility and potential biological applicability. Density functional theory (DFT) and Time-dependent density functional theory (TDDFT) calculations were employed to investigate the optical properties of the chalcogen-containing dyes, showing good agreement with experimental results and supporting the observed trends. This manuscript highlights the potential of these dyes for photodynamic therapy-based applications, with significant levels of singlet oxygen generation by the NIR seleno-variants., which also proved to be remarkably stable to singlet oxygen generation, despite the known propensity of selenium to undergo facile oxidation.

## Result and Discussion

The dyes **SY 1**–**SY 8** were synthesized in three steps via N-alkylation of the “indole” or “benzoindole” terminating groups, *bis*-Knoevenagel condensation with the bifunctional core 2-chloro-1-methyl-3-methylenecyclohex-1-ene **11** and nucleophilic displacement of the “reactive” halogen (see Figure 3). They were readily prepared on-scale and purified. The benzoindole derivatives were expected to allow red-shifted absorptions/emissions compared to the indole variants. The nucleophiles were all chalcogen-based (phenol, thiophenol or selenophenol (benzeneselenol)) and gave the cyanine dyes **SY 1**–**SY 6** as green solids, while **SY 7** and **SY 8** were generated using 4-mercaptobenzoic acid, with the carboxylic acid group, enhancing their water-solubility. Due to their varying electronegativities and sizes (O: 3.44, 63 pm S: 2.58, 103 pm Se: 2.54, 116 pm), the chalcogen would be expected to alter the electronic distribution/conjugation and affect the optical properties of the dyes (Figures 2 and 3).

**Figure 2:**
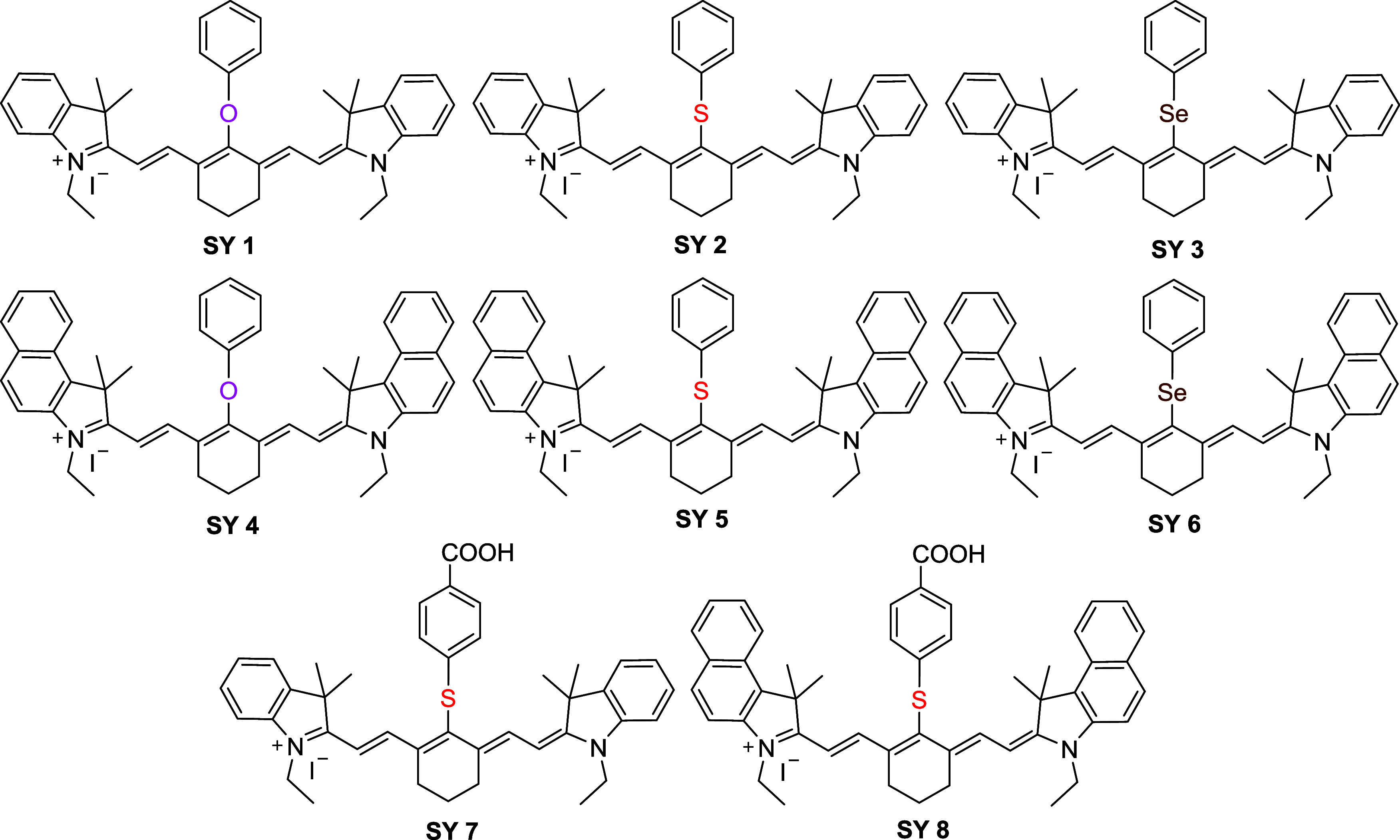
Chemical structures of the synthesized cyanine dyes **SY 1**–**SY 8**.

**Figure 3:**
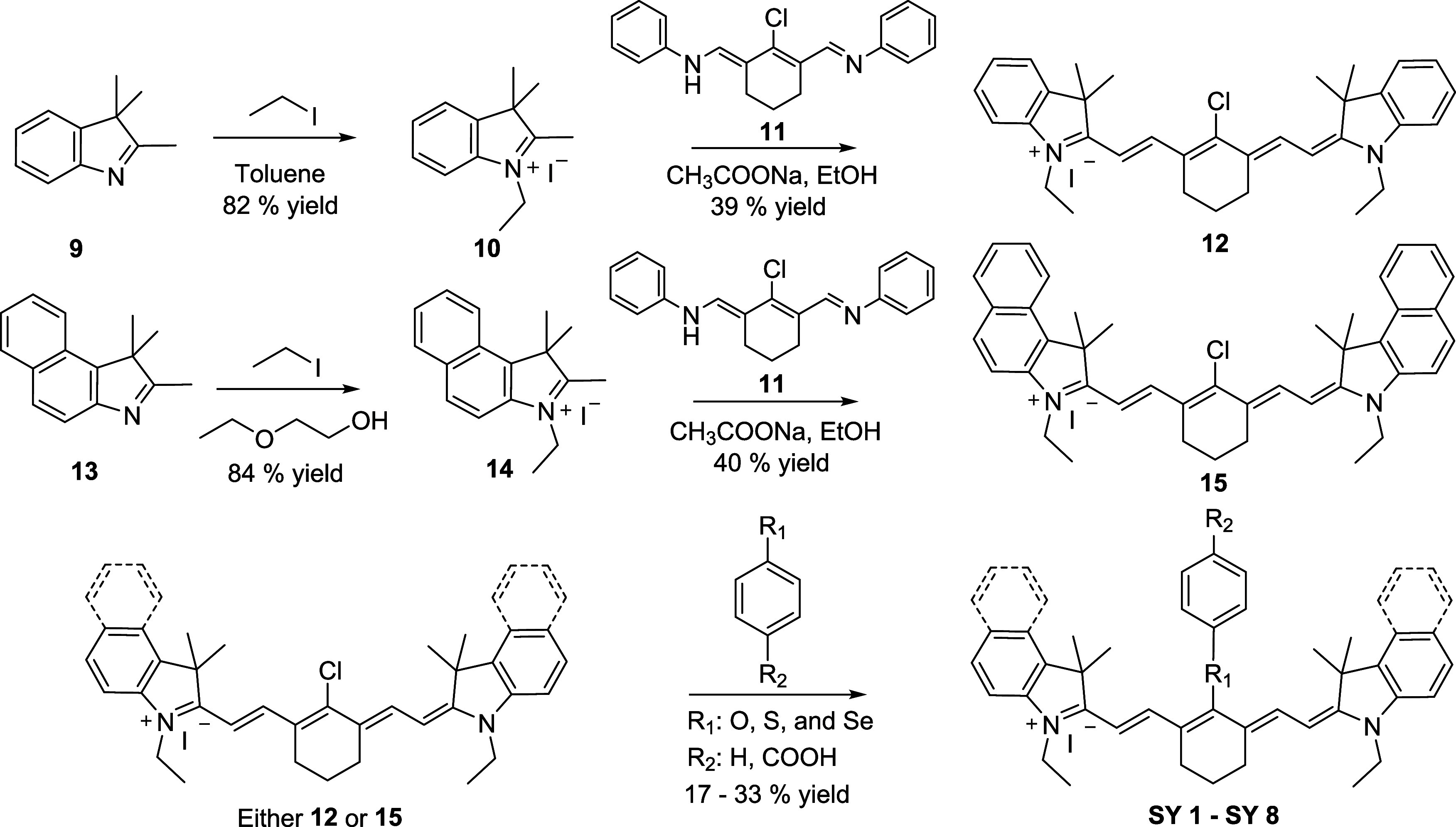
Synthetic route to the ‘deep-NIR’’ fluorophores and singlet oxygen generators **SY 1–SY 8**.

The purified dyes were examined optically with the optical properties varying with the indole/benzoindole head groups and the chalcogen (see Figure 4). With the same chalcogen the absorption spectrum red-shifted when using the different terminal indoles or benzoindoles (as expected). Thus, comparing **SY 1** to **SY 4**, the λ_max,abs_ increased from 767 to 805 nm; **SY 2** and **SY 5** increased from 793 to 833 nm; and there was an increase from 787 to 826 nm for **SY 3** and **SY 6**.

**Figure 4:**
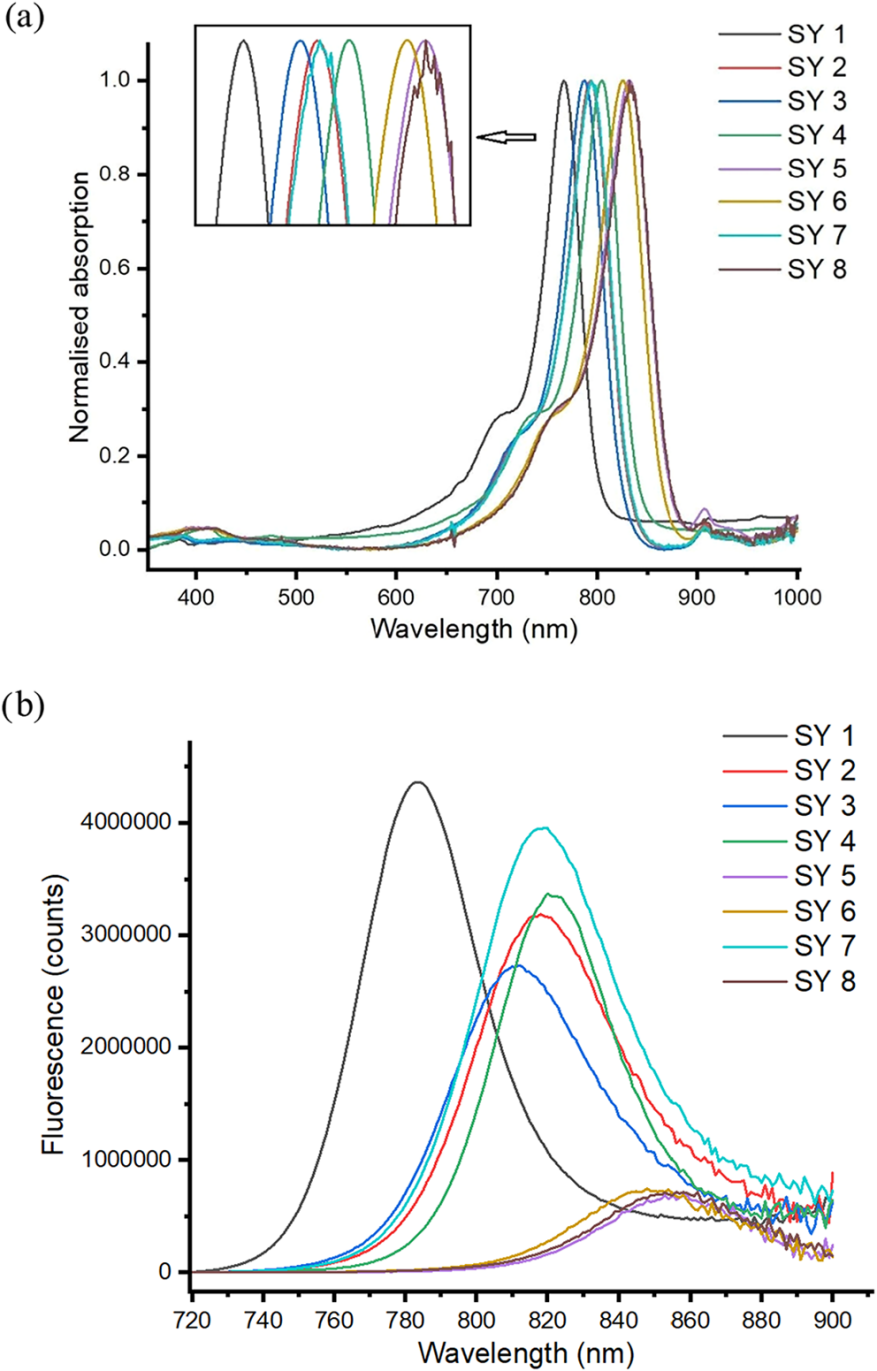
Optical properties of the dyes **SY 1–8**. (a) Normalized absorption spectra of the eight NIR dyes (**SY 1**–**SY 8**); (b) Fluorescence spectrum of the dyes **SY 1–8**. All the dyes were 2 μM in ethanol with excitation at 710 nm.

When the chalcogen was altered there were subtle changes in the absorption spectrum. In the indole series, from **SY 1** (O) to **SY 2** (S), there was a 26 nm red-shift observed, potentially due to improved conjugation and the reduced electron-withdrawing nature of the central ring. However, the change from **SY 2** (S) to **SY 3** (Se), was observed to result in a very slight blue-shift (793 to 787 nm). Selenium, being a little larger than sulfur and slightly less electronegative, potentially gives rise to this by slightly reducing conjugation across the system or forcing it out of planarity. Similarly, within the benzoindole series, there was a red-shift from 805 to 833 nm when comparing **SY 4** (O) and **SY 5** (S), and a slight blue shift between **SY 5 (S)** and **SY 6** (Se) (833 nm decreasing to 826 nm).

Fluorescence emission studies showed that **SY 1** (O) had a λ_max,em_ of 784 nm and **SY 4** (O) (the benzoindole variant) had a λ_max,em_ of 823 nm, both showing a 17 nm Stokes shift. **SY 3** (Se) had a λ_max,em_ of 812 nm with a Stokes shift of 25 nm, while **SY 6** (Se) with its increased conjugation has a λ_max,em_ of 854 nm (and a 28 nm Stokes shift). The dyes with the carboxylic acid groups (**SY 7** and **SY 8**) showed identical optical properties as the unmodified dyes (**SY 2** and **SY 5**).

The quantum yields (Φ_F_) of **SY 1**–**SY 8** ranged from 6 to 18%, with the benzoindole variants exhibiting a slightly lower Φ_F_ than the indole variants. The quantum yield values decreased from O > S > Se among both the indole series (**SY 1**–**SY 3**) and the benzoindole series (**SY 4**–**SY 6**) with values of 18, 13, and 11% and 14, 7, and 6% respectively. **SY 7** and **SY 8** had similar quantum yield to **SY 2** and **SY 5**.

Table 1 gives the summary of absorption, fluorescence and Stokes shift data, as well as their extinction coefficients, fluorescence quantum yields, brightness and singlet oxygen quantum yields for the 8 dyes. It is worthwhile to note that all dyes exhibit high molar extinction coefficients ranging from 178,000 to 267,000 cm^–1^ M^–1^, which along with their fluorescence quantum yields (between 6–18%), resulted in high brightness values between 10,700 and 47,800. There was no quenching observed, even at 5 μM, suggesting the absence of any aggregation effects.

**Table 1:**
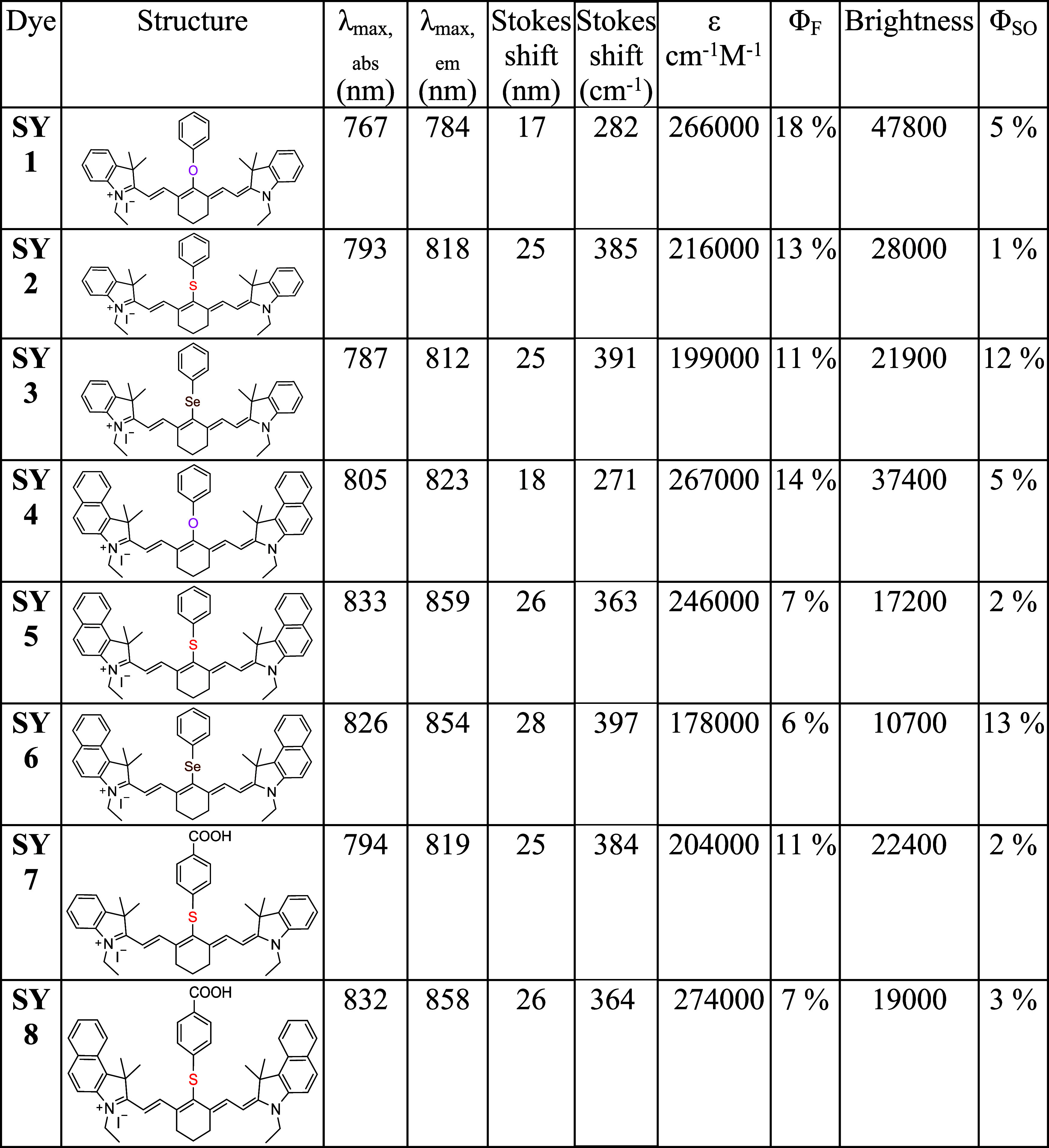
Extinction coefficients (ε) of each dye were measured from three concentrations (1.25 μM, 2.5 μM and 5 μM). Fluorescence quantum yield measurements of **SY 1** – **SY 8** were carried out in ethanol (2 μM); Indocyanine Green in ethanol (ICG, Φ = 13.2 %) was used as the reference for fluorescence quantum yield measurements.^49^ The singlet oxygen quantum yields were measured using Methylene blue in MeOH (ΦΔ = 52 %) as a reference, with each dye evaluated at 5 μM in MeOH.^50^

The singlet oxygen quantum yield efficiencies (Φ_SO_) of **SY 1**–**SY 8** were measured by monitoring the changes in absorbance of 1,3-diphenylisobenzofuran at 410 nm when exposed to the NIR dyes under irradiation (see SI Figure 2). Table 1 gives the singlet oxygen quantum yield efficiencies which ranged from 1 to 13%, much higher than ICG (with a Φ_SO_ of approximately 0.9%),^43^ although having a similar cyanine conjugation system. There was no significant change observed in Φ_SO_ between dyes in the indole or benzoindole series with the same chalcogen. Incorporation of Se in dyes **SY 3** and **SY 6** increased singlet oxygen quantum efficiencies (12 and 13% respectively see Figure 5) when compared with their oxygen **SY 1** and **SY 4** (both 5%),^44^ and sulfur counterparts **SY 2** (1%) and **SY 5** (2%).^12,45^ **SY 7** and **SY 8** exhibited similar Φ_SO_ as **SY 2** and **SY 5** (2 and 3%).

**Figure 5:**
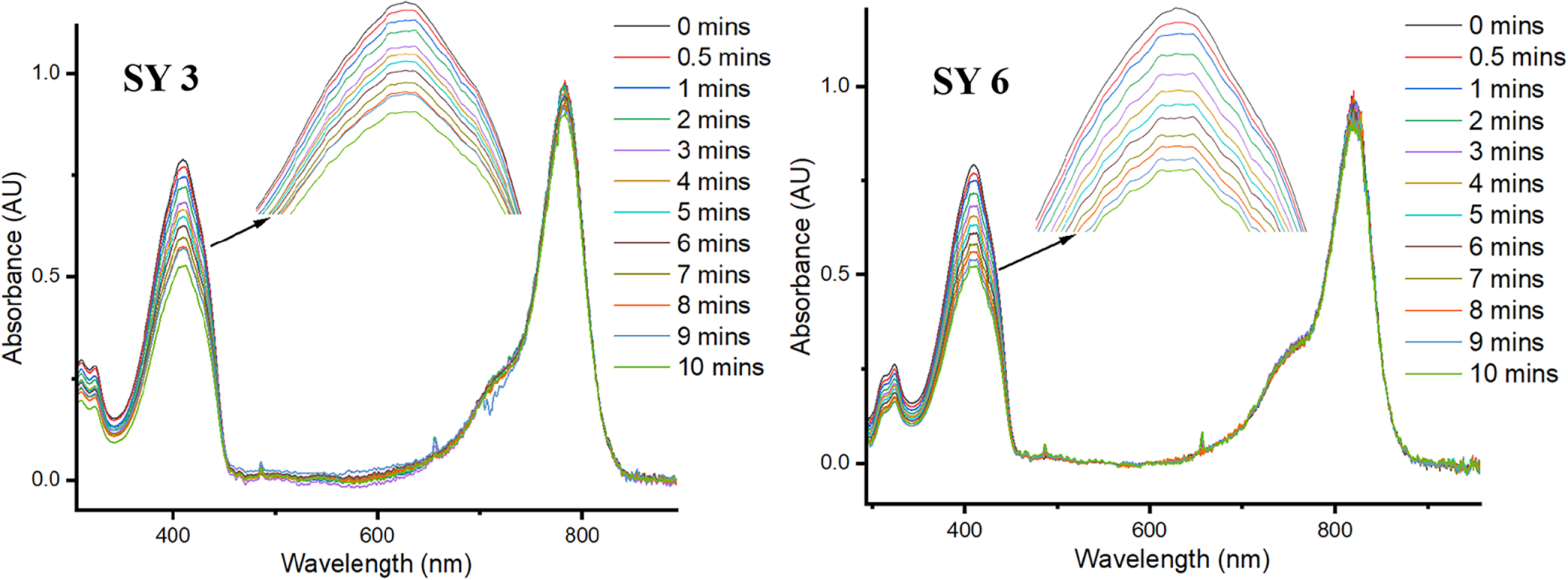
Singlet oxygen generation by **SY 3** and **SY 6**. Change of absorbance with time for DPBF (50 μM) in MeOH treated with **SY 3** and **SY 6** (5 μM) upon irradiation (lamp power 0.02 W/cm^2^, long pass filter > 490 nm).

**SY 3** and **SY 6** which both contain the chalcogen selenium, benefit from longer excitation wavelengths (>810 nm) and showed the highest singlet oxygen generation efficiencies of all the dyes (approximately 13%).^46–48^ Analysis (HPLC) showed that the seleno-dyes remained unchanged during extended irradiation (as do their absorbances as shown in Figure 5), confounding fears of selenium oxidation by the photogenerated singlet oxygen. The calculated S_1_-T_1_ gap for all the dyes **SY 1**–**SY 6** were approximately 0.8 eV, indicating an energetically accessible intersystem crossing (ISC) pathway, and supporting the proposed singlet oxygen generation mechanism (Table S9).

## Theoretical Investigations

To explore the absorption and fluorescence emission processes/properties, density functional theory (DFT) and time-dependent density functional theory (TD-DFT) calculations were performed on the dyes **SY 1–SY 8** (for details see the SI Section). The optimized structures of **SY 1–SY 8**, along with the corresponding HOMO and LUMO orbital contours, are shown in Figure 6. In the indole series, the oxygen-containing dye **SY 1** had the largest gap (2.04 eV), while the S-containing dye **SY 2** (1.95 eV) showed a similar gap to the Se-containing dye **SY 3** (1.93 eV). The largest gap for **SY 1** may originate from the more electronegative character of O, which leads to higher electronic localization. The energy gaps decreased with the reduction in electronegativity from O to S, to Se. The benzoindole series exhibited a smaller HOMO–LUMO gap compared to the indole counterpart, with the different chalcogens exhibiting a similar trend to that observed in the previous series. The calculated gaps were 1.95 eV for **SY 4** (O), and 1.88 eV for SY **5** (S) and **SY 6** (Se). The additional ring in the benzoindoles series enhancing the delocalization of electrons. The carboxylic group did not cause any noticeable differences in the HOMO–LUMO gap. These excitation energies accurately reproduce the experimentally observed λ_max_ values across the indole and benzoindole series with the different chalcogens (Table 2).

**Figure 6:**
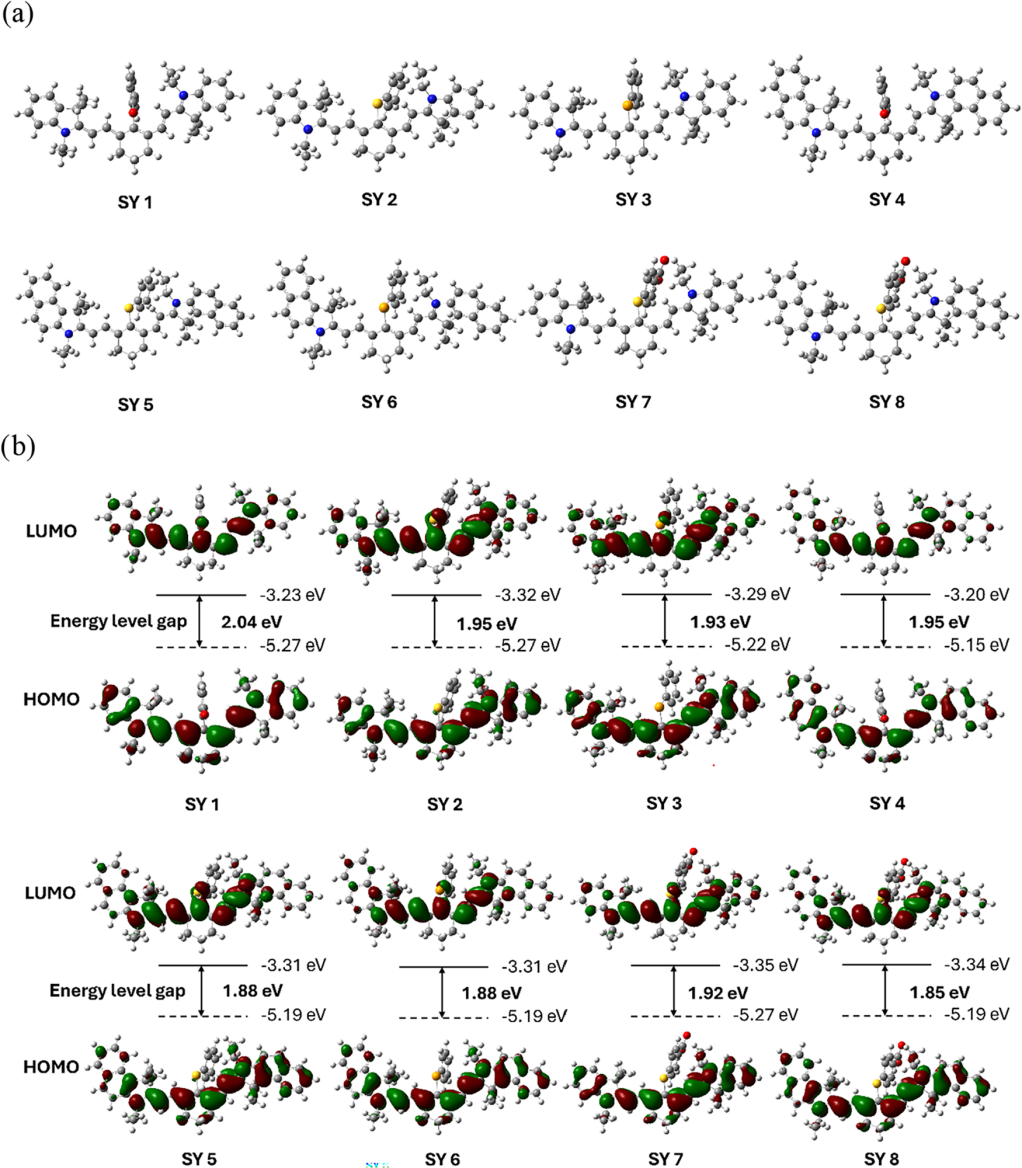
(a) Optimized geometric structures of **SY 1–SY 8**, respectively. Gray: carbon, red: oxygen, blue: nitrogen, white: hydrogen, yellow: sulfur and orange: selenium; (b) HOMO and LUMO orbital contours of **SY 1–SY 8**.

**Table 2:** Summary of the results of the energy gap calculations for the absorption and fluorescence emission processes, along with the corresponding spectral peaks for each dye. *E*_g_: HOMO-LUMO energy gap; *E*_abs,cal_: TD-DFT excitation energy from the S_0_ geometry to the first singlet excitation energies. *E*_abs,exp_: Experimental absorption. *E*_ems,cal_: TDDFT emission energy from S1 geometry; *E*_ems,exp_: Experimental emissions; *f*_cal_: Calculated oscillator strengths. Values of the energies in eV and of the oscillatory strengths in arbitrary units.

NIR dyes	HOMO (eV)	LUMO (eV)	*E*_g_ (eV)	*E*_abs,cal_ (eV)	*E*_abs,exp_ (eV)	*E*_ems,cal_ (eV)	*E*_ems,exp_ (eV)	*f*_cal_
**SY 1**	–5.27	–3.23	2.04	1.94	1.62	1.64	1.58	2.0293
**SY 2**	–5.27	–3.32	1.95	1.85	1.56	1.49	1.52	1.8088
**SY 3**	–5.22	–3.29	1.93	1.87	1.57	1.51	1.53	2.0008
**SY 4**	–5.15	–3.2	1.95	1.82	1.54	1.48	1.51	2.0758
**SY 5**	–5.19	–3.31	1.88	1.75	1.48	1.45	1.44	1.9303
**SY 6**	–5.19	–3.31	1.92	1.76	1.50	1.46	1.45	1.9268
**SY 7**	–5.27	–3.35	1.92	1.84	1.56	1.48	1.51	1.8735
**SY 8**	–5.19	–3.34	1.85	1.74	1.49	1.45	1.45	1.9818

The TD-DFT values for fluorescence energies and emission wavelengths further support the experimental results. Calculations conducted on the first excited state were used to analyze the fluorescence emission process, and determined the energy gaps of 1.64, 1.49, and 1.51 eV for the three indole dyes (**SY 1–SY 3**) and 1.48, 1.45, and 1.46 eV for the three benzoindole dyes (**SY 4–SY 6**). Similar to the absorption process, the additional carboxylic acid group has little impact on the fluorescence emission. Our computational results suggest that O-containing dyes displayed the largest energy gap in both absorption and fluorescence emission processes, followed by Se- and S-containing dyes, while the indole series displayed larger gaps than the benzoindoles. The attached carboxylic acid group exerted barely any effect on the properties of these dyes. Given the inverse relationship between the energy gaps and emission spectrum, these results agree well with our experimental results, specifically, the blue shift caused by the O and the red shift caused by S in the same series.

## Experimental Section

### The Synthesis of **SY 1**

The synthesis of **SY 1** followed the synthetic procedure of Wang.^51^ **12** (80 mg, 0.125 mmol) and phenol (117 mg, 1.25 mmol) were dissolved in DMF (5 mL), and triethylamine (0.1 mL) was added. The reaction mixture was stirred under an N_2_ atmosphere for 24 h at room temperature. After the reaction was complete (shown by TLC), the solvent was removed in vacuo and the residue purified by column chromatography on silica gel (eluting with dichloromethane: ethanol = 100:1 to 20:1), yielding the title compound **SY 1** as a green solid (23 mg, 26%). ^**1**^ **H NMR** (400 MHz, CDCl_3_) δ 7.87 (d, 2H, *J* = 14.2 Hz), 7.35–7.29 (m, 4H), 7.21 (d, 2H, *J* = 6.7 Hz), 7.14 (t, 2H, *J* = 7.4 Hz), 7.09 (d, 2H, *J* = 8.0 Hz), 7.02 (d, 2H, *J* = 7.9 Hz), 6.98 (apparent t, 1H, *J* = 7.4 Hz), 6.02 (d, 2H, *J* = 14.2 Hz), 4.12 (q, 4H, *J* = 7.2 Hz), 2.71 (apparent t, 4H, *J* = 6.0 Hz), 2.05–1.99 (m, 2H), 1.37 (t, 6H, *J* = 7.2 Hz), 1.28 (s, 12H). ^**13**^ **C NMR** (100 MHz, CDCl_3_) δ 171.4, 164.1, 159.7, 142.2, 141.7, 141.1, 130.3, 128.7, 125.0, 122.5, 122.4, 122.1, 114.5, 110.5, 99.7, 48.9, 39.8, 27.8, 24.6, 21.1, 12.4. **HRMS** (ES *m*/*z*): [M]^+^ calc. for C_40_H_45_N_2_O_1_: 569.35264, found: 569.3514.

### The Synthesis of **SY 2**

**12** (80 mg, 0.108 mmol) and thiophenol (36 mg, 0.324 mmol) were dissolved in degassed DMF (10 mL). The reaction was stirred until **12** had been consumed (TLC analysis). DMF was removed under *vacuum* at 50 °C, and the residue was washed with diethyl ether. The solid was purified by column chromatography on silica gel (eluting with dichloromethane: acetone = 100:1 to 4:1) to yield the green title compound **SY 2** (26 mg, 33%). ^**1**^ **H NMR** (400 MHz, CDCl_3_) δ 8.68 (d, 2H, *J* = 14.2 Hz), 7.35 (td, 2H, *J* = 1.2 Hz, *J* = 7.9 Hz), 7.29–7.26 (m, 2H), 7.25–7.23 (m, 2H), 7.20–7.17 (m, 4H), 7.13 (d, 2H, *J* = 8.0 Hz), 7.09–7.04 (m, 1H), 6.21 (d, 2H, *J* = 14.1 Hz), 4.20 (q, 4H, *J* = 7.2 Hz), 2.79 (apparent t, 4H, *J* = 6.1 Hz), 2.07–2.01 (m, 2H), 1.46 (s, 12H), 1.42 (t, 6H, *J* = 7.2 Hz); ^**13**^ **C NMR** (100 MHz, CDCl_3_) δ 171.9, 151.5, 146.3, 141.8, 141.3, 137.3, 134.3, 129.6, 128.8, 126.1, 125.8, 125.3, 122.3, 110.7, 101.4, 49.3, 40.1, 27.9, 26.9, 20.9, 12.6. **HRMS** (ES *m*/*z*): [M]^+^ calc. for C_40_H_45_N_2_S_1_: 585.32980, found: 585.3311.

### The Synthesis of **SY 3**

**12** (80 mg, 0.108 mmol) and 3 equiv of benzeneselenol (52 mg, 0.324 mmol) were stirred in anhydrous DMF (20 mL) under N_2_ (ballon). The solution was stirred at room temperature overnight. TLC inspection of the reaction showed **12** had been completely consumed. The DMF was removed under *vacuum* at 50 °C and the resulting crude product was purified by silica gel column chromatography (eluting with DCM/acetone = 30:1) to give the green target compound **SY 3** (22 mg, 27%). ^**1**^ **H NMR** (400 MHz, CDCl_3_) δ 8.73 (d, 2H, *J* = 14.1 Hz), 7.35 (td, 2H, *J* = 1.2 Hz, *J* = 7.86 Hz), 7.29–7.26 (m, 4H), 7.23–7.16 (m, 4H), 7.14–7.07 (m, 3H), 6.18 (d, 2H, *J* = 14.1 Hz), 4.19 (q, 4H, *J* = 7.2 Hz), 2.78 (apparent t, 4H, *J* = 6.1 Hz), 2.05–1.99 (m, 2H), 1.48 (s, 12H), 1.42 (t, 6H, *J* = 7.2 Hz); ^**13**^ **C NMR** (100 MHz, CDCl_3_) δ 171.9, 154.7, 149.4, 141.8, 141.3, 134.7, 132.4, 129.8, 128.8, 128.5, 126.4, 125.2, 122.3, 110.7, 101.2, 49.3, 40.0, 27.8, 27.3, 21.0, 12.5. **HRMS** (ES *m*/*z*): [M]^+^ calc. for C_40_H_45_N_2_Se_1_: 633.27425, found: 633.2732.

### The Synthesis of **SY 4**

The synthesis of **SY 4** followed the same procedure as **SY 1**, using **15** (90 mg, 0.121 mmol) and 3 equiv of phenol (35 mg, 0.365 mmol). The crude was purified by silica gel chromatography (eluting with dichloromethane/ethanol = 100:1 to 10:1) to give the title compound **SY 4** as a dark green solid (29 mg, 30% yield) ^**1**^ **H NMR** (400 MHz, CDCl_3_) δ 8.01–7.95 (m, 4H), 7.90–7.86 (m, 4H), 7.53 (t, 2H, *J* = 7.2 Hz), 7.42–7.38 (m, 6H), 7.11 (d, 2H, *J* = 8.3 Hz), 7.03 (apparent t, 1H, *J* = 7.3 Hz), 6.07 (d, 2H, *J* = 14.3 Hz), 4.26 (q, 4H, *J* = 7.1 Hz), 2.81–2.72 (m, 4H), 2.08–2.05 (m, 2H), 1.61 (s, 12H), 1.44 (t, 6H, *J* = 7.0 Hz); ^**13**^ **C NMR** (100 MHz, CDCl_3_) δ 172.8, 163.7, 159.8, 141.2, 139.2, 133.8, 131.8, 130.8, 130.4, 130.1, 128.1, 127.7, 124.9, 122.5, 122.2, 122.0, 114.6, 110.6, 99.3, 50.8, 40.1, 27.4, 24.7, 21.2, 12.7. **HRMS** (ES *m*/*z*): [M]^+^ calc. for C_48_H_49_N_2_O_1_: 669.38394, found: 669.3837.

### The Synthesis of **SY 5**

The synthesis of **SY 5** followed the same procedure as **SY 2**, using **15** (90 mg, 0.121 mmol) and 3 equiv of thiophenol (40 mg, 0.365 mmol). The crude was purified by column chromatography on silica gel, eluting with dichloromethane/acetone from 50:1 to 20:1 to yield the title compound **SY 5** (25 mg, 26%) as a dark green solid. ^**1**^ **H NMR** (400 MHz, CDCl_3_) δ 8.78 (d, 2H, *J* = 14.2 Hz), 8.02 (d, 2H, *J* = 8.51 Hz), 7.91–7.87 (m, 4H), 7.56–7.52 (m, 2H), 7.43–7.39 (m, 4H), 7.27–7.24 (m, 4H), 7.08–7.03 (m, 1H), 6.22 (d, 2H, *J* = 14.2 Hz), 4.31 (q, 4H, *J* = 7.2 Hz), 2.80 (apparent t, 4H, *J* = 6.1 Hz), 2.08–2.03 (m, 2H), 1.74 (s, 12H), 1.46 (t, 6H, *J* = 7.2 Hz); ^**13**^ **C NMR** (100 MHz, CDCl_3_) δ 173.2, 150.8, 145.3, 139.2, 137.3, 134.0, 133.9, 131.9, 130.8, 130.1, 129.5, 128.1, 127.7, 126.1, 125.7, 125.1, 122.1, 110.7, 100.8, 51.0, 40.3, 31.0, 27.4, 26.8, 12.9. **HRMS** (ES *m*/*z*): [M]^+^ calc. for C_48_H_49_N_2_S_1_: 685.36110, found: 685.3582.

### The Synthesis of **SY 6**

The synthesis of **SY 6** followed the same procedure as **SY 3**, using **15** (90 mg, 0.121 mmol) and 3 equiv of benzeneselenol (57 mg, 0.365 mmol). The resulting crude was purified by column chromatography on silica gel, eluting with dichloromethane/acetone from 30:1 to 10:1 to yield the title compound **SY 6** (29 mg, 28%) as a green solid. ^**1**^ **H NMR** (400 MHz, CDCl_3_) δ 8.86 (d, 2H, *J* = 14.2 Hz), 8.04 (d, 2H, *J* = 8.5 Hz), 7.93–7.89 (m, 4H), 7.58–7.54 (m, 2H), 7.45–7.41 (m, 4H), 7.37–7.34 (m, 2H), 7.27–7.23 (m, 2H), 7.13–7.09 (m, 1H), 6.24 (d, 2H, *J* = 14.2 Hz), 4.33 (q, 4H, *J* = 7.2 Hz), 2.84 (apparent t, 4H, *J* = 6.1 Hz), 2.09–2.03 (m, 2H), 1.79 (s, 12H), 1.49 (t, 6H, *J* = 7.2 Hz); ^**13**^ **C NMR** (100 MHz, CDCl_3_) δ 173.2, 150.8, 145.3, 139.2, 137.3, 134.0, 133.9, 131.9, 130.8, 130.1, 129.5, 128.1, 127.7, 126.1, 125.7, 125.1, 122.1, 110.7, 100.8, 51.0, 40.3, 31.0, 27.4, 26.8, 12.9. **HRMS** (ES *m*/*z*): [M]^+^ calc. for C_48_H_49_N_2_Se_1_: 733.30555, found: 733.3043.

### The Synthesis of **SY 7**

Compound **12** (100 mg, 0.156 mmol) and 4-mercaptobenzoic acid (72 mg, 0.47 mmol) were dissolved in anhydrous DMF (20 mL) under N_2_. The solution was stirred at room temperature until TLC inspection showed **12** was completely consumed. The DMF was removed under *vacuum* at 42 °C, and the residue was dissolved in dichloromethane (1 mL) and purified by column chromatography on silica gel (eluting using pure acetone), yielding the pure product **SY 7** as a green solid (23 mg, 19%). ^**1**^ **H NMR** (500 MHz, CDCl_3_) δ 8.62 (d, 2H, *J* = 14.1 Hz), 8.00 (d, 2H, *J* = 8.6 Hz), 7.32 (t, 2H, *J* = 8.7 Hz), 7.25 (d, 2H, *J* = 7.5 Hz), 7.18–7.10 (m, 6H), 6.15 (d, 2H, *J* = 14.1 Hz), 4.15 (q, 4H, *J* = 7.2 Hz), 2.76–2.74 (m, 4H), 2.03–2.00 (m, 2H), 1.41–1.38 (m, 18H); ^**13**^ **C NMR** (125 MHz, CDCl_3_) δ 172.1, 169.8, 151.0, 146.3, 141.8, 141.4, 141.1, 134.0, 131.8, 131.2, 128.8, 125.4, 125.2, 122.4, 110.7, 101.3, 49.4, 40.0, 27.9, 26.8, 20.9, 12.6. **HRMS** (ES *m*/*z*): [M]^+^ calc. for C_41_H_45_N_2_O_2_S_1_: 629.31963, found: 629.3177.

### The Synthesis of **SY 8**

Compound **15** (100 mg, 0.135 mmol) and 4-mercaptobenzoic acid (62 mg, 0.41 mmol) were dissolved in anhydrous DMF (20 mL) under N_2_. The resulting solution was stirred at room temperature and stopped when **15** was consumed (TLC analysis). After evaporation of the DMF, the solid residue was extracted with DCM (1 mL), which was evaporated and the crude was purified by column chromatography on silica gel, eluting using dichloromethane/acetone (1:1 to pure acetone), yielding **SY 8** a dark green solid product (25 mg, 21%). ^**1**^ **H NMR** (600 MHz, CDCl_3_) δ 8.68 (d, 2H, *J* = 14.1 Hz), 8.00–7.96 (m, 4H), 7.88 (t, 4H, *J* = 9.0 Hz), 7.53 (t, 2H, *J* = 8.0 Hz), 7.42–7.39 (m, 4H), 7.32 (d, 2H, *J* = 8.5 Hz), 6.26 (d, 2H, *J* = 14.2 Hz), 4.32 (q, 4H, *J* = 7.2 Hz), 2.87–2.84 (m, 4H), 2.10–2.08 (m, 2H), 1.71 (s, 12H), 1.46 (t, 6H, *J* = 7.0 Hz); ^**13**^ **C NMR** (125 MHz, CDCl_3_) δ 173.3, 170.1, 148.3, 144.8, 144.7, 139.2, 134.2, 134.1, 132.0, 131.2, 130.9, 130.2, 128.1, 127.8, 125.6, 125.2, 122.1, 118.1, 110.7, 101.2, 51.1, 40.5, 27.4, 27.0, 20.9, 12.8. **HRMS** (ES *m*/*z*): [M]^+^ calc. for C_49_H_49_N_2_O_2_S_1_: 729.35093, found: 729.3490.

## Conclusion

In summary, the design, synthesis, and optical characterisations of a series of eight near-infrared cyanine dyes containing different chalcogens (O, S, and Se) was conducted and their outstanding deep-NIR absorption and emission properties was demonstrated. Compared to the indole series (with λ_max, abs_/λ_max, ems_ ranging from 767 to 820 nm), the benzoindole series exhibited a more significant red-shift in both absorption and emission, ranging from 800 nm up to 850 nm. The fluorescence quantum yields ranged from 6% to 18%, with the indole series showing slightly higher quantum yields. TDDFT calculations used to investigate the absorption, emission, and energy gaps of the chalcogen-containing dyes, closely reflected the experimental data, reinforcing the observed photophysical trends. The singlet oxygen quantum yields for the selenium series, **SY 3** and **SY 6**, were both around 13%, high values considering the deep NIR excitation wavelengths were 812 and 854 nm respectively (the excitation energy at 850 nm is 141 kJ/mol, while the singlet oxygen ^1^Δ_g_ state is 95 kJ/mol above the ^3^Σ_g_^–^ ground state). Interestingly, while the change from indole to benzoindole had no observable impact on singlet oxygen generation, the selection of chalcogen did, with the Se-based dyes exhibiting the highest Φ_SO_, followed by the O and S variants. This is perhaps surprising in view of the propensity of selenium compounds to be readily oxidized, but this dye, with an absorption wavelengths λ_max,abs_ of 826 nm and an extinction coefficient of 178,000 cm^–1^ M^–1^, undergoes rapid singlet oxygen generation upon illumination at low illumination power (0.02 W/cm^2^) and in view of their ready synthesis and NIR absorption properties offer potential in photodynamic applications.

## Supplementary Material



## Notes

Computational details including DFT and TDDFT calculations. Singlet oxygen generation studies. Detailed synthetic procedures and characterization data including NMR and HRMS spectra (PDF)
