# Supplementary material for: Structure–Property Relationships of Near-Infrared Cyanine Dyes: Chalcogen-Driven Singlet Oxygen Generation with High Fluorescence Efficiency
Source: ACS Omega. 2026 Jan 12;11(3):4475–84. doi: 10.1021/acsomega.5c10499 (PMC12854524; doi:10.1021/acsomega.5c10499)
Supplement: Supplementary file 1 [file ao5c10499_si_001.pdf]

Supporting information

Structure-Property Relationships of Near-Infrared Cyanine Dyes: Chalcogen-Driven Singlet Oxygen Generation with High Fluorescence Efficiency

Shufan Yang,[a,b] Ewan Forsyth,[c] Wuyang Lin,[d] Kerry Setchfield,[e] Rachel Crespo Otero,[f] Devis Di Tommaso,[d,g] Annamaria Lilienkampf,[a] Amanda Wright,[e] Mark Bradley *[b]

[a] *School of Chemistry, University of Edinburgh, David Brewster Road, EH9 3FJ, United Kingdom*
[b] *Precision Healthcare University Research Institute, Queen Mary University of London, Empire House, 67 New Road, E1 1HH, United Kingdom. m.bradley@qmul.ac.uk*
[c] *Department of Chemistry, University of Oxford, Mansfield Rd, OX1 3TA, Oxford, United Kingdom*
[d] *School of Physical and Chemical Sciences, Queen Mary University of London, 327 Mile End Road London, E1 4NS, United Kingdom*
[e] *Electrical and Electronic Engineering, University of Nottingham, Life Sciences Building, Nottingham, NG7 2RD, United Kingdom*
[f] *Department of Chemistry, University College London, 20 Gordon St, London WC1H 0AJ, United Kingdom*
[g] *Digital Environment Research Institute, Queen Mary University of London, Empire House, 67 New Road, E1 1HH, United Kingdom*

Table of Contents

1. Computational Details 2

1.1 A list of the predicted transitions of TD-DFT calculations 3

1.2 Coordinates of the DFT-optimised geometries of SY 1-SY 8 7

1.3 Energies of the first five triplet states (T_1-T_5, in eV) at the S1-optimised geometry for SY 1 - SY 6. 26

2. Singlet oxygen evaluation of SY 1 - SY 8 27

Quantifying singlet oxygen generation of NIR cyanine dyes 27

2.1. Singlet oxygen evaluation of SY 1 – SY 8 over time. 29

3. Oxidation test of SY 5 (S) and SY 6 (Se) with H_2O_2 (5 %) 30

4. Synthetic procedures: 31

Synthesis of 1-ethyl-2,3,3-trimethyl-3H-indol-1-ium (10)[9] 31

Synthesis of 2-((E)-2-((E)-2-chloro-3-(2-((E)-1-ethyl-3,3-dimethylindolin-2-ylidene)ethylidene)cyclohex-1-en-1-yl)vinyl)-1-ethyl-3,3-dimethyl-3H-indol-1-ium (12) [10] 31

Synthesis of 3-ethyl-1,1,2-trimethyl-1H-benzo[e]indol-3-ium (14)[11] 31

Synthesis of 2-((E)-2-((E)-2-chloro-3-((E)-2-(3-ethyl-1,1-dimethyl-1,3-dihydro-2H-benzo[e]indol-2-ylidene)ethylidene)cyclohex-1-en-1-yl)vinyl)-3-ethyl-1,1-dimethyl-1H-benzo[e]indol-3-ium (15)[12] 32

5. Proton NMR and Carbon NMR of SY 1 to SY 8 33
6. Mass spec of SY 1 to SY 8 41
7. Non-normalised UV–vis spectra of the SY 1 – SY 8 49
8. References 50

1. Computational Details

All density functional theory (DFT) calculations were conducted using the Gaussian 16 program.[1] The Becke-type three-parameter Lee–Yang–Parr (B3LYP) exchange correlation functional[2-4] and triple-zeta Pople-type basis set with polarisation and diffusion (6-311+G (2d, p))[5, 6] were used in the work. Polarizable Continuum Model (PCM)[7] was chosen to address the solvent effect (ethanol). Geometry optimizations were deemed converged when the maximum and root-mean-square (RMS) forces reached 0.0045 and 0.0030 Ha/Bohr, respectively, and the maximum and RMS displacements reached 0.0018 and 0.0012 Å, respectively. No imaginary frequencies were observed, confirming that the optimised structures correspond to true local minima on the potential energy surface.

Time-dependent DFT (TD-DFT) calculations were subsequently carried out at the same level of theory (B3LYP/6-311+G(2d,p)) within the PCM model for ethanol to evaluate electronic excitation energies and oscillator strengths. The five lowest singlet excited states were computed from the optimized ground-state geometries, and the corresponding orbital transitions were analyzed. Excitation energies, wavelengths, oscillator strengths (f), and dominant molecular orbital transitions (DMOT) were extracted from the TD-DFT outputs. The contribution of each DMOT was quantified by squaring the TD-DFT configuration interaction coefficients. A complete list of the most significant transition contributions for each state is provided in the Supporting Information (Table S1). All visualisations were generated using GaussView 6.0All visualisation was done with the help of GaussView 6.0.[8]

1.1 A list of the predicted transitions of TD-DFT calculations

Table S1. Details of the five calculated states in TD-DFT for **SY 1**. f denotes oscillator strength, DMOT means dominant molecular orbital transitions.

State	Energy (eV)	Wavelength (nm)	f	DMOT	Contribution (%)
1	1.9449	637.47	2.0293	153 (HOMO) →154 (LUMO)	48.06
2	3.4368	360.76	0.0361	151→154	30.09
3	3.5150	352.73	0.1014	152→154	28.12
4	4.0665	304.89	0.1463	153→155	45.09
5	4.2965	288.57	0.0172	150→154	44.27

Table S2. Details of the five calculated states in TD-DFT for **SY 2**. f denotes oscillator strength, DMOT means dominant molecular orbital transitions.

State	Energy (eV)	Wavelength (nm)	f	DMOT	Contribution (%)
1	1.8514	669.69	1.8088	157 (HOMO) →158 (LUMO)	50.07
2	2.3316	531.77	0.1612	156→158	45.53
3	2.9486	420.48	0.0658	155→158	42.34
4	3.4119	363.29	0.0050	154→158	42.77
5	3.4662	357.70	0.1051	157→159	41.94

Table S3. Details of the five calculated states in TD-DFT for **SY 3**. f denotes oscillator strength, DMOT means dominant molecular orbital transitions.

State	Energy (eV)	Wavelength (nm)	f	DMOT	Contribution (%)
1	1.8665	664.28	2.0008	166 (HOMO)→ 167 (LUMO)	48.15
2	2.5598	484.34	0.0371	165→167	46.83
3	3.4915	355.10	0.0628	164→167	43.68
4	3.9978	310.13	0.0893	166→168	44.64
5	4.2098	294.51	0.0484	163→167	44.50

Table S4. Details of the five calculated states in TD-DFT for **SY 4**. f denotes oscillator strength, DMOT means dominant molecular orbital transitions.

State	Energy (eV)	Wavelength (nm)	f	DMOT	Contribution (%)
1	1.8241	679.68	2.0758	179 (HOMO)→ 180 (LUMO)	50.01
2	2.5753	481.44	0.0240	178→180	47.76
3	2.7564	449.80	0.0007	176→180	32.18
4	2.8816	430.27	0.0618	177→180	29.06
5	3.0206	410.46	0.0459	179→181	47.29

Table S5. Details of the five calculated states in TD-DFT for **SY 5**. f denotes oscillator strength, DMOT means dominant molecular orbital transitions.

State	Energy (eV)	Wavelength (nm)	f	DMOT	Contribution (%)
1	1.7552	706.39	1.9303	183 (HOMO)→ 184 (LUMO)	49.96
2	2.2838	542.89	0.1139	182→184	36.19
3	2.5977	477.29	0.0216	181→184	33.57
4	2.8077	441.58	0.0531	180→184	43.81
5	3.0045	412.66	0.0396	183→185	46.06

Table S6. Details of the five calculated states in TD-DFT for **SY 6**. f denotes oscillator strength, DMOT means dominant molecular orbital transitions.

State	Energy (eV)	Wavelength (nm)	f	DMOT	Contribution (%)
1	1.7580	705.24	1.9268	192 (HOMO)→ 193 (LUMO)	49.44
2	2.0762	597.17	0.1136	191→193	42.39
3	2.5520	485.83	0.0195	190→193	41.63
4	2.8056	441.92	0.0511	189→193	46.57
5	3.0001	413.27	0.0379	192→194	47.04

Table S7. Details of the five calculated states in TD-DFT for **SY 7**. f denotes oscillator strength, DMOT means dominant molecular orbital transitions.

State	Energy (eV)	Wavelength (nm)	f	DMOT	Contribution (%)
1	1.8422	673.02	1.8735	168 (HOMO)→ 169 (LUMO)	50.39
2	2.4543	505.17	0.1153	167→169	39.52
3	2.8057	441.90	0.0587	168→170	49.38
4	2.9304	423.09	0.0373	166→169	36.74
5	3.3997	364.69	0.0012	165→169	46.06

Table S8. Details of the five calculated states in TD-DFT for **SY 8**. f denotes oscillator strength, DMOT means dominant molecular orbital transitions.

State	Energy (eV)	Wavelength (nm)	f	DMOT	Contribution (%)
1	1.7410	712.16	1.9818	194 (HOMO)→ 195 (LUMO)	50.11
2	2.3901	518.75	0.0722	193→195	35.24
3	2.5849	479.64	0.0144	191→195	23.19
4	2.7465	451.42	0.0340	194→196	48.98
5	2.7669	448.09	0.0764	192→195	30.89

1.2 Coordinates of the DFT-optimised geometries of SY 1-SY 8

SY 1:

opt=loose b3lyp/6-311+g(2d,p) scrf=(solvent=ethanol,pcm) nosymm integral=grid=finegrid scf=(maxcycle=100,tight)

Title Card Required

1 1

C	-3.87398242	-1.94878344	-2.22500180
C	-4.03418469	-3.03953027	-3.27278564
C	-3.69386275	-2.53851520	-4.67215204
C	-2.28040604	-1.96179431	-4.70478545
C	-2.03171316	-0.97137226	-3.58632286
C	-2.83348766	-1.02527493	-2.43019401
O	-2.60676983	-0.06161337	-1.45743924
C	-1.02667666	-0.00519128	-3.68862497
C	-0.14165815	0.12973978	-4.75823604
C	-4.78576634	-1.95616174	-1.16260432
C	-4.97033358	-1.09442220	-0.07801712
N	-6.16948905	-0.33598843	1.87047364
C	-5.99225791	-1.22318882	0.85808444
C	-7.08052055	-2.30971445	0.96073546
C	-7.83141836	-1.86238972	2.20064463
C	-7.25336058	-0.69710891	2.69386842
C	-8.92731729	-2.41427403	2.83812424
C	-9.43367113	-1.78328224	3.97807261
C	-8.84257702	-0.61781821	4.46143035
C	-7.73703214	-0.05250880	3.82554560
C	-8.03178582	-2.28880300	-0.25844371
N	1.64453825	1.18909989	-5.97495810
C	2.55838245	2.25599572	-5.86391745
C	2.33061346	2.92596789	-4.66534527
C	1.19430322	2.25004729	-3.92137897
C	0.83503835	1.11199267	-4.89055149
C	1.69534502	1.71166265	-2.56241138
C	3.54877650	2.64775810	-6.75533182
C	4.31814171	3.75874302	-6.40941024
C	4.09903172	4.44325246	-5.21587097
C	3.09874233	4.02672904	-4.33277575
C	-6.47882909	-3.71534443	1.18463253
C	0.01731109	3.23465317	-3.73582888
C	1.62176496	0.29097784	-7.13322470
C	2.52160430	-0.93070425	-6.95830897
C	-5.34843176	0.84618080	2.14538444
C	-4.21491394	0.56338482	3.12911756
C	-1.66356085	-0.29501774	-0.46952908
H	-3.37813319	-3.88298575	-3.02335077
H	-5.05549430	-3.42369747	-3.24567878
H	-4.41169854	-1.76576598	-4.96502345
H	-3.78141425	-3.35007230	-5.39787957
H	-2.10813855	-1.47830224	-5.66846033
H	-1.54942831	-2.77676564	-4.63643066

H	-0.94616375	0.68822736	-2.86602466
H	-0.21183238	-0.59244789	-5.55889624
H	-5.48808460	-2.77601712	-1.23323957
H	-4.30069414	-0.25855554	0.03552408
H	-9.39269725	-3.31973632	2.46665592
H	-10.29059415	-2.20365327	4.48942697
H	-9.24319600	-0.13973362	5.34685550
H	-7.28303071	0.84933238	4.21304769
H	-7.53048960	-2.60046615	-1.17363008
H	-8.86212848	-2.97375938	-0.07883715
H	-8.44224136	-1.29017395	-0.41495488
H	0.90201700	1.21552404	-2.00507307
H	2.06215467	2.54350635	-1.95882107
H	2.51459880	1.00443746	-2.69983965
H	3.73182399	2.12190073	-7.68259334
H	5.09878017	4.09005314	-7.08303986
H	4.70930793	5.30315959	-4.96963717
H	2.93462354	4.56373670	-3.40588364
H	-5.94036573	-4.07388438	0.30820463
H	-5.79214694	-3.71489761	2.03241770
H	-7.28246565	-4.42207644	1.39783773
H	0.34991132	4.08622339	-3.14011205
H	-0.33466157	3.60838190	-4.69848043
H	-0.82191501	2.76892588	-3.22081700
H	0.58945105	-0.00357588	-7.31441721
H	1.93133598	0.87666717	-7.99799679
H	2.47373726	-1.54785030	-7.85710515
H	3.56012047	-0.63488792	-6.80244791
H	2.20524899	-1.53789459	-6.10888476
H	-4.96275993	1.22309514	1.20042856
H	-6.01568485	1.61456826	2.53603586
H	-3.65647154	1.48275631	3.31274156
H	-4.60160979	0.20295306	4.08352456
H	-3.52460804	-0.18089797	2.73067327
C	-1.38634423	0.78300484	0.36833318
C	-0.45688832	0.63474450	1.39009114
C	0.19663017	-0.58084529	1.58039982
C	-0.09001046	-1.64849941	0.73629862
C	-1.01940417	-1.51583457	-0.29296885
H	-1.89855117	1.72366094	0.20837106
H	-0.24269539	1.47546717	2.03897168
H	0.92114476	-0.69325604	2.37722784
H	-1.23351262	-2.35142609	-0.94539979
H	0.41126750	-2.59925152	0.87328246

SY 2:
# opt=loose b3lyp/6-311+g(2d,p) scrf=(solvent=ethanol,pcm) nosymm
integral=grid=finegrid scf=(maxcycle=100,tight)

Title Card Required

1 1

C	-3.51159730	-1.77997564	-1.97012140
C	-3.47450896	-2.94692792	-2.94986251
C	-3.58879736	-2.41624404	-4.38175389
C	-2.39119458	-1.51604066	-4.69212150
C	-1.95126849	-0.63418907	-3.53022042
C	-2.59978285	-0.72615188	-2.27189920
C	-0.87537404	0.25424925	-3.73957669
C	-0.21783246	0.45379981	-4.95541200
C	-4.47498336	-1.86178943	-0.95295467
C	-4.94623361	-0.93890537	-0.00116088
N	-6.52595338	-0.21920230	1.68607359
C	-6.00986004	-1.18361904	0.86738797
C	-6.78832877	-2.49661578	1.10734287
C	-7.74667655	-2.08130947	2.21189440
C	-7.55264493	-0.72903852	2.50610474
C	-8.71587765	-2.80723678	2.89273999
C	-9.48464439	-2.15949263	3.87304447
C	-9.27838877	-0.80421562	4.15505165
C	-8.30417642	-0.06239308	3.47398740
C	-7.60128531	-2.92840582	-0.14185710
N	1.45127296	1.47319160	-6.37328246
C	2.55722114	2.35104785	-6.30828203
C	2.70414220	2.79851112	-4.99260316
C	1.62770391	2.16872254	-4.12510184
C	0.87719220	1.30470296	-5.15516319
C	2.27439694	1.30459956	-3.01322213
C	3.40947075	2.75623313	-7.33455945
C	4.43582049	3.64900015	-6.99988585
C	4.59489517	4.11041545	-5.68789590
C	3.72495435	3.68464168	-4.67200540
C	-5.86160203	-3.63622023	1.59670743
C	0.71447812	3.26649490	-3.52210958
C	1.03497239	0.82050759	-7.62238869
C	1.70493878	-0.54039545	-7.83252333
C	-6.10584708	1.18436488	1.75703724
C	-5.10619528	1.45160577	2.88582036
C	-1.49803492	-0.30718975	0.31180357
H	-2.53043698	-3.50192297	-2.84594166
H	-4.28374411	-3.64897624	-2.73111664
H	-4.52546484	-1.85413918	-4.48030045
H	-3.62920007	-3.23963696	-5.10285071
H	-2.61768980	-0.88267100	-5.55900859
H	-1.53589785	-2.13863631	-4.98861180

H	-0.53697124	0.80396361	-2.87211674
H	-0.57439658	-0.08989842	-5.82126328
H	-4.99251437	-2.81382768	-0.96277570
H	-4.48857869	0.03974893	0.03678516
H	-8.88246852	-3.85866427	2.67651777
H	-10.24371280	-2.71414928	4.41562652
H	-9.87863294	-0.31498917	4.91602035
H	-8.14884152	0.98512990	3.70591777
H	-6.95714774	-3.24987385	-0.96252332
H	-8.25060913	-3.76758472	0.12462262
H	-8.23192425	-2.10942513	-0.49954921
H	1.52168587	0.82096341	-2.38761666
H	2.88942357	1.94315010	-2.37251573
H	2.91609045	0.52999078	-3.44261272
H	3.30089508	2.40217437	-8.35328788
H	5.11812706	3.98371194	-7.77498409
H	5.39855670	4.80126594	-5.45350022
H	3.85489549	4.04491897	-3.65568443
H	-5.15334992	-3.94267933	0.82391257
H	-5.29460719	-3.32632136	2.47923836
H	-6.46645052	-4.50689577	1.86687372
H	1.31830347	3.93509905	-2.90184639
H	0.24475547	3.86160354	-4.31034487
H	-0.07159009	2.84172739	-2.89442052
H	-0.05267674	0.72962648	-7.61194564
H	1.28086760	1.50386513	-8.43754078
H	1.36841368	-0.96607448	-8.78243063
H	2.79372036	-0.44283024	-7.86696288
H	1.44501949	-1.23855049	-7.03183789
H	-5.68921821	1.46509927	0.78880748
H	-7.01086040	1.78330387	1.89305004
H	-4.83151507	2.51089794	2.88560479
H	-5.53534055	1.21036912	3.86237304
H	-4.19768703	0.85887674	2.75387417
C	-1.51804505	0.34125175	1.55546932
C	-0.89217185	-0.24449298	2.65929716
C	-0.25447254	-1.48226533	2.53493034
C	-0.23632124	-2.12567095	1.29302164
C	-0.84624651	-1.54116679	0.18001106
H	-2.01606380	1.30050726	1.66082737
H	-0.91261896	0.26675916	3.61727391
H	0.22534555	-1.93946451	3.39480077
H	-0.81193214	-2.04419856	-0.78036442
H	0.26279539	-3.08406792	1.18264391
S	-2.23378143	0.56823819	-1.07378885

SY 3:
opt=loose b3lyp/6-311+g(2d,p) scrf=(solvent=ethanol,pcm) nosymm integral=grid=finegrid scf=(maxcycle=100,tight)

Title Card Required

1 1

C	-3.74988849	-1.82819801	-2.15526658
C	-3.72110229	-3.06212694	-3.05914705
C	-3.54757856	-2.67843415	-4.52638403
C	-2.22776648	-1.93331433	-4.69837859
C	-2.02368818	-0.84087518	-3.65971012
C	-2.84645743	-0.79126030	-2.50006771
C	-0.97901442	0.08008875	-3.85493914
C	-0.06817416	0.08080219	-4.91610099
C	-4.68386482	-1.90942244	-1.09877961
C	-5.03731566	-1.05433048	-0.04689721
N	-6.35903230	-0.44605253	1.87933647
C	-6.03157673	-1.33030441	0.89620167
C	-6.92143664	-2.58189167	1.04340630
C	-7.75060511	-2.21989588	2.26420996
C	-7.37532329	-0.95222623	2.71648138
C	-8.74935668	-2.92421773	2.92475654
C	-9.36282742	-2.34033167	4.04431304
C	-8.97293010	-1.07057644	4.48607679
C	-7.96678439	-0.35180121	3.82776430
C	-7.84892566	-2.78027159	-0.18315713
N	1.89623625	0.89622866	-6.06442038
C	2.87001373	1.91534850	-5.97715895
C	2.62326917	2.68254704	-4.83512340
C	1.39043727	2.14772695	-4.12651653
C	1.00380233	0.97030797	-5.04217157
C	1.74804531	1.67946944	-2.69274538
C	3.93380849	2.18060331	-6.83883816
C	4.76936884	3.25703680	-6.51323773
C	4.53990411	4.03192333	-5.37029049
C	3.45844514	3.74719651	-4.52175053
C	-6.08658079	-3.85638193	1.32748544
C	0.28286425	3.23264977	-4.09560468
C	1.87955370	-0.08903525	-7.15356950
C	1.02244286	0.35279500	-8.34317747
C	-5.73421796	0.86058331	2.12004831
C	-4.58215768	0.78358322	3.12621217
C	-1.65801864	0.37650103	0.01840910
H	-2.88988408	-3.71913692	-2.76117842
H	-4.63933064	-3.63942860	-2.91889173
H	-4.38485530	-2.04179385	-4.83930298
H	-3.56163158	-3.56949844	-5.16288213
H	-2.17854325	-1.48683711	-5.69817374
H	-1.39257148	-2.64633608	-4.64045550

H	-0.87318042	0.83857880	-3.09322480
H	-0.17989524	-0.66675694	-5.69197929
H	-5.23422898	-2.84255777	-1.12837740
H	-4.53169547	-0.10572678	0.03868186
H	-9.05618656	-3.90980878	2.58688247
H	-10.14465452	-2.87805006	4.57134261
H	-9.45390486	-0.63140468	5.35460657
H	-7.66990928	0.62851129	4.18257373
H	-7.28700319	-3.03676319	-1.08356425
H	-8.54739200	-3.59650941	0.02296223
H	-8.42958445	-1.87554777	-0.38460194
H	0.86951444	1.31012357	-2.16050798
H	2.15467743	2.52386920	-2.12874603
H	2.50155325	0.88699168	-2.71303339
H	4.11736947	1.59153516	-7.73013997
H	5.60684521	3.49070004	-7.16326255
H	5.20154438	4.86079725	-5.13935555
H	3.28471472	4.35347241	-3.63751601
H	-5.47111357	-4.13923219	0.47116448
H	-5.43087536	-3.71065245	2.19059014
H	-6.76233908	-4.68804130	1.54753782
H	0.64716195	4.10346435	-3.54264789
H	0.02025106	3.55467267	-5.10730025
H	-0.62161486	2.87145262	-3.60118798
H	2.91600990	-0.24500187	-7.45991021
H	1.53032107	-1.03962764	-6.74587738
H	1.06249675	-0.41375575	-9.12276288
H	-0.02206893	0.49044804	-8.04979584
H	1.38991457	1.29197621	-8.76636385
H	-5.39531554	1.26183709	1.16406520
H	-6.51960378	1.53061004	2.47849194
H	-4.15438531	1.78075480	3.26538634
H	-4.92955910	0.42124934	4.09796554
H	-3.79083914	0.11874407	2.76984949
C	-1.37798448	1.39191954	0.94358840
C	-0.54896447	1.12718804	2.03515200
C	0.00075067	-0.14706172	2.21585136
C	-0.28569797	-1.15481022	1.29084459
C	-1.10779296	-0.89702476	0.18890283
H	-1.79785735	2.38571414	0.81363734
H	-0.33316334	1.91996551	2.74581397
H	0.64340045	-0.35017183	3.06690486
H	-1.31718540	-1.68784497	-0.52318407
H	0.13449737	-2.14840158	1.41882013
Se	-2.77499310	0.87296218	-1.47417073

SY 4:
opt=loose b3lyp/6-311+g(2d,p) scrf=(solvent=ethanol,pcm) nosymm integral=grid=finegrid scf=(maxcycle=100,tight)

Title Card Required

1 1

C	-3.73346805	-1.95849731	-2.15833566
C	-3.75715214	-3.12717884	-3.13736364
C	-3.31732617	-2.70774468	-4.54011694
C	-1.92832873	-2.06545862	-4.49674960
C	-1.83088820	-0.97173624	-3.44886483
C	-2.72873887	-0.98798139	-2.35600804
O	-2.65115006	0.06817272	-1.45379046
C	-0.87352010	0.04908757	-3.54897001
C	0.09694586	0.15340723	-4.55436786
C	-4.71761090	-1.95592085	-1.15362956
C	-5.01358856	-1.04339262	-0.13012008
N	-6.35175807	-0.23649695	1.71482249
C	-6.07848559	-1.17379635	0.76755786
C	-7.11442033	-2.30990181	0.89653605
C	-7.95124092	-1.83122268	2.08076181
C	-7.45120657	-0.61587465	2.51177572
C	-9.07607267	-2.40865132	2.73141217
C	-9.64928974	-1.68091640	3.83819499
C	-9.08660600	-0.43603013	4.23849736
C	-7.99761055	0.10805206	3.59467002
C	-7.98729011	-2.39412608	-0.38482676
N	1.96873649	1.17777640	-5.68402941
C	2.76428062	2.34179642	-5.63877820
C	2.37766042	3.13605883	-4.57386066
C	1.22245047	2.45082757	-3.84794927
C	1.03763347	1.17776184	-4.69401170
C	1.62554036	2.08006554	-2.39567440
C	3.80988917	2.68597566	-6.52358632
C	4.47166580	3.87213313	-6.29537397
C	4.12396251	4.72853742	-5.21272846
C	3.05029304	4.36278577	-4.32068696
C	-6.41157939	-3.65367178	1.22437753
C	-0.06131318	3.32026758	-3.89467787
C	2.13876643	0.13566765	-6.70517217
C	1.26674413	0.36358585	-7.94326611
C	-5.60817222	1.00836035	1.94730271
C	-4.49598438	0.84754666	2.98729754
C	-1.75072563	0.02023312	-0.39799461
H	-3.08844134	-3.92188296	-2.77412558
H	-4.76254737	-3.55896019	-3.16776563
H	-4.04016725	-1.99020495	-4.94906977
H	-3.30694628	-3.57400370	-5.20994028
H	-1.68760792	-1.65470215	-5.48221862

H	-1.17197211	-2.83746967	-4.29152411
H	-0.90179261	0.80874875	-2.78011508
H	0.12717873	-0.63185346	-5.29920531
H	-5.36858765	-2.82060489	-1.21831483
H	-4.39344725	-0.16546351	-0.03190638
H	-7.40121377	-2.69577357	-1.25460872
H	-8.78856263	-3.12332178	-0.25041327
H	-8.44523243	-1.42465183	-0.60145266
H	0.82529492	1.54647310	-1.87997654
H	1.84931191	2.98276296	-1.82361229
H	2.51780558	1.44740706	-2.39265206
H	4.08678627	2.05218878	-7.35775403
H	5.28045268	4.17198906	-6.95538537
H	-5.77976393	-3.98982219	0.40030979
H	-5.78506178	-3.55214717	2.11515503
H	-7.15089848	-4.43279609	1.41911417
H	0.09627066	4.26049517	-3.36278306
H	-0.33000477	3.55610628	-4.92840158
H	-0.90608228	2.80811979	-3.43037529
H	3.19640318	0.11725212	-6.97451905
H	1.92303298	-0.82888937	-6.24162941
H	1.44554167	-0.43897756	-8.66505181
H	0.20380345	0.36352801	-7.68594139
H	1.50569146	1.31647633	-8.42434871
H	-5.20815864	1.34978917	0.99163939
H	-6.33229366	1.76171579	2.26566208
H	-3.99407551	1.80849759	3.13347468
H	-4.89964061	0.52185194	3.95033394
H	-3.75066174	0.11886884	2.65786047
C	-1.59110485	1.21244921	0.31620662
C	-0.71450686	1.24978699	1.40176815
C	0.00134837	0.10695192	1.77618035
C	-0.17055268	-1.07674660	1.05346523
C	-1.04632122	-1.13138184	-0.03652684
H	-2.14942213	2.09247882	0.01299788
H	-0.59064954	2.17708839	1.95327956
H	-1.17384883	-2.05371507	-0.59212699
H	0.37738041	-1.97137501	1.33447504
C	2.73946335	5.25416039	-3.25065710
C	3.44049068	6.42863705	-3.07402715
C	4.49308251	6.78433089	-3.95343482
C	4.82316726	5.94823274	-4.99824116
H	5.03526529	7.71255250	-3.80143725
H	5.62874764	6.21039927	-5.67900757
H	1.94117706	5.01348411	-2.55971908
H	3.18218720	7.08839910	-2.25097598
H	-7.58093659	1.05438891	3.91882586
H	-9.53371913	0.09013759	5.07692605
C	-9.67670393	-3.65134925	2.36911898
C	-10.76821969	-4.14317596	3.05376303

C	-11.32669854	-3.42553760	4.14044086
C	-10.77466428	-2.22097632	4.51959271
H	-12.18595275	-3.82610916	4.66946432
H	-11.19439603	-1.66136309	5.35130350
H	-9.27610336	-4.22608474	1.54366556
H	-11.20456285	-5.09198295	2.75575243
H	0.68368038	0.13957451	2.61968615

SY 5:
opt=loose b3lyp/6-311+g(2d,p) scrf=(solvent=ethanol,pcm) nosymm integral=grid=finegrid scf=(maxcycle=100,tight)

Title Card Required

1 1

C	-3.50012040	-1.69092149	-1.89892014
C	-3.31025809	-2.92949118	-2.76708751
C	-3.33252112	-2.52842815	-4.24473353
C	-2.16620723	-1.58176758	-4.53494253
C	-1.88297529	-0.57759660	-3.42504911
C	-2.63500271	-0.60506855	-2.22224465
S	-2.46034119	0.81359068	-1.12433467
C	-0.84183565	0.35443841	-3.62200905
C	-0.08267613	0.48124107	-4.78720466
C	-4.52894980	-1.76184247	-0.94586830
C	-5.13166629	-0.80257868	-0.11242766
N	-6.86511906	-0.05588562	1.40085551
C	-6.22656987	-1.04863844	0.71795131
C	-6.91451215	-2.38877873	1.05936228
C	-7.96966835	-1.94495454	2.07077662
C	-7.88998653	-0.57187439	2.21750755
C	-8.94460821	-2.68226769	2.79744916
C	-9.81750217	-1.94314178	3.67788789
C	-9.68579313	-0.53014280	3.78781264
C	-8.73816347	0.16713369	3.07193538
C	-7.60288099	-2.98887080	-0.19782562
N	1.69629019	1.40612172	-6.13202225
C	2.68798850	2.41024987	-6.09150531
C	2.66130854	3.04779284	-4.86443387
C	1.55221367	2.42910896	-4.01742220
C	0.98532222	1.36898517	-4.97908757
C	2.13350620	1.74809307	-2.74918913
C	3.59084574	2.74572644	-7.12367242
C	4.49002697	3.75880641	-6.87554258
C	4.51751605	4.44814557	-5.63003174
C	3.58441715	4.09368110	-4.58787444
C	-5.89819522	-3.37071692	1.69657904
C	0.46505321	3.48042079	-3.67067851
C	1.48538062	0.54907766	-7.30752849
C	0.48191504	1.13895828	-8.30203950
C	-6.56541481	1.37917181	1.33591229
C	-5.64552304	1.84983373	2.46564439
C	-1.75866711	0.12873514	0.38071926
H	-2.34681449	-3.40961291	-2.53994809
H	-4.09137464	-3.66413022	-2.55314131
H	-4.28822587	-2.03860969	-4.46782369
H	-3.26292738	-3.40983685	-4.89115486
H	-2.35088233	-1.03997952	-5.47093354

H	-1.25425215	-2.17146984	-4.70239552
H	-0.61732895	0.99964065	-2.78435074
H	-0.33454764	-0.15563009	-5.62588834
H	-4.97443564	-2.74879453	-0.90764382
H	-4.75177478	0.20884382	-0.14165094
H	-6.87566323	-3.31740682	-0.94204092
H	-8.20844080	-3.85347179	0.08101938
H	-8.26225363	-2.25148917	-0.66459805
H	1.34974119	1.27161984	-2.15762787
H	2.62990670	2.48646803	-2.11670395
H	2.86913239	0.98623256	-3.02253895
H	3.58249485	2.23753052	-8.08063296
H	5.19972230	4.04801837	-7.64511870
H	-5.09928784	-3.62518162	0.99737667
H	-5.44277809	-2.92858847	2.58742128
H	-6.39322969	-4.29730738	1.99391807
H	0.89474907	4.28694152	-3.07358397
H	0.04724817	3.91924167	-4.58125900
H	-0.35143240	3.03745708	-3.09747242
H	2.45962111	0.40465360	-7.77830378
H	1.16476204	-0.43305781	-6.95631487
H	0.37736815	0.46081015	-9.15409520
H	-0.50224130	1.26904600	-7.84319816
H	0.82010676	2.10946759	-8.67614374
H	-6.12697405	1.58946229	0.35955348
H	-7.52037432	1.91048394	1.36721157
H	-5.44785062	2.92005789	2.35135946
H	-6.10392378	1.69025679	3.44570636
H	-4.69154579	1.31737488	2.44211163
C	-1.89075863	0.89580128	1.54811994
C	-1.30218459	0.46355428	2.73957986
C	-0.58970029	-0.73865117	2.78028011
C	-0.45818105	-1.49951507	1.61412749
C	-1.02997453	-1.06801127	0.41425877
H	-2.44626273	1.82878325	1.52680823
H	-1.41033266	1.06621834	3.63662066
H	-0.90477754	-1.66009726	-0.48573501
H	0.10071994	-2.43077279	1.63059929
C	3.65273757	4.81332252	-3.35790391
C	4.57924804	5.81760698	-3.16980188
C	5.49235093	6.16339685	-4.19647216
C	5.45759039	5.48974749	-5.39841139
H	6.21549689	6.95649416	-4.03305074
H	6.15367607	5.74628489	-6.19253218
H	2.97069493	4.57437209	-2.55163254
H	4.60879263	6.34915738	-2.22328405
H	-8.65226509	1.24260442	3.17257171
H	-10.35391952	0.00305221	4.45795646
C	-9.12115275	-4.09627684	2.71886536
C	-10.09110740	-4.73748386	3.46078721

C	-10.94331195	-4.00763959	4.32629484
C	-10.80443981	-2.64016792	4.42799450
H	-11.70152008	-4.52676607	4.90450156
H	-11.45301193	-2.06931884	5.08731195
H	-8.48686376	-4.68563241	2.06869718
H	-10.20197220	-5.81488569	3.38075624
H	-0.13940846	-1.07706271	3.70834974

SY 6:

opt=loose b3lyp/6-311+g(2d,p) scrf=(solvent=ethanol,pcm) nosymm integral=grid=finegrid scf=(maxcycle=100,tight)

Title Card Required

1 1

C	-3.67392706	-1.68161375	-2.06516341
C	-3.51813140	-2.92142244	-2.94404449
C	-3.45432288	-2.52752679	-4.42030612
C	-2.23054463	-1.64456092	-4.65494626
C	-2.02262031	-0.59014916	-3.57664714
C	-2.81565787	-0.60048767	-2.39878900
Se	-2.72951664	1.00940349	-1.29632533
C	-0.99390450	0.35431680	-3.76031600
C	-0.14426874	0.43827970	-4.86597905
C	-4.66647998	-1.78045371	-1.06902924
C	-5.19955473	-0.85993055	-0.15487750
N	-6.77831790	-0.13910606	1.51678063
C	-6.24738691	-1.11652349	0.73140616
C	-6.98760036	-2.43538265	1.03078717
C	-7.95353056	-2.00326542	2.13228864
C	-7.77848269	-0.65086015	2.36648678
C	-8.92551428	-2.73234627	2.87106652
C	-9.69199721	-2.00833281	3.85678745
C	-9.46131514	-0.61794290	4.05659406
C	-8.51698082	0.07185381	3.32939081
C	-7.77330665	-2.91005488	-0.22235010
N	1.74846073	1.33364309	-6.07109362
C	2.73036686	2.34238912	-5.96639903
C	2.57718374	3.02602632	-4.77359095
C	1.38339486	2.43936637	-4.02444440
C	0.92471042	1.33434760	-4.99431520
C	1.81382333	1.82521175	-2.66531008
C	3.73481179	2.64324379	-6.91193431
C	4.60211202	3.66993176	-6.61181339
C	4.49883611	4.40793444	-5.39874054
C	3.46303080	4.08897508	-4.44575319
C	-5.99462084	-3.51034780	1.54302455
C	0.26498922	3.50253651	-3.85394268
C	1.65800891	0.42975288	-7.22629608
C	0.74569355	0.96691205	-8.33232564
C	-6.38935590	1.27672468	1.51554132
C	-5.28458980	1.59437387	2.52624479
C	-1.74024111	0.38549099	0.23862227
H	-2.59457832	-3.45702441	-2.67705908
H	-4.34648492	-3.61218207	-2.76403765
H	-4.37117746	-1.98932990	-4.69113600
H	-3.40042244	-3.41644898	-5.05779066
H	-2.31058359	-1.14634801	-5.62874891

H	-1.33072696	-2.27370175	-4.70855979
H	-0.85441483	1.05693797	-2.95171480
H	-0.30539373	-0.24693442	-5.68913312
H	-5.12657374	-2.76180472	-1.05805923
H	-4.78988166	0.13860558	-0.14585135
H	-7.10310764	-3.19358882	-1.03573538
H	-8.38880017	-3.77784820	0.02313134
H	-8.43470503	-2.11763052	-0.58451668
H	0.95930983	1.40261356	-2.13437523
H	2.26098623	2.58981106	-2.02733785
H	2.55344789	1.03333614	-2.81452850
H	3.82631011	2.09854188	-7.84422487
H	5.38754389	3.93288602	-7.31430323
H	-5.26547178	-3.77735503	0.77564247
H	-5.45063430	-3.14489371	2.41878507
H	-6.52738348	-4.41883188	1.83057364
H	0.62191397	4.33193748	-3.24032348
H	-0.03638067	3.90475628	-4.82531962
H	-0.61654493	3.08004616	-3.36825843
H	2.67310015	0.27986578	-7.59870346
H	1.31484461	-0.54252556	-6.86851733
H	0.72784857	0.25564990	-9.16342216
H	-0.27827115	1.10013458	-7.97193185
H	1.10673059	1.92737205	-8.71114119
H	-6.08076873	1.54108739	0.50234771
H	-7.28908910	1.85864237	1.72805444
H	-5.05633544	2.66394162	2.48874529
H	-5.59633945	1.34652774	3.54500696
H	-4.36996582	1.04070659	2.29934030
C	-1.56665375	1.30906968	1.27895344
C	-0.80322904	0.96327402	2.39606948
C	-0.21720015	-0.30385217	2.48926775
C	-0.39398614	-1.21910919	1.44758190
C	-1.14809460	-0.87835598	0.32025514
H	-2.01740522	2.29589039	1.21956472
H	-0.67082965	1.68621498	3.19597605
H	-1.26771740	-1.59607306	-0.48384244
H	0.05951561	-2.20462759	1.50465809
C	3.39845161	4.86089156	-3.24768524
C	4.29762096	5.87825556	-3.00526405
C	5.31390618	6.18668102	-3.94318633
C	5.40759187	5.46365238	-5.11277650
H	6.01422506	6.99038251	-3.73758415
H	6.18271669	5.69172434	-5.83958054
H	2.63386834	4.65222360	-2.51011801
H	4.22494366	6.44970148	-2.08464981
H	-8.35129558	1.12872546	3.50191221
H	-10.04813794	-0.09709139	4.80760757
C	-9.19658729	-4.12375377	2.70720576
C	-10.15766488	-4.75836956	3.46608510

C	-10.90568883	-4.04375059	4.43442312
C	-10.67346463	-2.69798315	4.62065706
H	-11.65831112	-4.55739416	5.02468840
H	-11.24171029	-2.13945880	5.35983246
H	-8.64357097	-4.70066944	1.97661381
H	-10.34246627	-5.81845086	3.31945345
H	0.37300185	-0.57160496	3.36018538

SY 7:

opt=loose b3lyp/6-311+g(2d,p) scrf=(solvent=ethanol,pcm) nosymm integral=grid=finegrid scf=(maxcycle=100,tight)

Title Card Required

1 1

C	9.18089311	7.15287501	9.09436628
C	9.31684536	5.86601844	8.28702113
C	9.30256932	6.17998010	6.78979232
C	10.49757193	7.06735938	6.44391323
C	10.77679601	8.15935278	7.46814283
C	10.04062770	8.20952302	8.68046198
C	11.79354183	9.09529667	7.18316602
C	12.54942592	9.13539455	6.01003900
C	8.20102258	7.13383109	10.10413119
C	7.67837109	8.11288716	10.96533292
C	6.62710053	7.89119549	11.85820671
C	5.89157257	6.57496662	12.19128061
C	4.92317510	7.03565558	13.26815483
C	5.07569231	8.41015486	13.46796205
C	3.97914529	6.32868494	14.00223813
C	3.19425664	7.01873228	14.93962615
C	3.35983087	8.39584834	15.12768135
C	4.30804725	9.11877643	14.39225584
C	5.09208057	6.04088575	10.97310331
C	15.25773783	11.04651655	4.54543024
C	15.18665900	11.78281859	5.73082366
C	14.09999056	11.20117759	6.61884711
C	13.58333200	10.04355219	5.74397985
C	14.71117345	10.69054779	7.94893831
C	16.16131682	11.35188701	3.52863131
C	17.01877870	12.43919682	3.73954600
C	16.96455692	13.18492191	4.92313150
C	16.04274920	12.85876304	5.93000835
C	6.84767373	5.49498603	12.75441283
C	12.99859755	12.26003338	6.87906283
C	14.11427501	9.05793565	3.48659114
C	13.07179340	9.52715161	2.46796664
C	6.46524357	10.30980593	12.58068357
C	7.48869100	10.67125621	13.66058204
C	11.06544686	9.20751961	11.13935207
C	11.10184055	10.13660738	12.19469629
C	11.79731233	9.84305678	13.36080540
C	12.47627992	8.62111772	13.50479019
C	12.43407195	7.69841918	12.44695821
C	11.74030745	7.98597733	11.27310072
C	13.18878433	8.37081508	14.78957154
S	10.20082992	9.71701967	9.66299528
N	6.08382108	8.89291617	12.60779496

N	14.29813040	10.00953351	4.59146459
H	10.26143542	5.36101978	8.53837694
H	8.50989582	5.17490439	8.54457641
H	8.36381893	6.68850647	6.53795459
H	9.34042555	5.25964282	6.19754476
H	10.34435078	7.52986734	5.46143338
H	11.39837338	6.44494195	6.34906123
H	12.00359821	9.82341689	7.95404540
H	12.32598257	8.41364346	5.23437059
H	7.73349247	6.15983898	10.18818390
H	8.08413733	9.11272042	10.92092437
H	3.84440408	5.26041724	13.85960746
H	2.45423434	6.47998148	15.52295744
H	2.74762103	8.91715561	15.85708541
H	4.43173338	10.18392133	14.55137510
H	5.74741495	5.70300539	10.16788742
H	4.47969086	5.19138452	11.28932397
H	4.42654395	6.81193960	10.57423585
H	13.95236133	10.25891315	8.60482980
H	15.17621442	11.52878885	8.47591005
H	15.47938128	9.93454724	7.76326578
H	16.20853948	10.78580721	2.60532643
H	17.73416480	12.70505933	2.96762941
H	17.63914107	14.02368071	5.06275244
H	16.00466188	13.44275152	6.84484146
H	7.55616459	5.14419880	12.00105044
H	7.41385052	5.88054748	13.60702517
H	6.26258451	4.63502913	13.09359883
H	13.43954920	13.11840841	7.39390556
H	12.56275256	12.61129409	5.93949497
H	12.19629816	11.86567409	7.50584894
H	15.08888732	8.92790315	3.01219695
H	13.84562363	8.08972226	3.91288741
H	12.99415902	8.78951922	1.66381393
H	12.08659718	9.63702892	2.92987999
H	13.35614261	10.48675783	2.02707080
H	6.84465794	10.54261053	11.58475278
H	5.54749226	10.89024172	12.70765685
H	7.72663777	11.73718123	13.59294414
H	7.09608000	10.47191747	14.66167802
H	8.41357943	10.10298436	13.53312308
H	10.58552977	11.08722213	12.10287536
H	11.81986761	10.55899454	14.17476807
H	11.72818098	7.25795755	10.47064390
H	12.92046283	6.72930212	12.51598763
H	14.20931736	6.92486127	13.98571055
O	14.07076479	7.34332786	14.84871572
O	13.01858140	9.04254749	15.79281286

SY 8:

opt=loose b3lyp/6-311+g(2d,p) scrf=(solvent=ethanol,pcm) nosymm integral=grid=finegrid scf=(maxcycle=100,tight)

Title Card Required

1 1

C	11.54079600	7.87866800	10.57670500
C	11.72689400	6.60921200	9.75136800
C	11.78405200	6.95199800	8.26151000
C	12.98913300	7.85282000	7.99330000
C	13.20790500	8.92635500	9.05058000
C	12.40719200	8.95081300	10.22237900
C	14.23767200	9.86805400	8.84020400
C	15.05681900	9.92839700	7.71104000
C	10.52256300	7.82446700	11.54753100
C	9.95766000	8.77704700	12.41078200
C	8.88368900	8.53330100	13.27208900
C	8.15243900	7.20907900	13.57816000
C	7.14563900	7.65067400	14.63821500
C	7.29048200	9.01064500	14.84437000
C	6.16102400	6.92293000	15.36169000
C	5.35031900	7.65752800	16.30310400
C	5.55001800	9.05653400	16.47439600
C	6.50536200	9.74499700	15.76055000
C	7.40867300	6.69732100	12.31386900
C	17.85477300	11.82560400	6.41688100
C	17.75703300	12.54009200	7.59708200
C	16.61576200	11.95740600	8.42634800
C	16.11490900	10.82647300	7.50997800
C	17.14109900	11.37113500	9.76397200
C	18.80413500	12.10938500	5.41122900
C	19.67598300	13.15087900	5.63779800
C	19.63083200	13.91899600	6.83579600
C	18.65004900	13.61755100	7.85048300
C	9.14096200	6.15862800	14.14716500
C	15.49680800	13.00977300	8.64627700
C	16.73717300	9.87494500	5.26186700
C	15.77608700	10.39129000	4.18767000
C	8.67535200	10.94149900	14.00789100
C	9.67445000	11.30875500	15.10821400
C	13.32985000	9.94550500	12.72715300
C	13.35390200	10.88536200	13.77377800
C	14.02392100	10.59828900	14.95614600
C	14.69034600	9.37253700	15.12573800
C	14.65841000	8.43836500	14.07788300
C	13.98892800	8.71871600	12.88823700
C	15.37978800	9.13061400	16.42431600
C	18.64455700	14.41713500	9.03187500
C	19.54688000	15.44662100	9.20043800

C	20.50797800	15.73913800	8.20139600
C	20.54430400	14.98826800	7.04619400
C	5.91760800	5.52371000	15.22302300
C	4.94238500	4.89145800	15.96571000
C	4.15166500	5.61638300	16.89144300
C	4.35576300	6.97002400	17.05178700
S	12.49785900	10.44831300	11.23005400
N	8.31155900	9.51900000	14.01684900
N	16.88044200	10.80382800	6.39238900
H	12.66293500	6.10721700	10.03865300
H	10.91497000	5.90620000	9.95618500
H	10.85561500	7.46008500	7.97312500
H	11.85749300	6.04360000	7.65436600
H	12.88249000	8.33321300	7.01318000
H	13.89750900	7.23714300	7.93207900
H	14.40276700	10.57949900	9.63708900
H	14.86671500	9.22417900	6.91083300
H	10.06700600	6.84234100	11.59020500
H	10.35321600	9.78169900	12.39658100
H	8.10388900	6.40125600	11.52662200
H	6.79411500	5.82988500	12.56262200
H	6.75081500	7.47458300	11.91436000
H	16.33713500	10.90894800	10.33949500
H	17.58274400	12.15973400	10.37623800
H	17.90929100	10.61498400	9.57840100
H	18.85096800	11.54133900	4.48968400
H	20.41981600	13.40223300	4.88739800
H	9.89790300	5.88129500	13.41134200
H	9.65184300	6.55099700	15.03110100
H	8.60902600	5.25123600	14.43931900
H	15.88246100	13.85904700	9.21330200
H	15.12507900	13.38327600	7.68793500
H	14.65534000	12.59041800	9.20053100
H	17.73447900	9.71667500	4.84746600
H	16.41125000	8.91073900	5.65495800
H	15.71713800	9.66151300	3.37480300
H	14.77067900	10.53746300	4.59241600
H	16.12292900	11.34109400	3.77084400
H	9.07065200	11.18410500	13.02060800
H	7.74935100	11.51137800	14.11860300
H	9.90134800	12.37762000	15.04981200
H	9.26655200	11.10010700	16.10132000
H	10.60821000	10.75197800	14.99508400
H	12.84960200	11.84027000	13.66167500
H	14.03780800	11.32315800	15.76238700
H	13.98254300	7.98046100	12.09525900
H	15.13302700	7.46510500	14.16773100
H	16.41186800	7.67854600	15.64730100
H	21.21155800	16.55265600	8.34898500
H	21.27721100	15.20395400	6.27338300

H	17.92413800	14.22045500	9.81584900
H	19.52000300	16.03934200	10.10997100
H	6.64498200	10.80913500	15.90930400
H	4.92865700	9.58565200	17.19102700
H	3.38850100	5.10442700	17.46950100
H	3.75427500	7.53665500	17.75770000
H	6.50328200	4.93857100	14.52518900
H	4.77942200	3.82523200	15.83916000
O	16.25818900	8.10139200	16.50565600
O	15.19457900	9.81035400	17.41952400

1.3 Energies of the first five triplet states (T_1-T_5, in eV) at the S1-optimised geometry for SY 1 - SY 6.

Table S9. The first five triplet state energies (T_1-T_5, in eV) at the optimised first excited state (S_1) (**SY 1 - SY 6**). To assess the energetic accessibility of triplet states involved in intersystem crossing, the first five triplet excited states (T_1-T_5) were calculated at the optimised geometry of the first singlet excited state (S_1). The S_1 geometry was obtained by excited-state optimisation, and single-point triplet-state calculations were subsequently performed at this geometry using the same level of theory.

	**SY 1**	**SY 2**	**SY 3**	**SY 4**	**SY 5**	**SY 6**
T_1	0.8804	0.8424	0.8508	0.7659	0.7948	0.8300
T_2	2.0105	1.7498	1.6207	1.7798	1.6582	1.5763
T_3	2.5545	2.2559	2.1856	2.2267	2.1433	2.0817
T_4	2.6213	2.4979	2.4831	2.2505	2.2630	2.2651
T_5	2.8044	2.6838	2.6673	2.6074	2.3316	2.3080

2. Singlet oxygen evaluation of SY 1 - SY 8

Quantifying singlet oxygen generation of NIR cyanine dyes

Singlet oxygen generation upon light irradiation was quantified using 1,3-diphenylisobenzofuran (DPBF) as a singlet oxygen sensor. NIR cyanine dyes (**SY 1 - SY 8**, 10 µM) were mixed with DPBF (200 µM) in methanol. Absorbance was first measured in the dark, followed by irradiation of the mixture and subsequent absorbance measurements at various time intervals (0 - 10 minutes). A decrease in DPBF absorbance over time upon light exposure indicates the production of singlet oxygen, forming the colourless product 1,2-dibenzoylbenzene, as illustrated in Figure S1. The experiments were carried out using a white light source equipped with a 495 nm long-pass glass filter (0.02 W/cm^2).

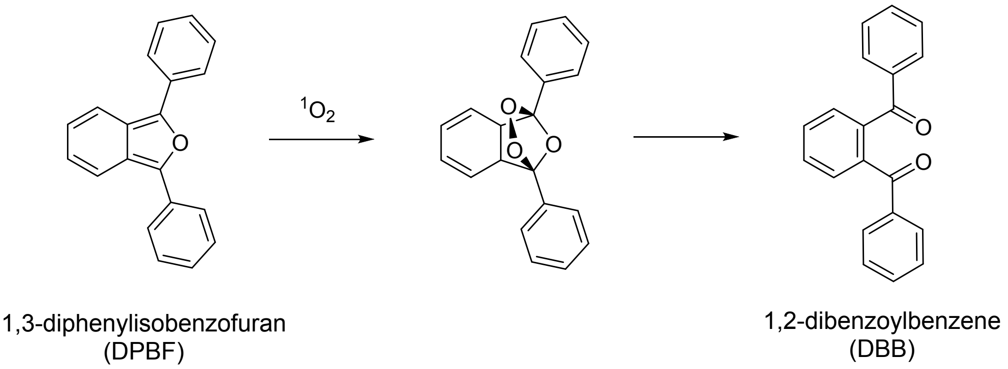

Figure S1: The proposed scheme of the reaction from DPBF to DBB.

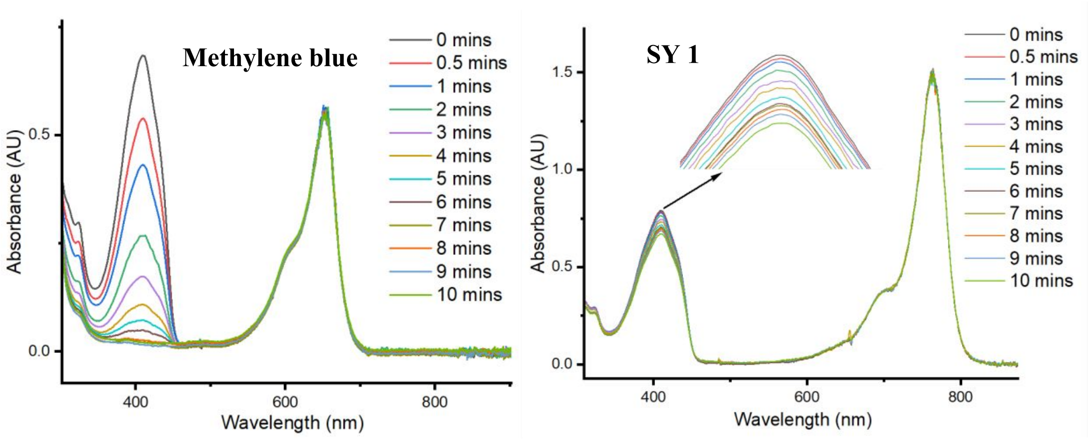

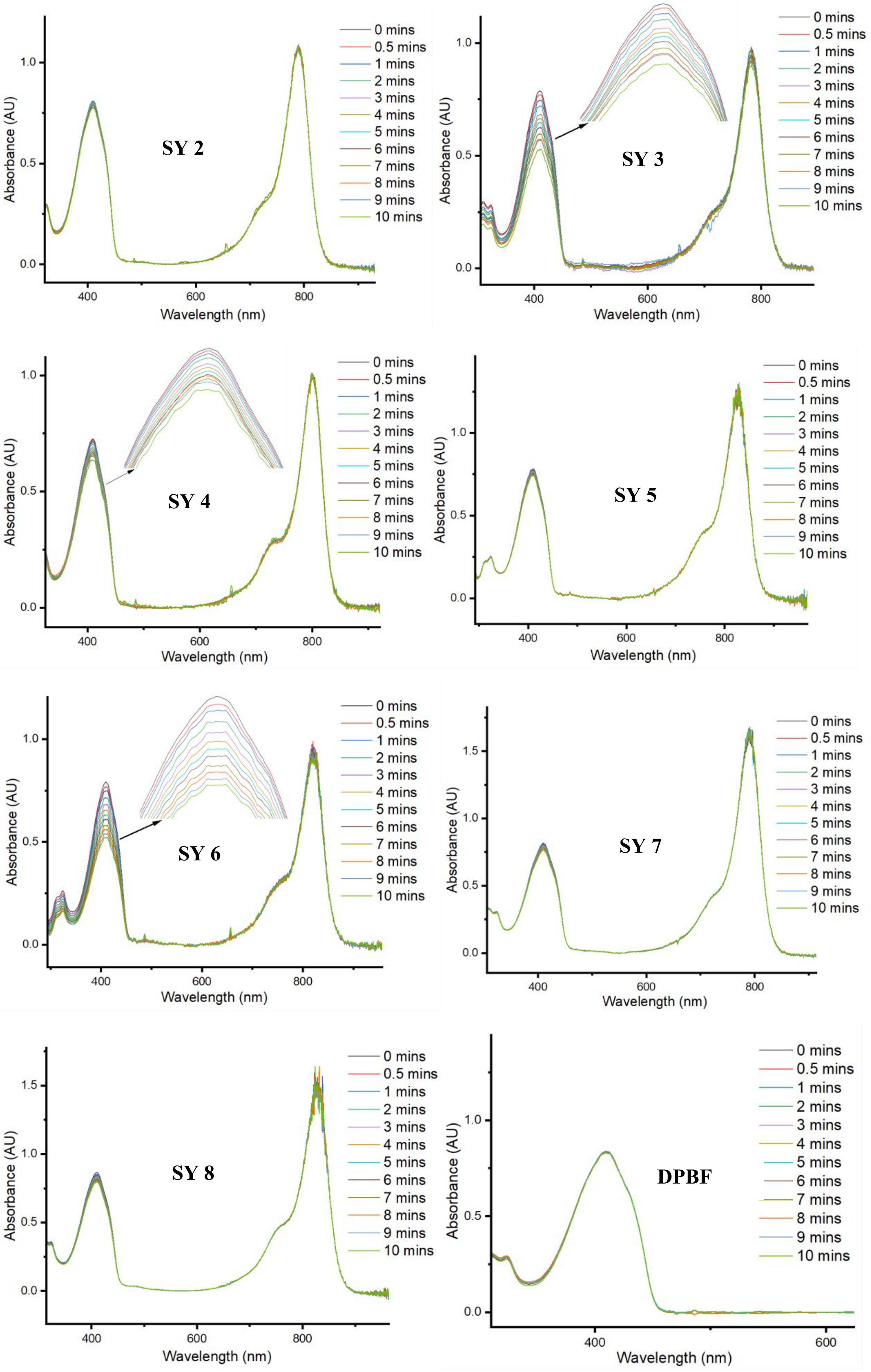

Figure S2: Change of absorbance with time for **SY 1** - **SY 8** (5 µM) and Methylene blue (MB) (5 µM) and DPBF (50µM) in MeOH upon irradiation (0.02 W/cm^2, > 490 nm).

2.1. Singlet oxygen evaluation of SY 1 – SY 8 over time.

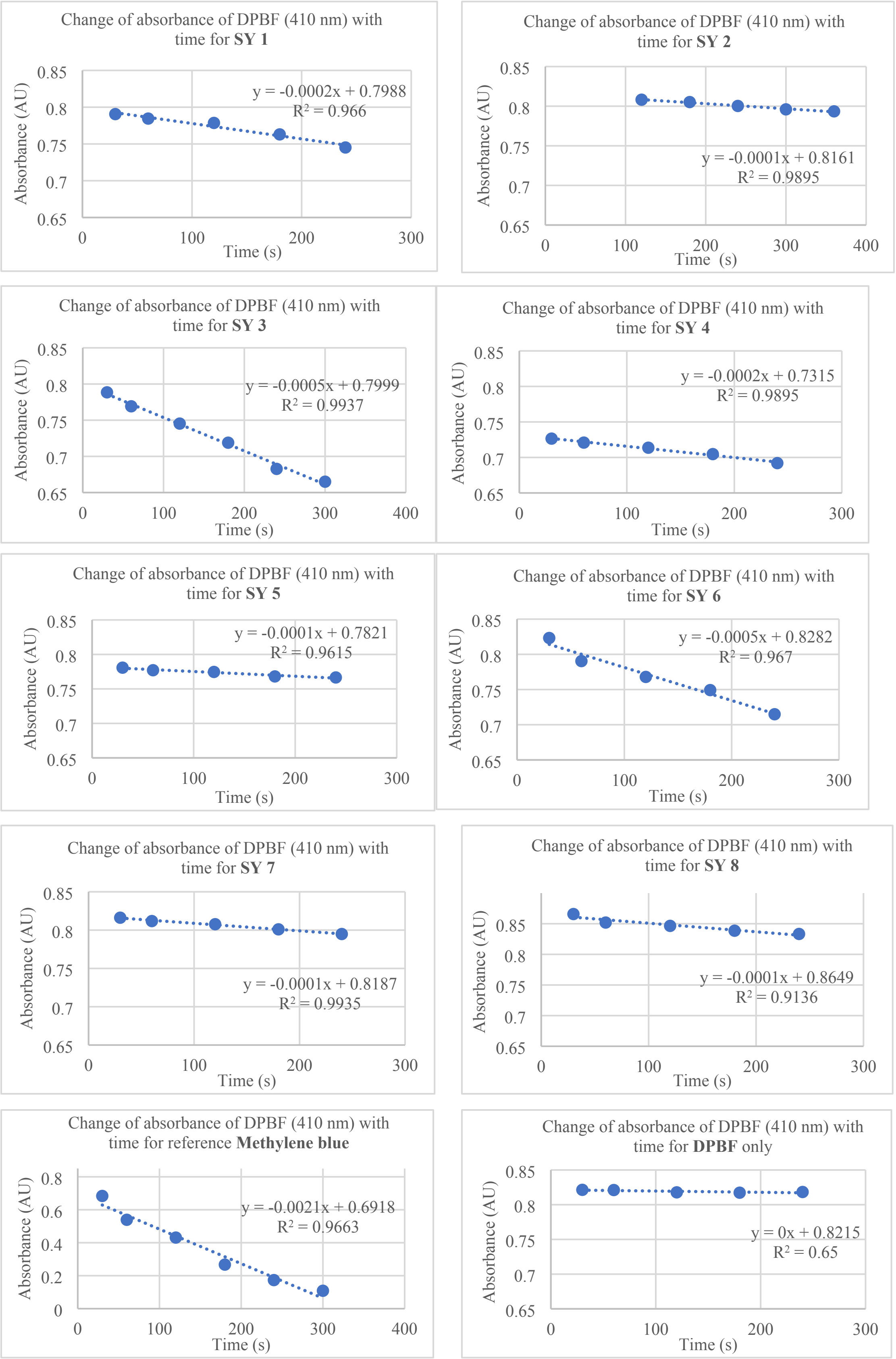

Figure S3: Graphs of the change of λ_{max} (absorption at 410 nm) of DPBF over time for **SY 1** - **SY 8** (5 µM) and the reference Methylene Blue (5 µM), and negative control DPBF only (50 µM) in methanol with the irradiation of light (0.02 W/cm^2, > 490 nm)

3. Oxidation test of SY 5 (S) and SY 6 (Se) with H_2O_2 (5 %)

The oxidation stability of **SY 5** (S) and **SY 6** (Se) was evaluated using hydrogen peroxide (H_2O_2, 5 %) as the oxidising agent. The compounds were dissolved in a mixed solvent of ethanol and water (1:1, v/v) containing 5 % H_2O_2. The solutions were monitored over time, with measurements taken at 0, 30, and 60 minutes.

As shown in Figure S4, **SY 5** (S) displayed no significant spectral or visual changes throughout the 60-minute period, indicating good stability under oxidative conditions. Similarly, Figure S5 demonstrates that **SY 6** (Se) also remained unchanged under the same treatment, suggesting comparable resistance toward oxidation.

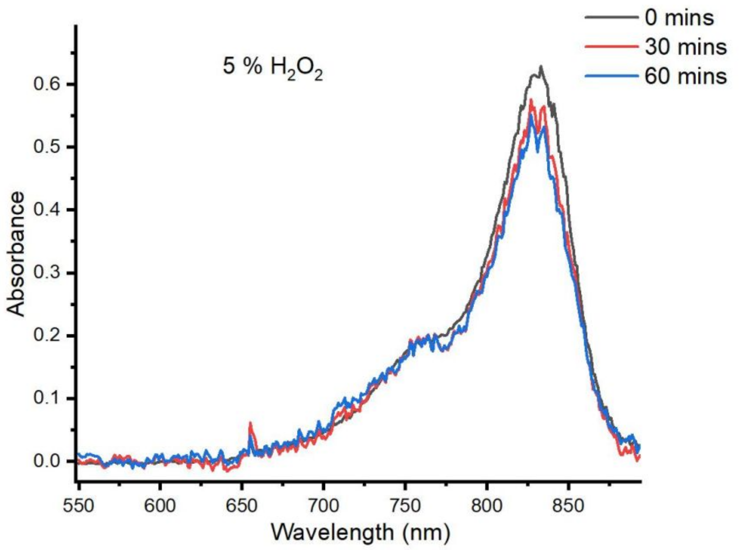

Figure S4. Spectra of **SY 5** (S) in EtOH/H_2O (1:1, v/v) under 5 % H_2O_2 at 0, 30, and 60 min respectively.

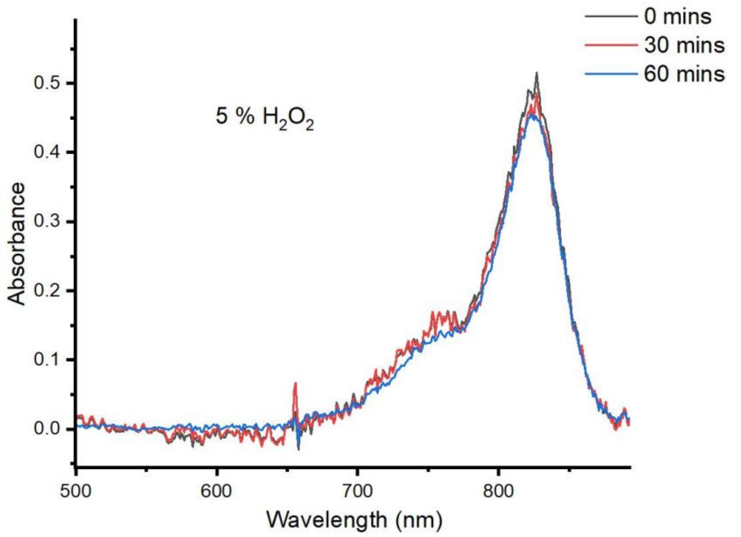

Figure S5. Spectra of **SY 6** (Se) in EtOH/H_2O (1:1, v/v) under 5 % H_2O_2 at 0, 30, and 60 min respectively.

4. Synthetic procedures:

Synthesis of 1-ethyl-2,3,3-trimethyl-3H-indol-1-ium (10)[9]

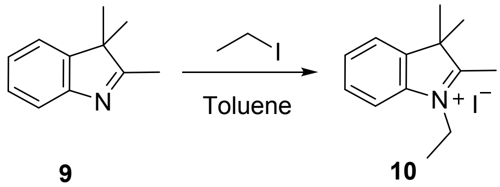

2, 3, 3-trimethyl-3H-indolenine (4.96 g, 31.2 mmol) and iodoethane (6 g, 38.4 mmol) were mixed in 15 mL dry toluene and the mixture was refluxed under N_2 atmosphere. The reaction was monitored by TLC and stopped when the starting materials were consumed. After cooling to room temperature, the precipitate was collected by filtered and washed with diethyl ether, to give a pink solid of **10** (8 g, yield: 82 %).

Synthesis of 2-((E)-2-((E)-2-chloro-3-(2-((E)-1-ethyl-3,3-dimethylindolin-2-ylidene)ethylidene)cyclohex-1-en-1-yl)vinyl)-1-ethyl-3,3-dimethyl-3H-indol-1-ium (12) [10]

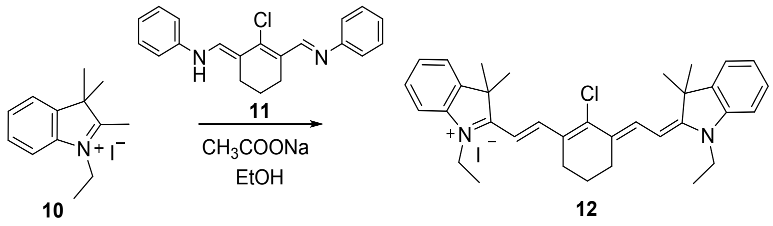

Compound **11** (500 mg, 1.6 mmol), Compound **10** (1222 mg, 3.9 mmol), and anhydrous sodium acetate (260 mg, 3.2 mmol) dissolved in 100 ml of ethanol and heating under reflux for overnight. After compound **11** had disappeared (analysis via TLC), the reaction was stopped and cooled down below 0 °C. The solid was collected by filtration and washed with diethyl ether (500 mL). The crude product was purified by column chromatography on silica gel (eluting with dichloromethane and ethanol (50:1 to 10:1), to yield the title compound as a dark blue solid (390 mg, 39 %).

Synthesis of 3-ethyl-1,1,2-trimethyl-1H-benzo[e]indol-3-ium (14)[11]

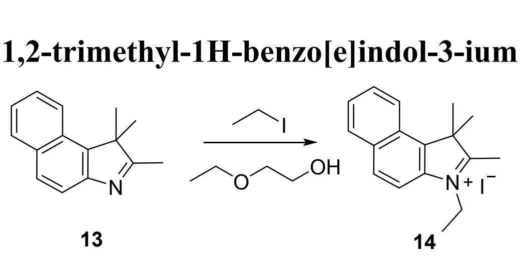

1,1,2-Trimethyl-1H-benz[e]indole (**13**) (2.6 g, 12.5 mmol) was dissolved in 2-ethoxyethanol (10 mL). Iodoethane (2.91 g, 18.7 mmol) was added, and the mixture was heated at 120 °C for 24 h under an N_2 atmosphere. After the consumption of **13**, the reaction was cooled to room temperature. The precipitate was collected by filtration

and washed with diethyl ether to afford the title compound as a pale blue solid (3.8 g, 84 % yield).

Synthesis of 2-((E)-2-((E)-2-chloro-3-((E)-2-(3-ethyl-1,1-dimethyl-1,3-dihydro-2H-benzo[e]indol-2-ylidene)ethylidene)cyclohex-1-en-1-yl)vinyl)-3-ethyl-1,1-dimethyl-1H-benzo[e]indol-3-ium (15)[12]

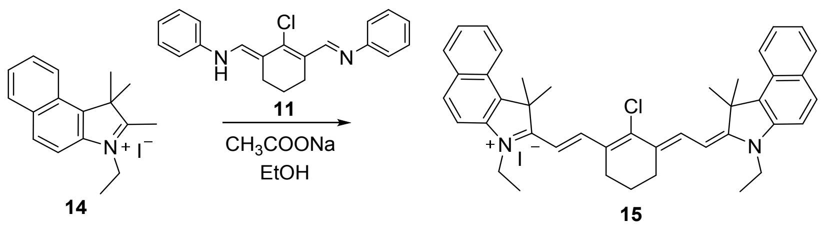

Compound **11** (500 mg, 1.6 mmol), Compound **14** (1699 mg, 4.7 mmol), and anhydrous sodium acetate (381 mg, 4.7 mmol) dissolved in ethanol (100 ml) and heated under reflux for 24 h. After the consumption of **11** (analysis via TLC), the reaction was stopped and cooled down below 5 °C. The solid residue was obtained through filtration and washed with diethyl ether (500 mL). And then the solid was extracted with dichloromethane (15 mL) and the crude was purified by column chromatography on silica gel (eluting using dichloromethane: ethanol (100:1 to 10:1) to yield a green solid product (460 mg, 40 %).

5. Proton NMR and Carbon NMR of SY 1 to SY 8

Proton NMR of 1-ethyl-2-((E)-2-((E)-3-(2-((E)-1-ethyl-3,3-dimethylindolin-2-ylidene)ethylidene)-2-phenoxycyclohex-1-en-1-yl)vinyl)-3,3-dimethyl-3H-indol-1-ium (SY 1)

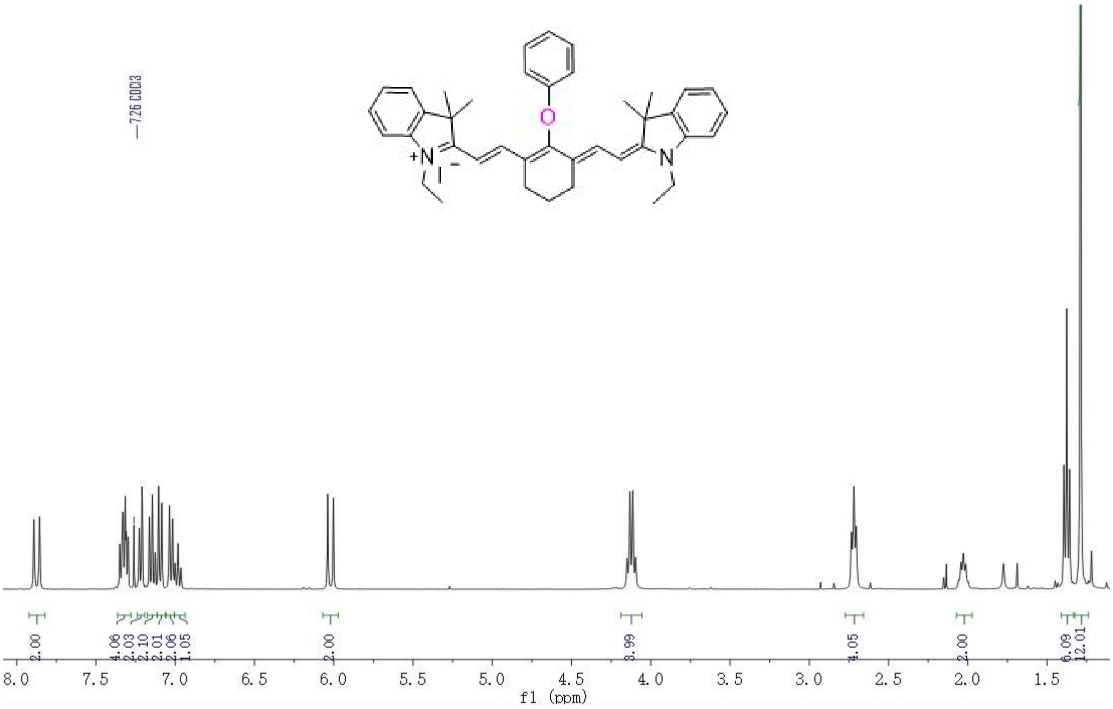

Carbon NMR of 1-ethyl-2-((E)-2-((E)-3-(2-((E)-1-ethyl-3,3-dimethylindolin-2-ylidene)ethylidene)-2-phenoxycyclohex-1-en-1-yl)vinyl)-3,3-dimethyl-3H-indol-1-ium (SY 1)

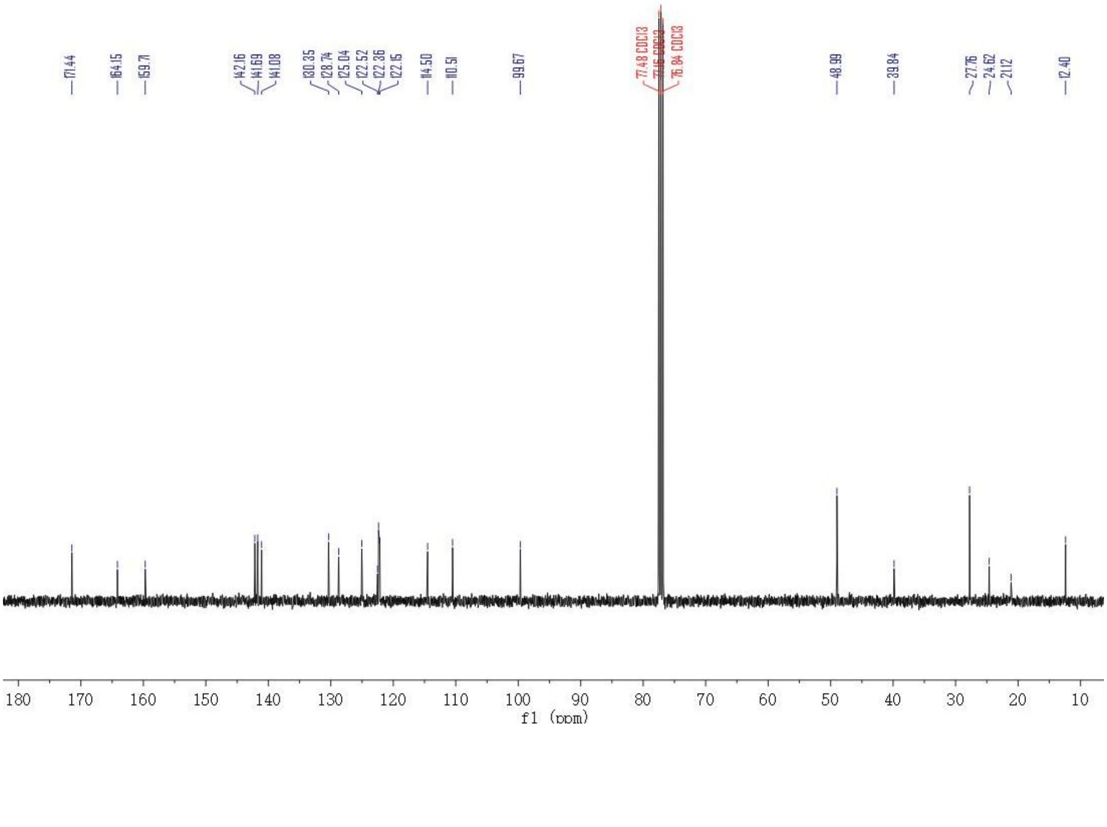

Proton NMR of 1-ethyl-2-((E)-2-((E)-3-(2-((E)-1-ethyl-3,3-dimethylindolin-2-ylidene)ethylidene)-2-(phenylthio)cyclohex-1-en-1-yl)vinyl)-3,3-dimethyl-3H-indol-1-ium (SY 2)

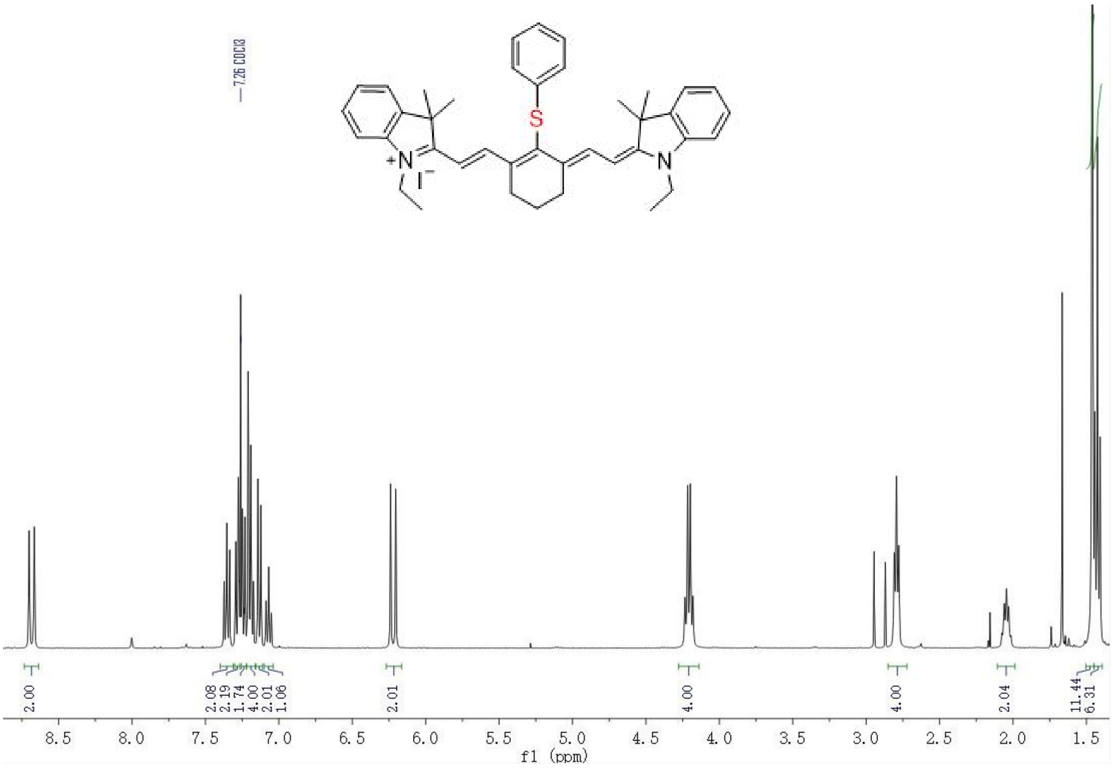

Carbon NMR of 1-ethyl-2-((E)-2-((E)-3-(2-((E)-1-ethyl-3,3-dimethylindolin-2-ylidene)ethylidene)-2-(phenylthio)cyclohex-1-en-1-yl)vinyl)-3,3-dimethyl-3H-indol-1-ium (SY 2)

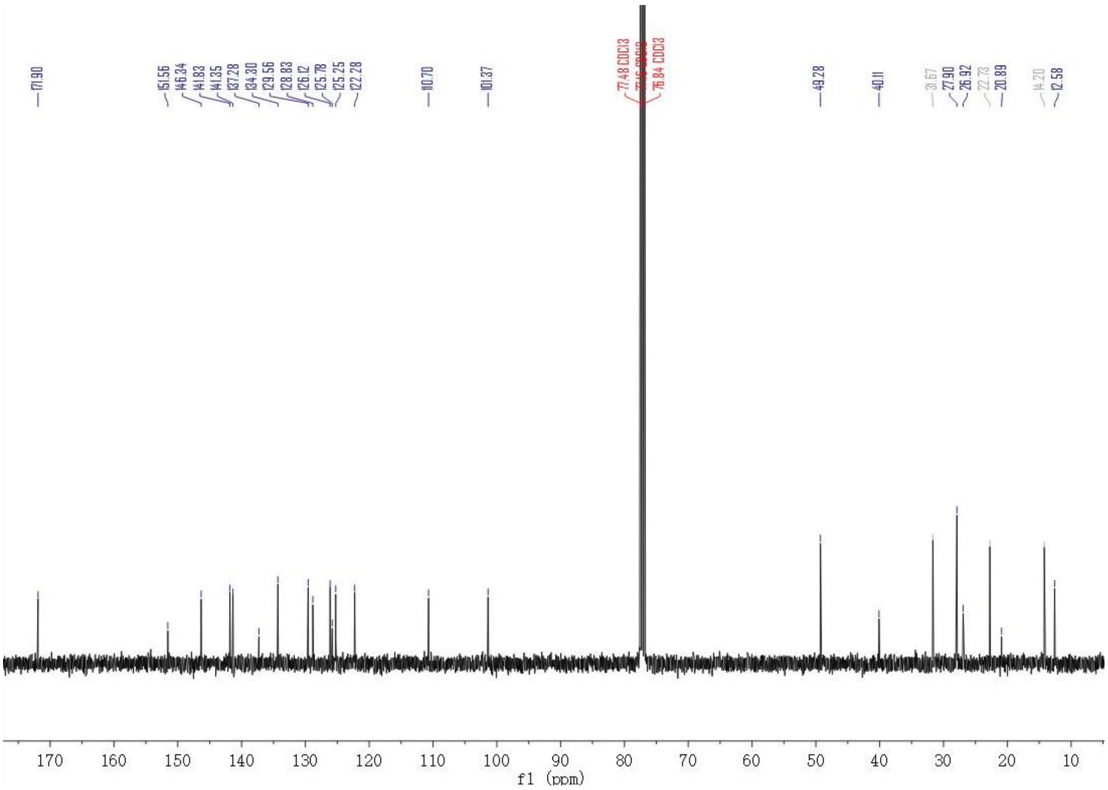

Proton NMR of 1-ethyl-2-((E)-2-((E)-3-(2-((E)-1-ethyl-3,3-dimethylindolin-2-ylidene)ethylidene)-2-(phenylselanyl)cyclohex-1-en-1-yl)vinyl)-3,3-dimethyl-3H-indol-1-ium (SY 3)

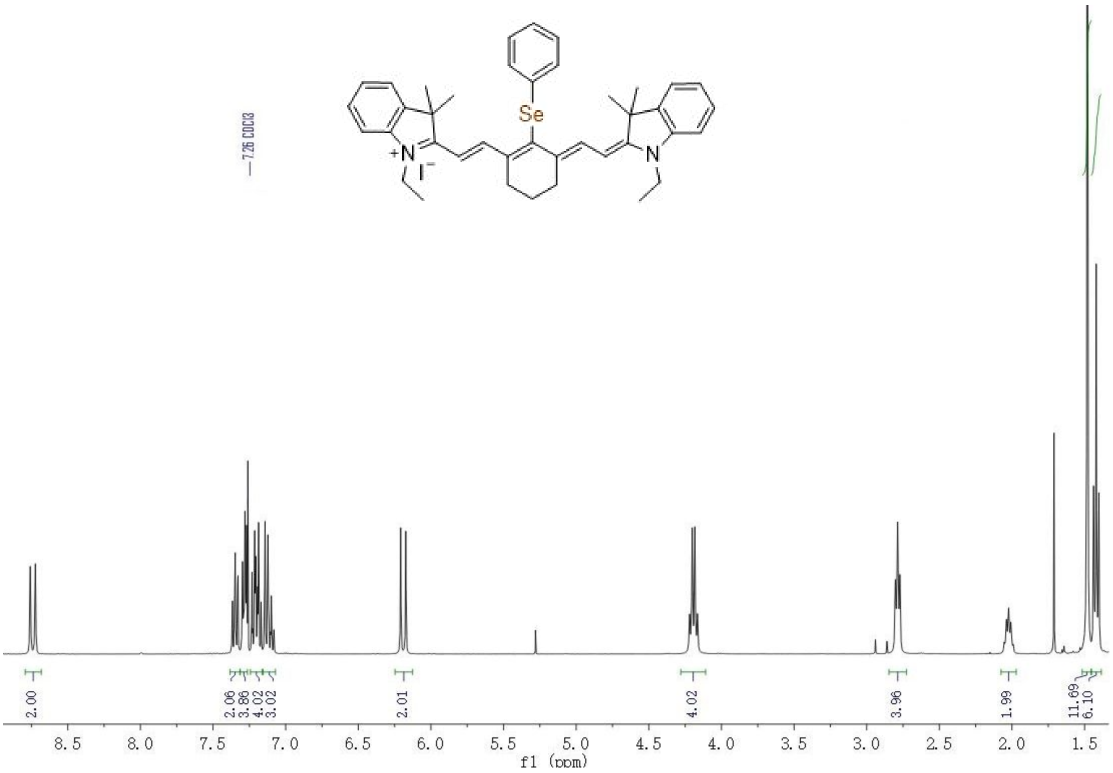

Carbon NMR of 1-ethyl-2-((E)-2-((E)-3-(2-((E)-1-ethyl-3,3-dimethylindolin-2-ylidene)ethylidene)-2-(phenylselanyl)cyclohex-1-en-1-yl)vinyl)-3,3-dimethyl-3H-indol-1-ium (SY 3)

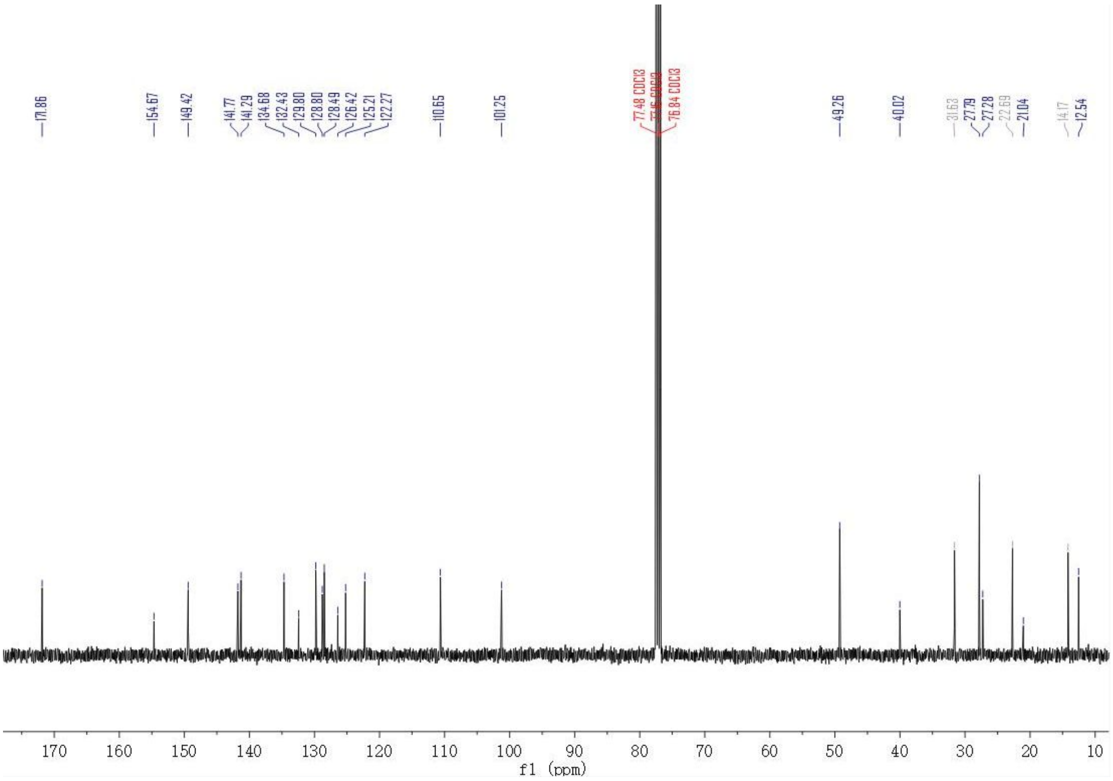

Proton NMR of 3-ethyl-2-((E)-2-((E)-3-((E)-2-(3-ethyl-1,1-dimethyl-1,3-dihydro-2H-benzo[e]indol-2-ylidene)ethylidene)-2-phenoxycyclohex-1-en-1-yl)vinyl)-1,1-dimethyl-1H-benzo[e]indol-3-ium (SY 4)

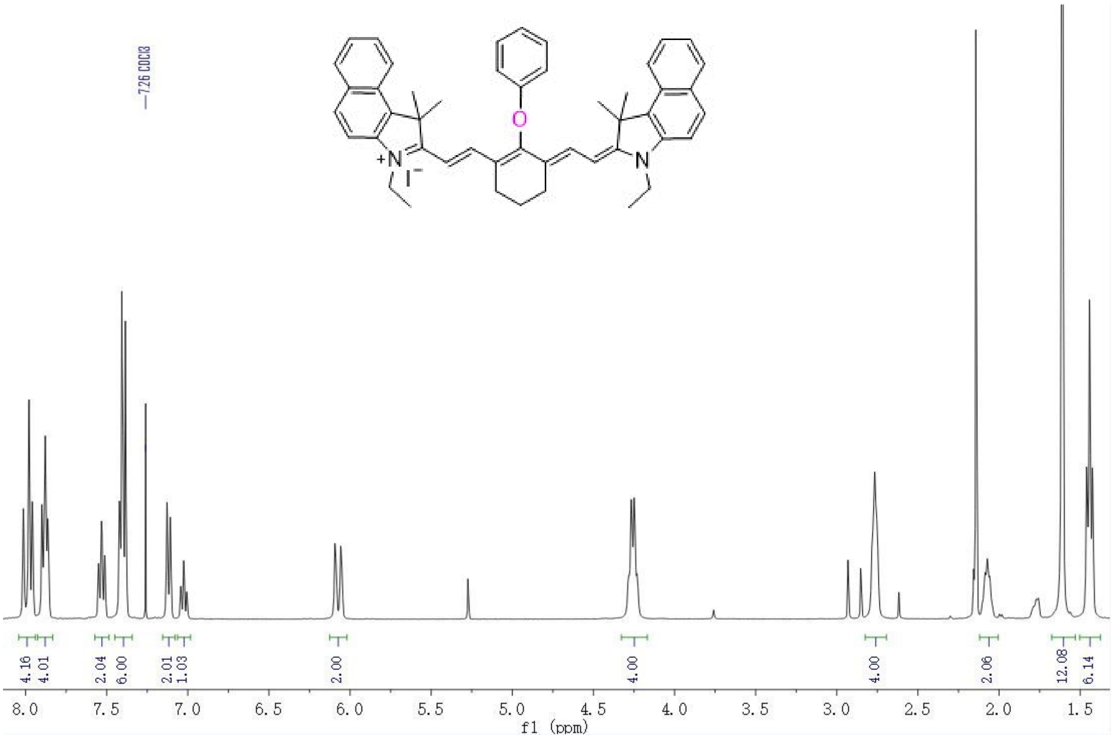

Carbon NMR of 3-ethyl-2-((E)-2-((E)-3-((E)-2-(3-ethyl-1,1-dimethyl-1,3-dihydro-2H-benzo[e]indol-2-ylidene)ethylidene)-2-phenoxycyclohex-1-en-1-yl)vinyl)-1,1-dimethyl-1H-benzo[e]indol-3-ium (SY 4)

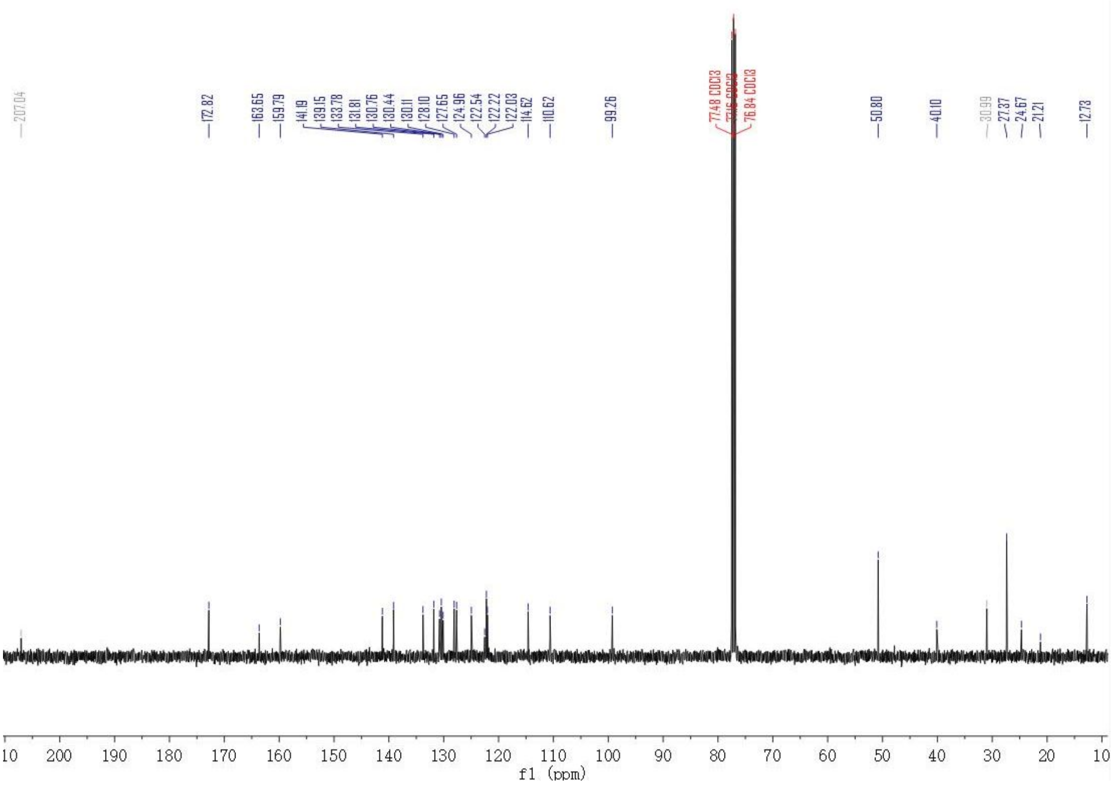

Proton NMR of 3-ethyl-2-((E)-2-((E)-3-((E)-2-(3-ethyl-1,1-dimethyl-1,3-dihydro-2H-benzo[e]indol-2-ylidene)ethylidene)-2-(phenylthio)cyclohex-1-en-1-yl)vinyl)-1,1-dimethyl-1H-benzo[e]indol-3-ium (SY 5)

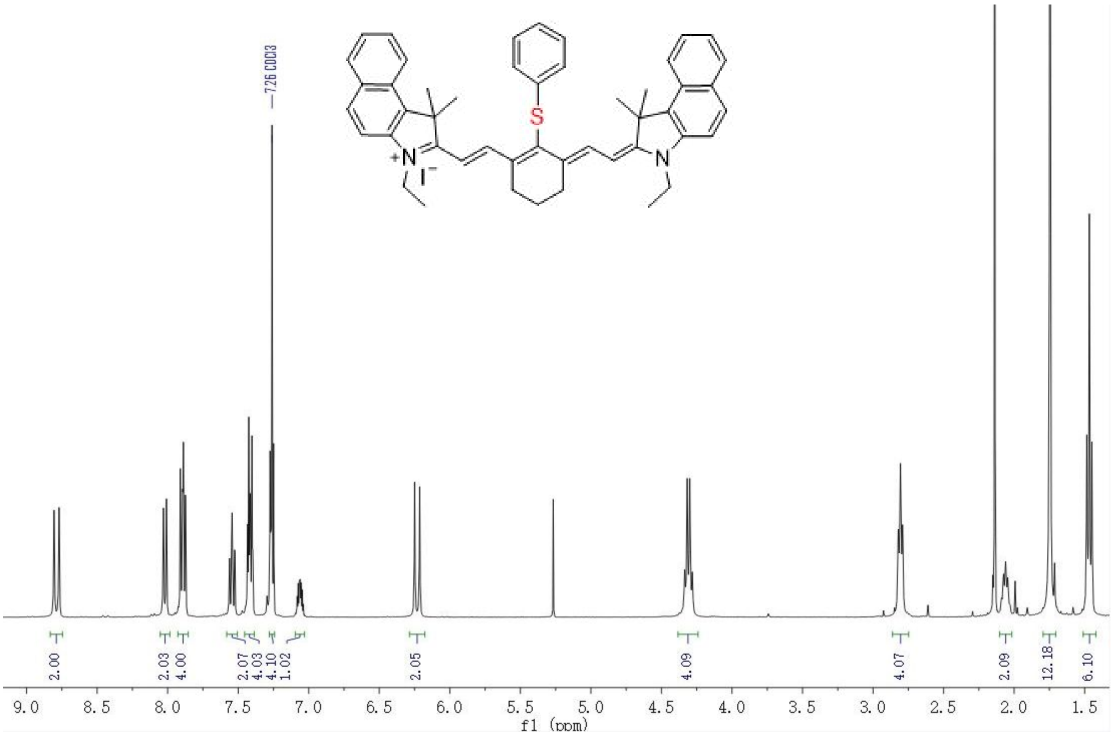

Carbon NMR of 3-ethyl-2-((E)-2-((E)-3-((E)-2-(3-ethyl-1,1-dimethyl-1,3-dihydro-2H-benzo[e]indol-2-ylidene)ethylidene)-2-(phenylthio)cyclohex-1-en-1-yl)vinyl)-1,1-dimethyl-1H-benzo[e]indol-3-ium (SY 5)

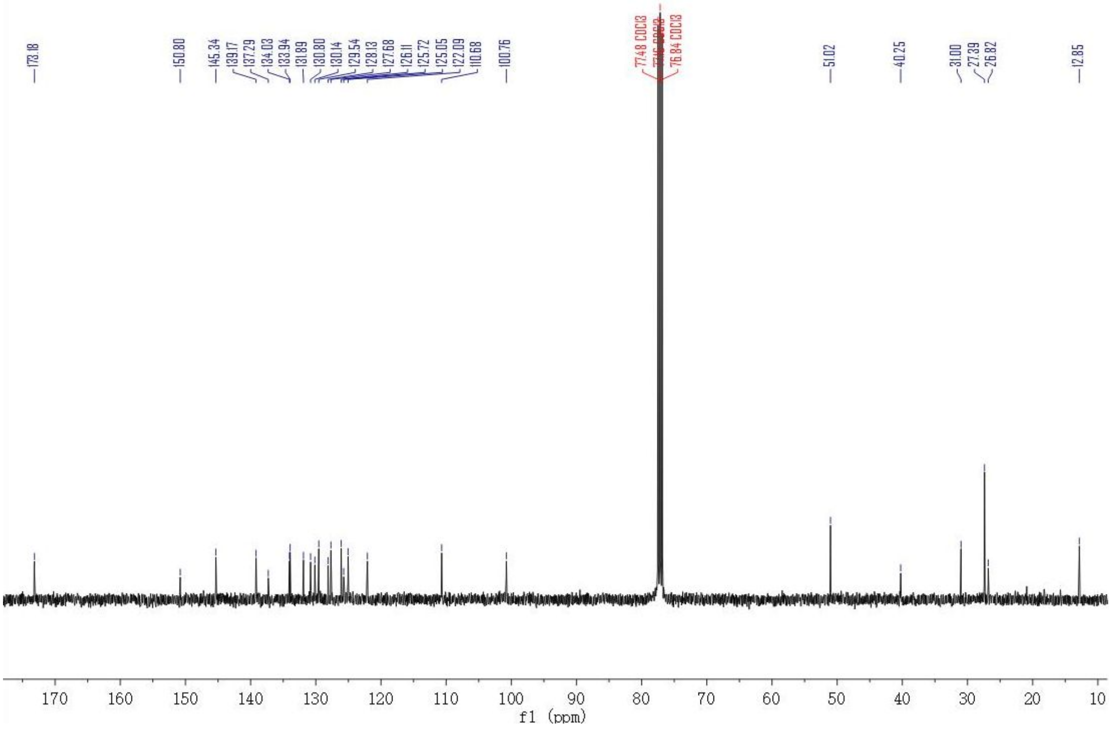

Proton NMR of 3-ethyl-2-((E)-2-((E)-3-((E)-2-(3-ethyl-1,1-dimethyl-1,3-dihydro-2H-benzo[e]indol-2-ylidene)ethylidene)-2-(phenylselanyl)cyclohex-1-en-1-yl)vinyl)-1,1-dimethyl-1H-benzo[e]indol-3-ium (SY 6)

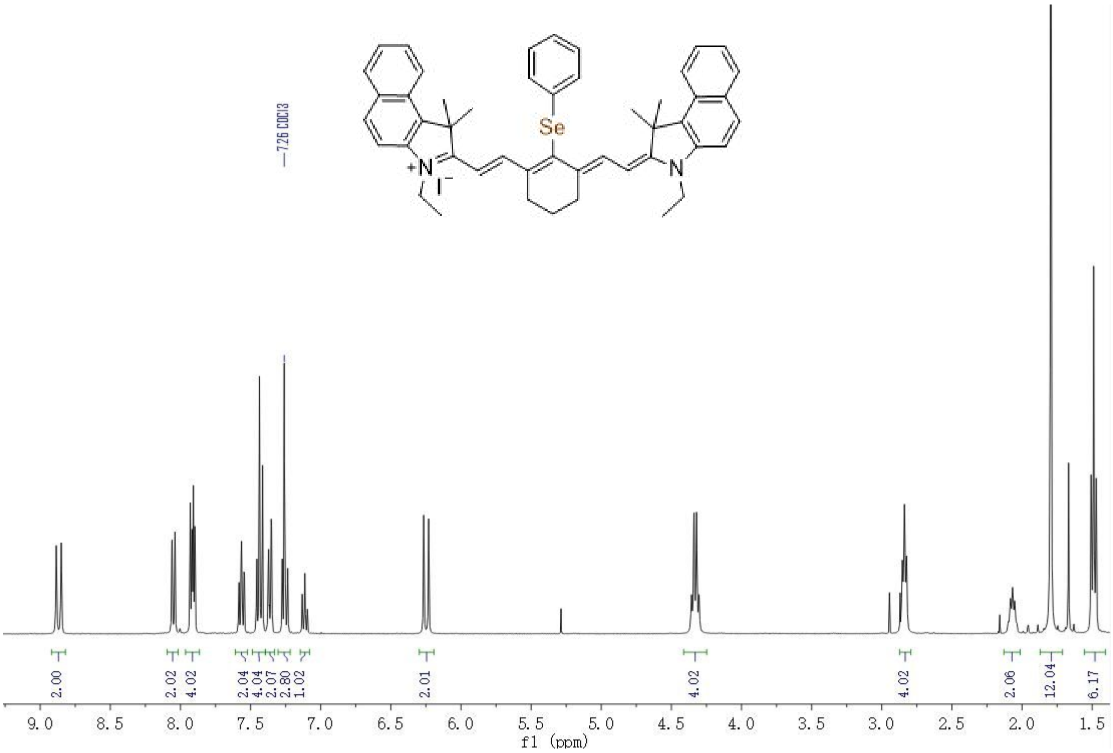

Carbon NMR of 3-ethyl-2-((E)-2-((E)-3-((E)-2-(3-ethyl-1,1-dimethyl-1,3-dihydro-2H-benzo[e]indol-2-ylidene)ethylidene)-2-(phenylselanyl)cyclohex-1-en-1-yl)vinyl)-1,1-dimethyl-1H-benzo[e]indol-3-ium (SY 6)

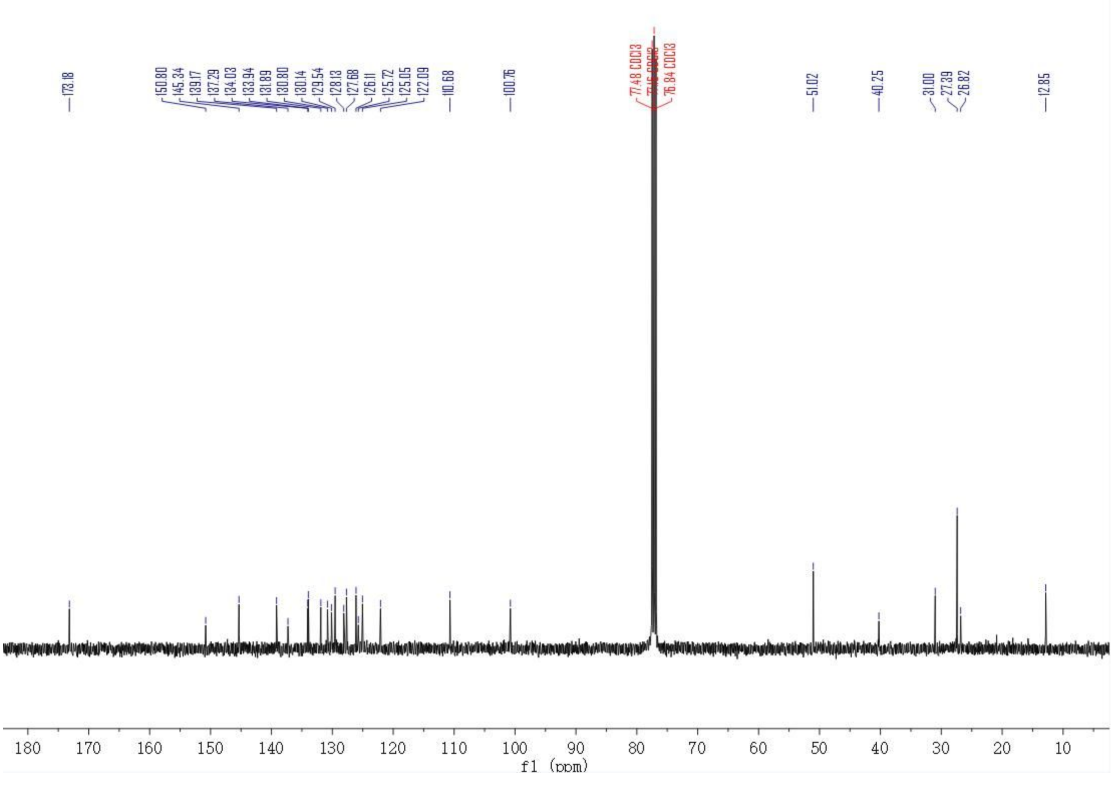

Proton NMR of 2-((E)-2-((E)-2-((4-carboxyphenyl)thio)-3-(2-((E)-1-ethyl-3,3-dimethylindolin-2-ylidene)ethylidene)cyclohex-1-en-1-yl)vinyl)-1-ethyl-3,3-dimethyl-3H-indol-1-ium (SY 7)

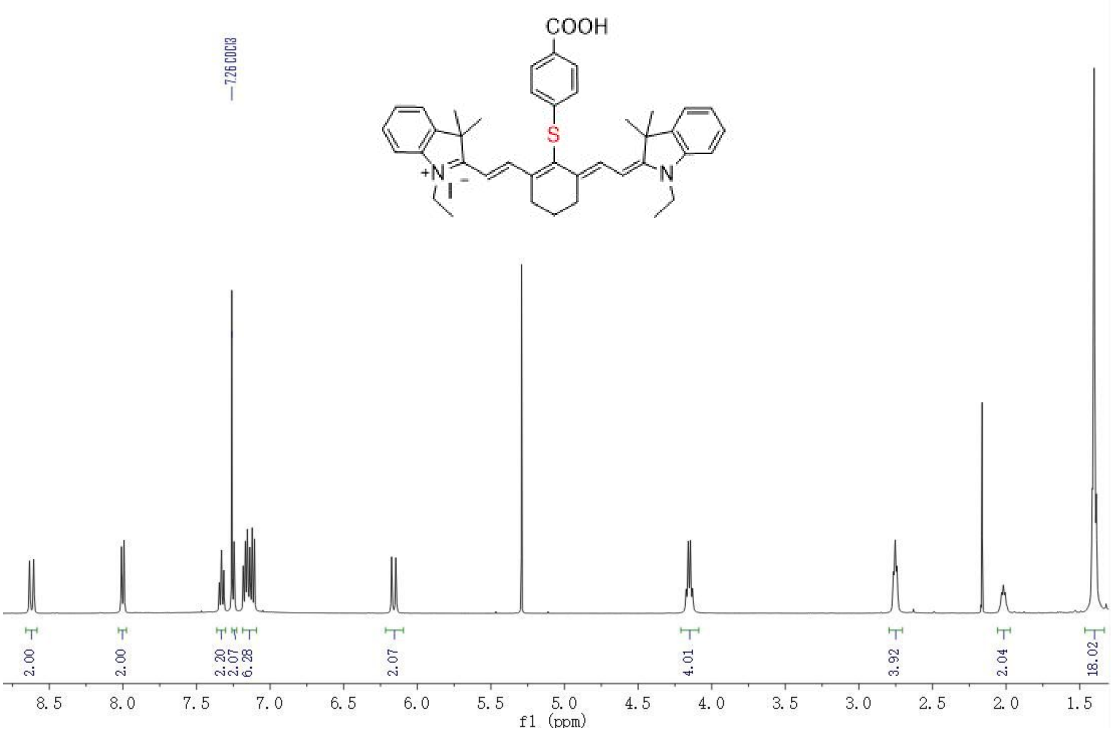

Carbon NMR of 2-((E)-2-((E)-2-((4-carboxyphenyl)thio)-3-(2-((E)-1-ethyl-3,3-dimethylindolin-2-ylidene)ethylidene)cyclohex-1-en-1-yl)vinyl)-1-ethyl-3,3-dimethyl-3H-indol-1-ium (SY 7)

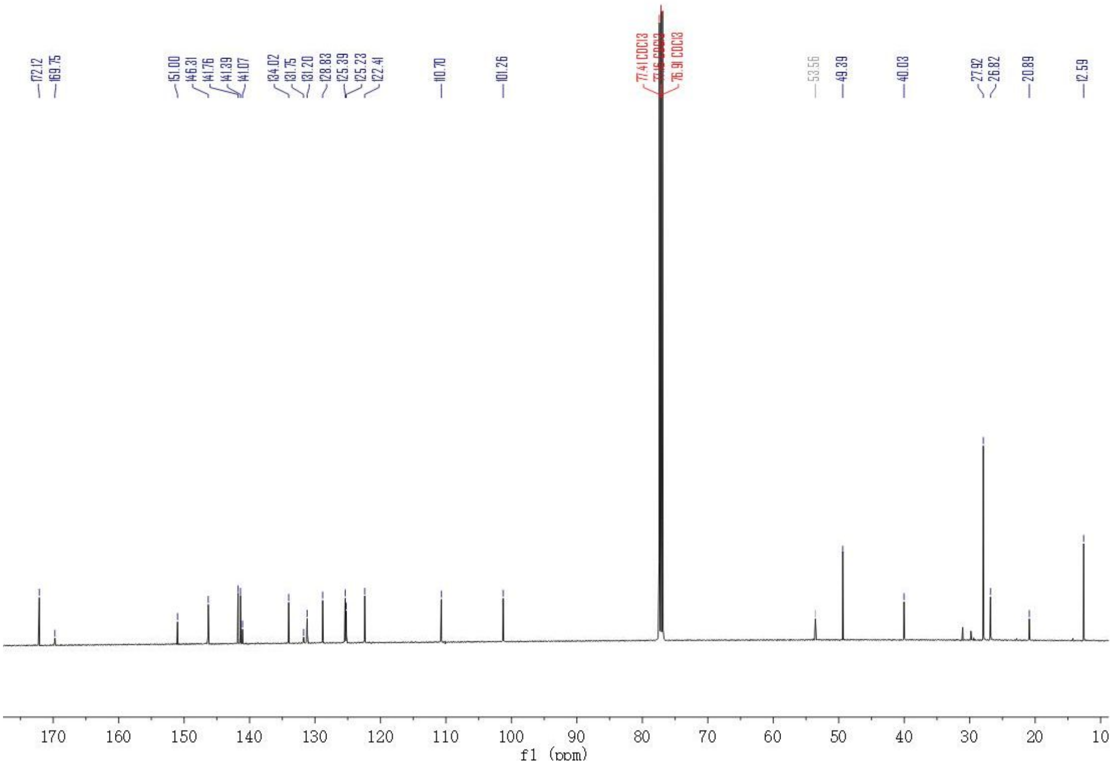

Proton NMR of 2-((E)-2-((E)-2-((4-carboxyphenyl)thio)-3-((E)-2-(3-ethyl-1,1-dimethyl-1,3-dihydro-2H-benzo[e]indol-2-ylidene)ethylidene)cyclohex-1-en-1-yl)vinyl)-3-ethyl-1,1-dimethyl-1H-benzo[e]indol-3-ium (SY 8)

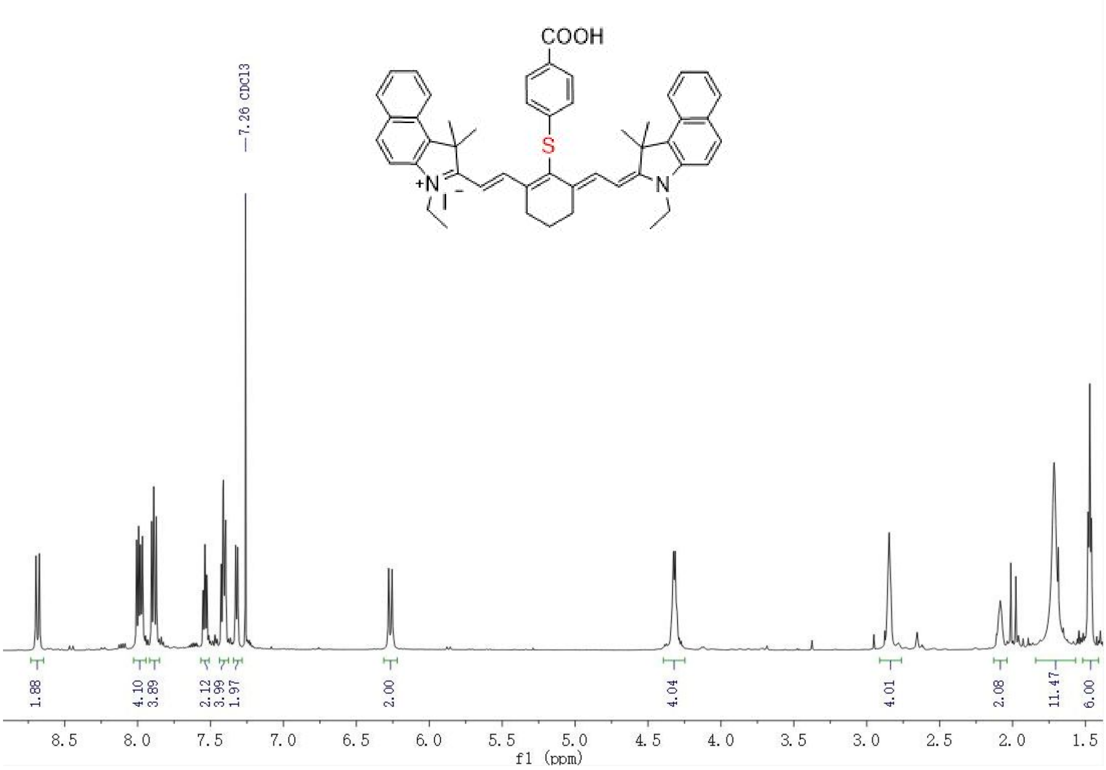

Carbon NMR of 2-((E)-2-((E)-2-((4-carboxyphenyl)thio)-3-((E)-2-(3-ethyl-1,1-dimethyl-1,3-dihydro-2H-benzo[e]indol-2-ylidene)ethylidene)cyclohex-1-en-1-yl)vinyl)-3-ethyl-1,1-dimethyl-1H-benzo[e]indol-3-ium (SY 8)

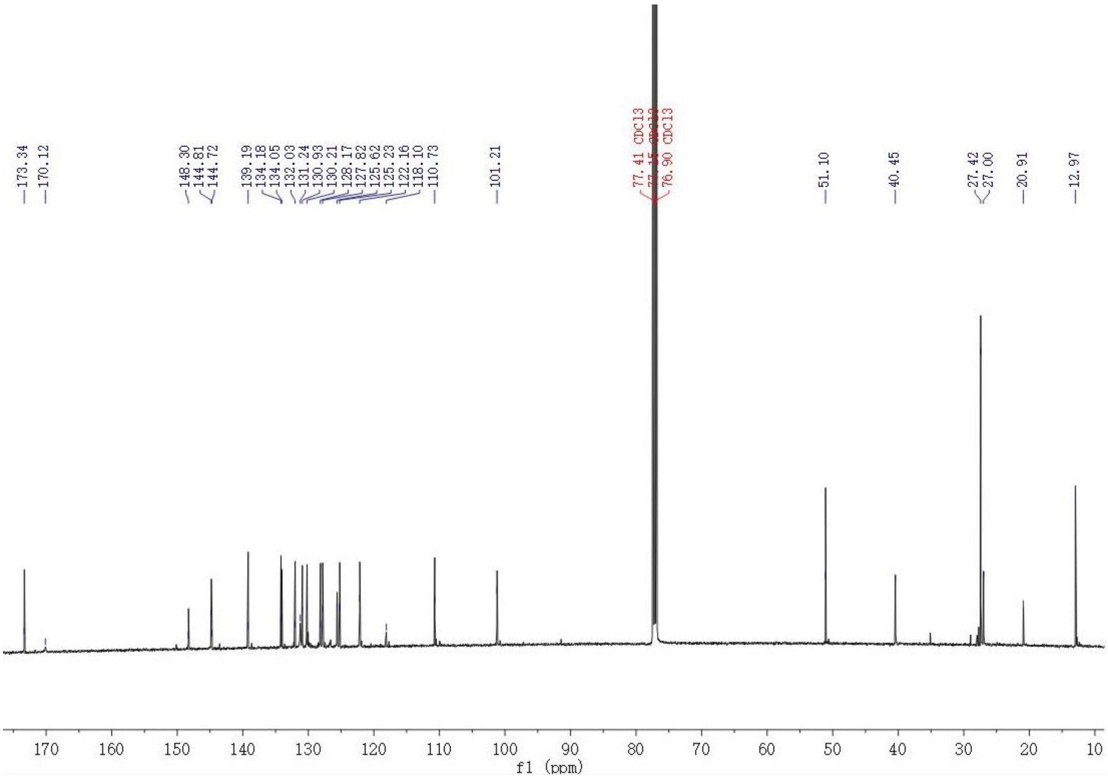

6. Mass spec of SY 1 to SY 8

Mass spec of SY 1

Display Report

Analysis Info

Analysis Name	D:\Data\Alans Data July 2024\LKF_Yang 19574 (310724) YSF590.d	Acquisition Date	7/31/2024 12:34:28 PM
Method	230724VGalanstune_low.m	Operator	demo
Sample Name	Yang 19574 (310724) YSF590	Instrument	micrOTOF 8213750.10408
Comment	Yang 19574 (310724) YSF590		

Acquisition Parameter

Source Type	ESI	Ion Polarity	Positive	Set Nebulizer	0.2 Bar
Focus	Not active			Set Dry Heater	200 °C
Scan Begin	50 m/z	Set Capillary	3500 V	Set Dry Gas	6.0 l/min
Scan End	3000 m/z	Set End Plate Offset	-500 V	Set Divert Valve	Waste

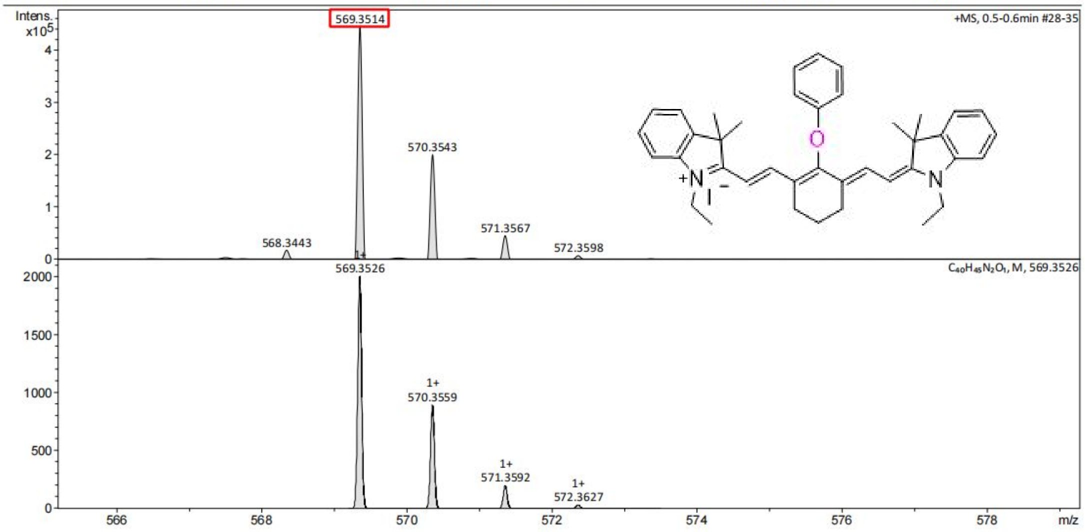

Bruker Compass DataAnalysis 4.1 printed: 7/31/2024 12:42:29 PM by: demo 1 of 1

Display Report

Analysis Info

Analysis Name	D:\Data\Alans Data Aug 2024\LKF_ Yang 19574 (010824) YSF590 NEG.d	Acquisition Date	8/1/2024 11:00:32 AM
Method	230724VGalanstune_low.m	Operator	demo
Sample Name	Yang 19574 (010824) YSF590 NEG	Instrument	micrOTOF 8213750.10408
Comment	Yang 19574 (010824) YSF590 NEG		

Acquisition Parameter

Source Type	ESI	Ion Polarity	Negative	Set Nebulizer	0.4 Bar
Focus	Not active			Set Dry Heater	180 °C
Scan Begin	50 m/z	Set Capillary	3000 V	Set Dry Gas	6.0 l/min
Scan End	3000 m/z	Set End Plate Offset	-500 V	Set Divert Valve	Waste

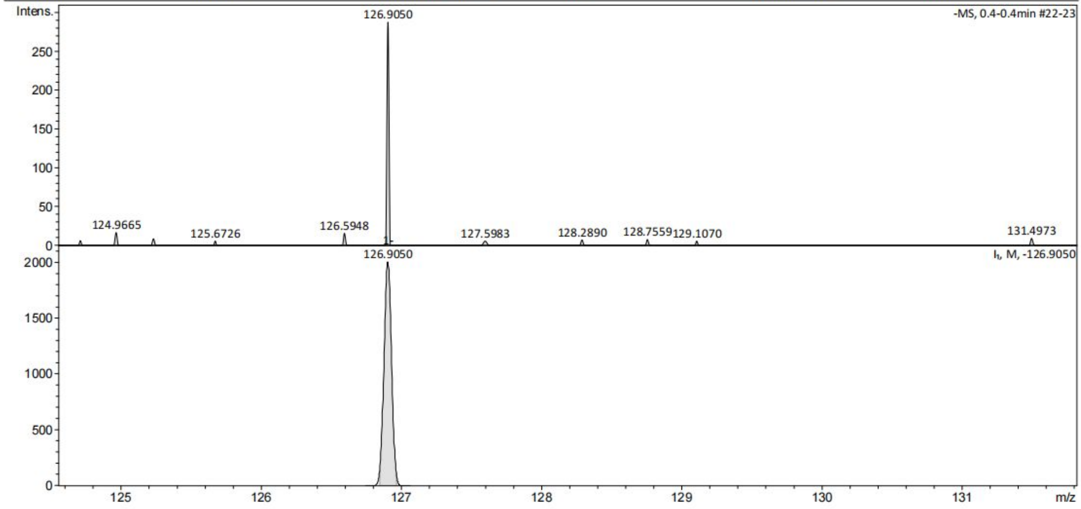

Bruker Compass DataAnalysis 4.1 printed: 8/1/2024 11:03:48 AM by: demo 1 of 1

Mass spec of SY 2

Display Report

Analysis Info

Analysis Name	D:\Data\Alans Data May 2024\LKF_Yang 18227 (010524) YSF59A.d	Acquisition Date	5/1/2024 9:58:24 AM
Method	AAlansVGD2tune090223_low.m	Operator	demo
Sample Name	Yang 18227 (010524) YSF59A	Instrument	micrOTOF 8213750.10408
Comment	Yang 18227 (010524) YSF59A		

Acquisition Parameter

Source Type	ESI	Ion Polarity	Positive	Set Nebulizer	0.6 Bar
Focus	Not active			Set Dry Heater	200 °C
Scan Begin	50 m/z	Set Capillary	3500 V	Set Dry Gas	8.0 l/min
Scan End	3000 m/z	Set End Plate Offset	-500 V	Set Divert Valve	Waste

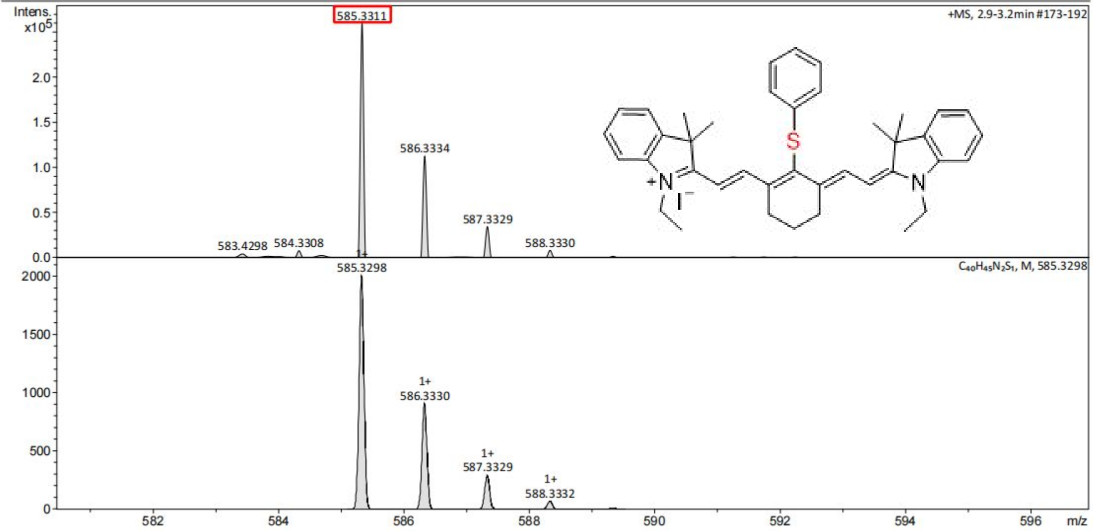

Bruker Compass DataAnalysis 4.1 printed: 5/1/2024 10:17:38 AM by: demo 1 of 1

Display Report

Analysis Info

Analysis Name	D:\Data\Alans Data May 2024\LKF_Yang 18227 (010524) YSF59A neg.d	Acquisition Date	5/1/2024 10:03:23 AM
Method	AAlansVGD2tune090223_low.m	Operator	demo
Sample Name	Yang 18227 (010524) YSF59A neg	Instrument	micrOTOF 8213750.10408
Comment	Yang 18227 (010524) YSF59A neg		

Acquisition Parameter

Source Type	ESI	Ion Polarity	Negative	Set Nebulizer	0.6 Bar
Focus	Not active			Set Dry Heater	200 °C
Scan Begin	50 m/z	Set Capillary	3500 V	Set Dry Gas	8.0 l/min
Scan End	3000 m/z	Set End Plate Offset	-500 V	Set Divert Valve	Waste

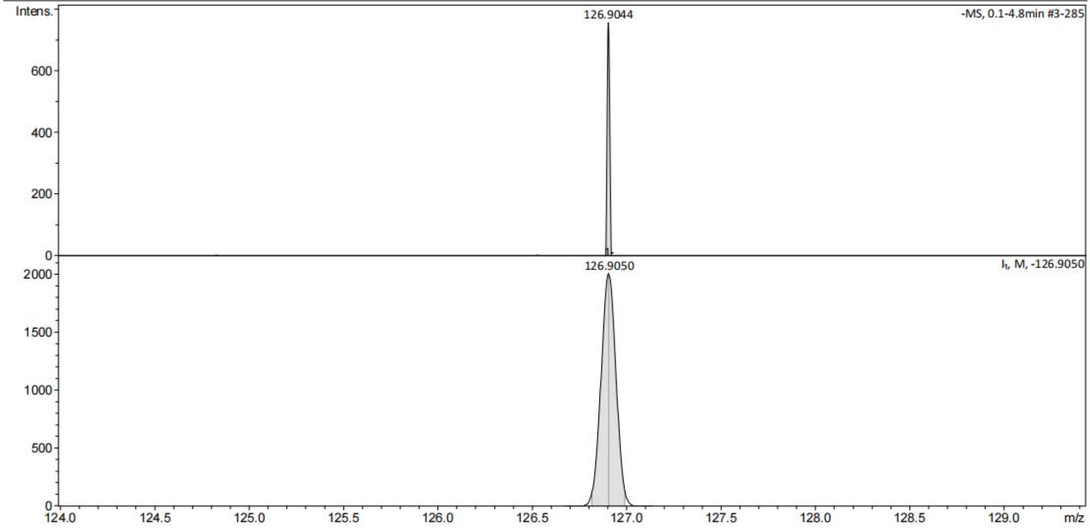

Bruker Compass DataAnalysis 4.1 printed: 5/1/2024 10:15:09 AM by: demo 1 of 1

Mass spec of SY 3

Display Report

Analysis Info

Analysis Name	D:\Data\Alans Data May 2024\LKF_Yang 18228 (010524) YSF59SE.d	Acquisition Date	5/1/2024 10:24:17 AM
Method	AAlansVGD2tune090223_low.m	Operator	demo
Sample Name	Yang 18228 (010524) YSF59SE	Instrument	micrOTOF 8213750.10408
Comment	Yang 18228 (010524) YSF59SE		

Acquisition Parameter

Source Type	ESI	Ion Polarity	Positive	Set Nebulizer	0.6 Bar
Focus	Not active			Set Dry Heater	200 °C
Scan Begin	50 m/z	Set Capillary	3500 V	Set Dry Gas	6.0 l/min
Scan End	3000 m/z	Set End Plate Offset	-500 V	Set Divert Valve	Waste

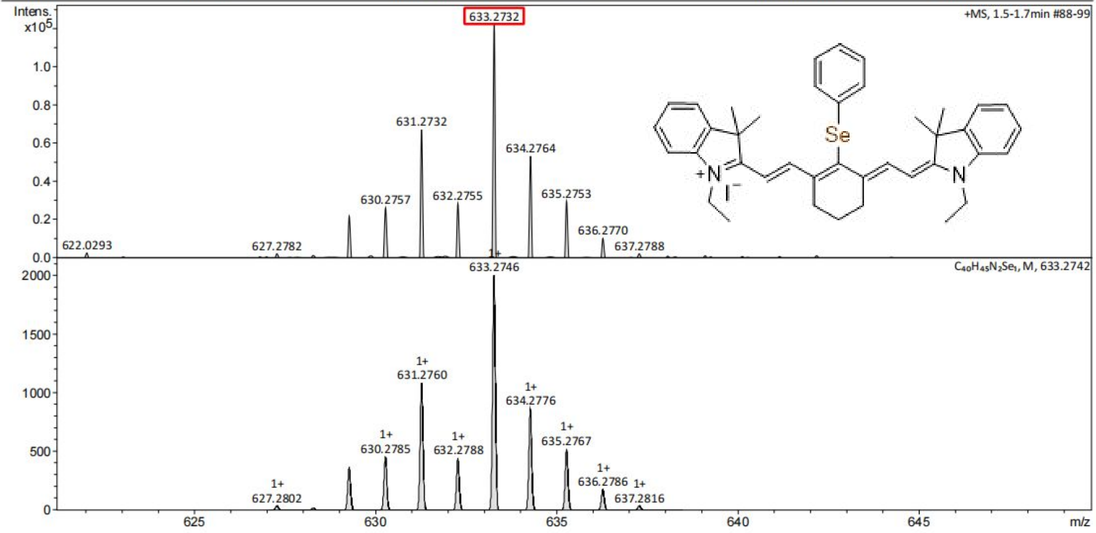

Bruker Compass DataAnalysis 4.1 printed: 5/1/2024 10:39:27 AM by: demo 1 of 1

Display Report

Analysis Info

Analysis Name	D:\Data\Alans Data May 2024\LKF_Yang 18228 (010524) YSF59SE NEG.d	Acquisition Date	5/1/2024 10:28:04 AM
Method	AAlansVGD2tune090223_low.m	Operator	demo
Sample Name	Yang 18228 (010524) YSF59SE NEG	Instrument	micrOTOF 8213750.10408
Comment	Yang 18228 (010524) YSF59SE NEG		

Acquisition Parameter

Source Type	ESI	Ion Polarity	Negative	Set Nebulizer	0.6 Bar
Focus	Not active			Set Dry Heater	200 °C
Scan Begin	50 m/z	Set Capillary	3500 V	Set Dry Gas	6.0 l/min
Scan End	3000 m/z	Set End Plate Offset	-500 V	Set Divert Valve	Waste

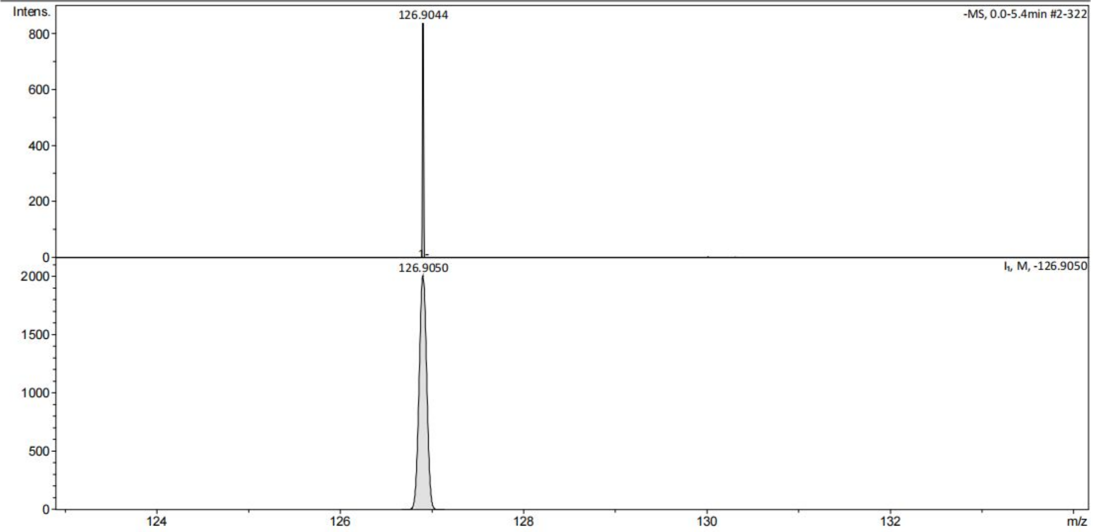

Bruker Compass DataAnalysis 4.1 printed: 5/1/2024 10:36:46 AM by: demo 1 of 1

Mass spec of SY 4

Display Report

Analysis Info

Analysis Name	D:\Data\Alans Data July 2024\LKF_Yang 19575 (310724) YSF550.d	Acquisition Date	7/31/2024 3:03:05 PM
Method	230724VGalanstune_low.m	Operator	demo
Sample Name	Yang 19575 (310724) YSF550	Instrument	micrOTOF 8213750.10408
Comment	Yang 19575 (310724) YSF550		

Acquisition Parameter

Source Type	ESI	Ion Polarity	Positive	Set Nebulizer	0.2 Bar
Focus	Not active			Set Dry Heater	200 °C
Scan Begin	50 m/z	Set Capillary	3500 V	Set Dry Gas	6.0 l/min
Scan End	3000 m/z	Set End Plate Offset	-500 V	Set Divert Valve	Waste

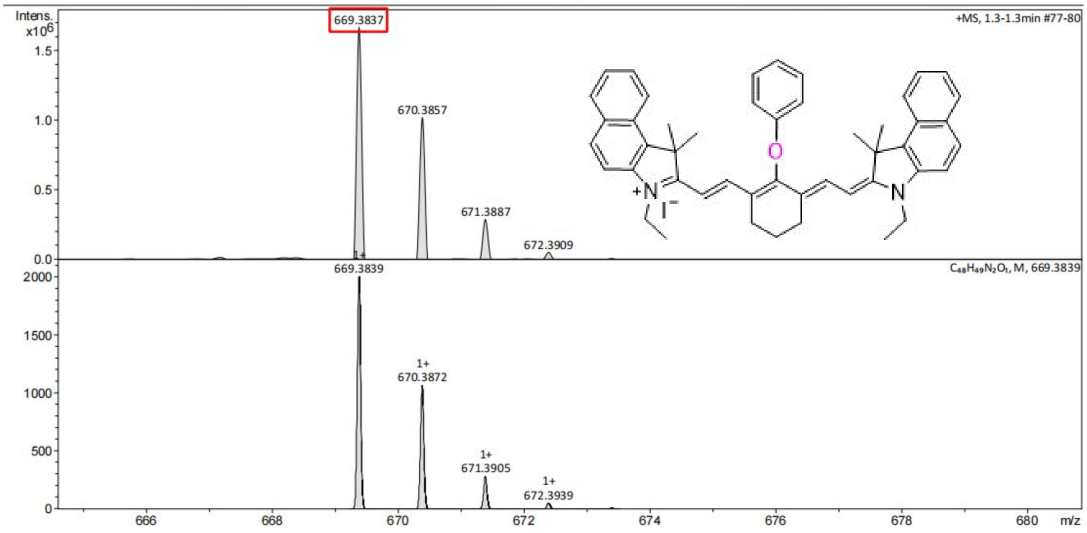

Bruker Compass DataAnalysis 4.1 printed: 7/31/2024 3:08:20 PM by: demo 1 of 1

Display Report

Analysis Info

Analysis Name	D:\Data\Alans Data Aug 2024\LKF_ Yang 19575 (010824) YSF550 NEG.d	Acquisition Date	8/1/2024 11:07:08 AM
Method	230724VGalanstune_low.m	Operator	demo
Sample Name	Yang 19575 (010824) YSF550 NEG	Instrument	micrOTOF 8213750.10408
Comment	Yang 19575 (010824) YSF550 NEG		

Acquisition Parameter

Source Type	ESI	Ion Polarity	Negative	Set Nebulizer	0.4 Bar
Focus	Not active			Set Dry Heater	180 °C
Scan Begin	50 m/z	Set Capillary	3000 V	Set Dry Gas	6.0 l/min
Scan End	3000 m/z	Set End Plate Offset	-500 V	Set Divert Valve	Waste

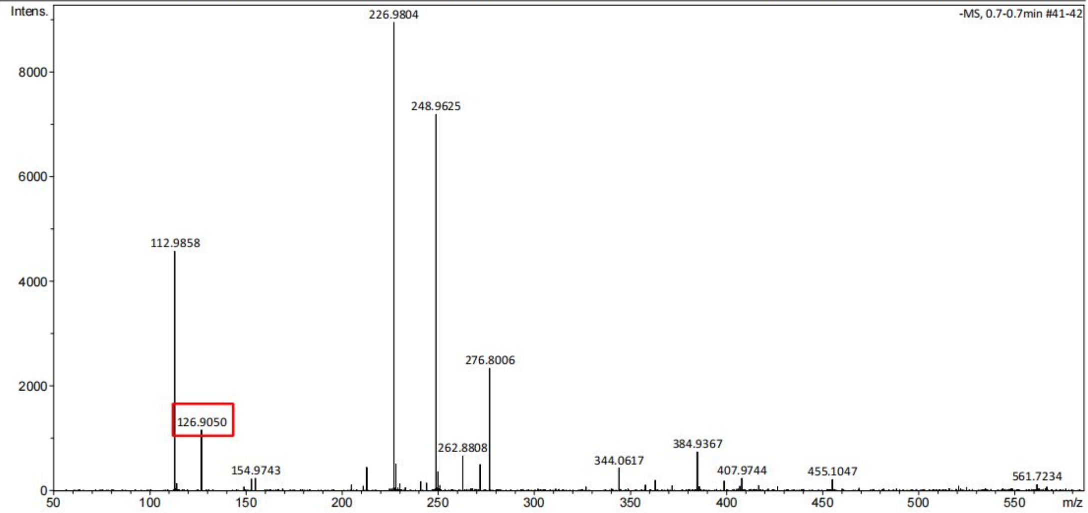

Bruker Compass DataAnalysis 4.1 printed: 8/1/2024 11:09:52 AM by: demo 1 of 1

Mass spec of SY 5

Display Report

Analysis Info

Analysis Name	D:\Data\Alans Data May 2024\LKF_Yang 18229 (010524) YSF55A.d	Acquisition Date	5/1/2024 10:43:51 AM
Method	AAlansVGD2tune090223_low.m	Operator	demo
Sample Name	Yang 18229 (010524) YSF55A	Instrument	micrOTOF 8213750.10408
Comment	Yang 18229 (010524) YSF55A		

Acquisition Parameter

Source Type	ESI	Ion Polarity	Positive	Set Nebulizer	0.6 Bar
Focus	Not active			Set Dry Heater	200 °C
Scan Begin	50 m/z	Set Capillary	3500 V	Set Dry Gas	8.0 l/min
Scan End	3000 m/z	Set End Plate Offset	-500 V	Set Divert Valve	Waste

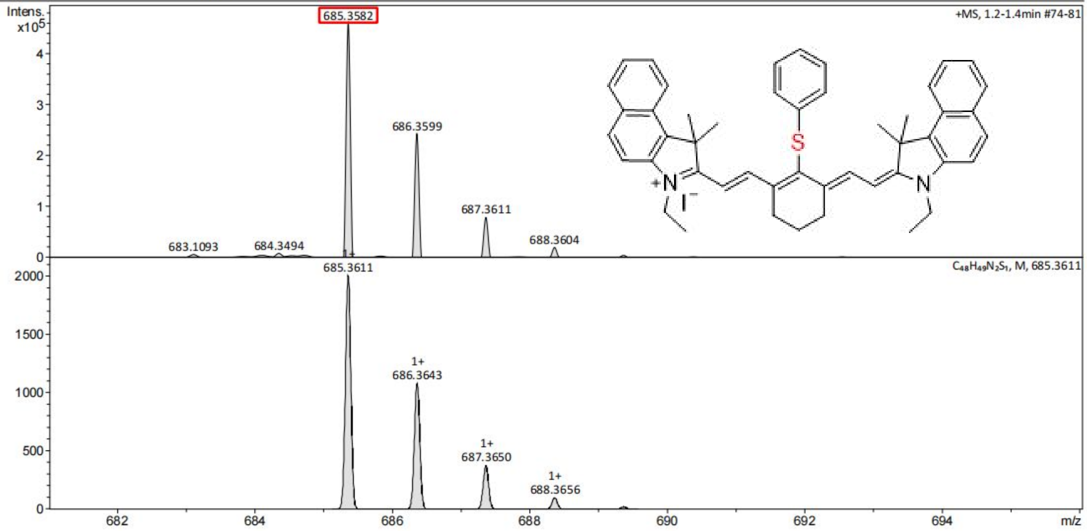

Bruker Compass DataAnalysis 4.1 printed: 5/1/2024 11:00:29 AM by: demo 1 of 1

Display Report

Analysis Info

Analysis Name	D:\Data\Alans Data May 2024\LKF_Yang 18229 (010524) YSF55A NEG.d	Acquisition Date	5/1/2024 10:48:25 AM
Method	AAlansVGD2tune090223_low.m	Operator	demo
Sample Name	Yang 18229 (010524) YSF55A NEG	Instrument	micrOTOF 8213750.10408
Comment	Yang 18229 (010524) YSF55A NEG		

Acquisition Parameter

Source Type	ESI	Ion Polarity	Negative	Set Nebulizer	0.6 Bar
Focus	Not active			Set Dry Heater	200 °C
Scan Begin	50 m/z	Set Capillary	2500 V	Set Dry Gas	8.0 l/min
Scan End	3000 m/z	Set End Plate Offset	-500 V	Set Divert Valve	Waste

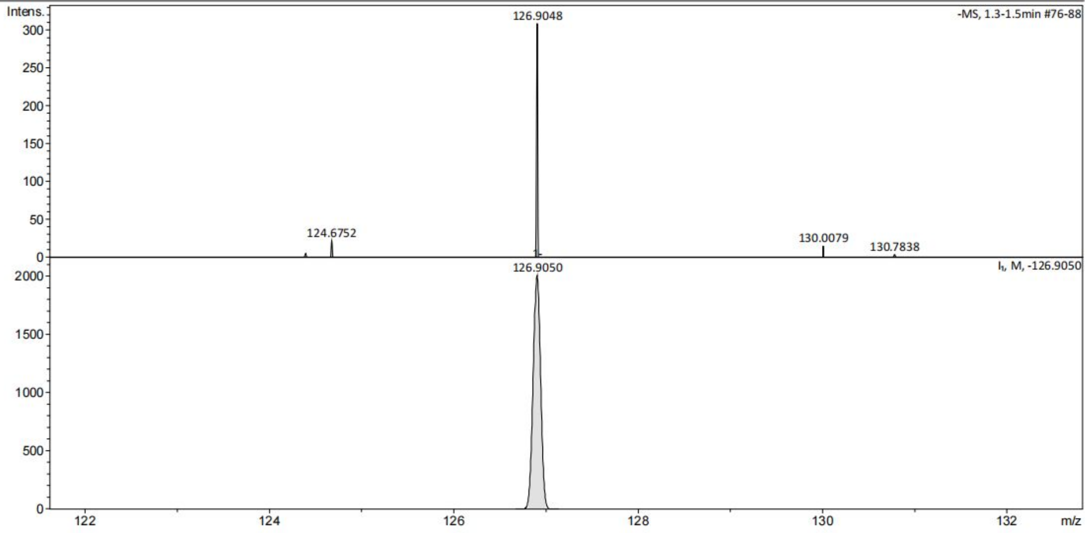

Bruker Compass DataAnalysis 4.1 printed: 5/1/2024 10:56:59 AM by: demo 1 of 1

Mass spec of SY 6

Display Report

Analysis Info

Analysis Name	D:\Data\Alans Data May 2024\LKF_Yang 18230 (010524) SF55SE.d	Acquisition Date	5/1/2024 12:04:55 PM
Method	AAlansVGD2tune090223_low.m	Operator	demo
Sample Name	Yang 18230 (010524) SF55SE	Instrument	micrOTOF 8213750.10408
Comment	Yang 18230 (010524) SF55SE		

Acquisition Parameter

Source Type	ESI	Ion Polarity	Positive	Set Nebulizer	0.6 Bar
Focus	Not active			Set Dry Heater	200 °C
Scan Begin	50 m/z	Set Capillary	3500 V	Set Dry Gas	6.0 l/min
Scan End	3000 m/z	Set End Plate Offset	-500 V	Set Divert Valve	Waste

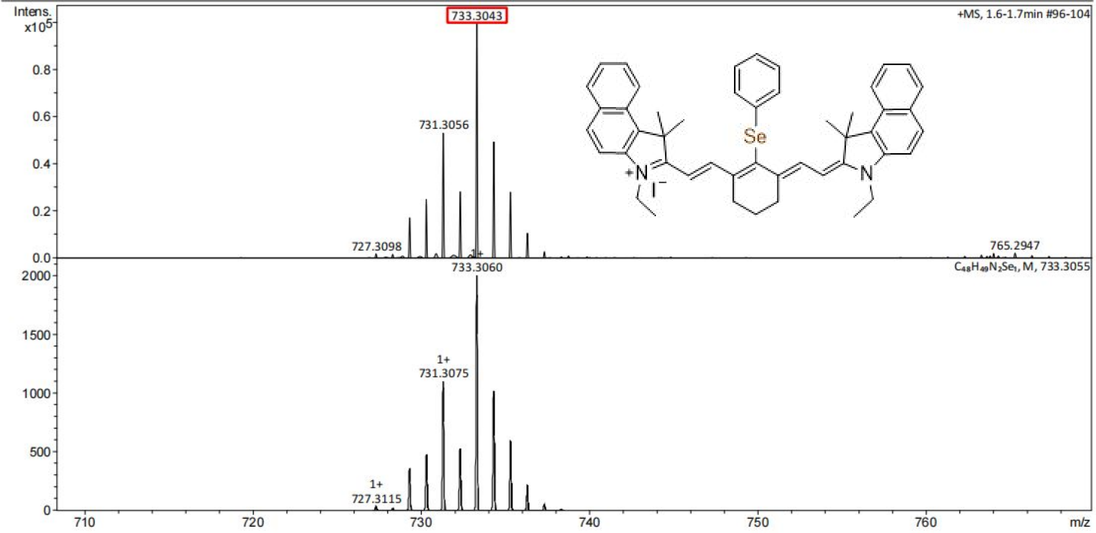

Bruker Compass DataAnalysis 4.1 printed: 5/1/2024 12:28:35 PM by: demo 1 of 1

Display Report

Analysis Info

Analysis Name	D:\Data\Alans Data May 2024\LKF_Yang 18230 (010524) SF55SE NEG 2.d	Acquisition Date	5/1/2024 12:22:49 PM
Method	AAlansVGD2tune090223_low.m	Operator	demo
Sample Name	Yang 18230 (010524) SF55SE NEG 2	Instrument	micrOTOF 8213750.10408
Comment	Yang 18230 (010524) SF55SE NEG 2		

Acquisition Parameter

Source Type	ESI	Ion Polarity	Negative	Set Nebulizer	0.6 Bar
Focus	Not active			Set Dry Heater	200 °C
Scan Begin	50 m/z	Set Capillary	2500 V	Set Dry Gas	8.0 l/min
Scan End	3000 m/z	Set End Plate Offset	-500 V	Set Divert Valve	Waste

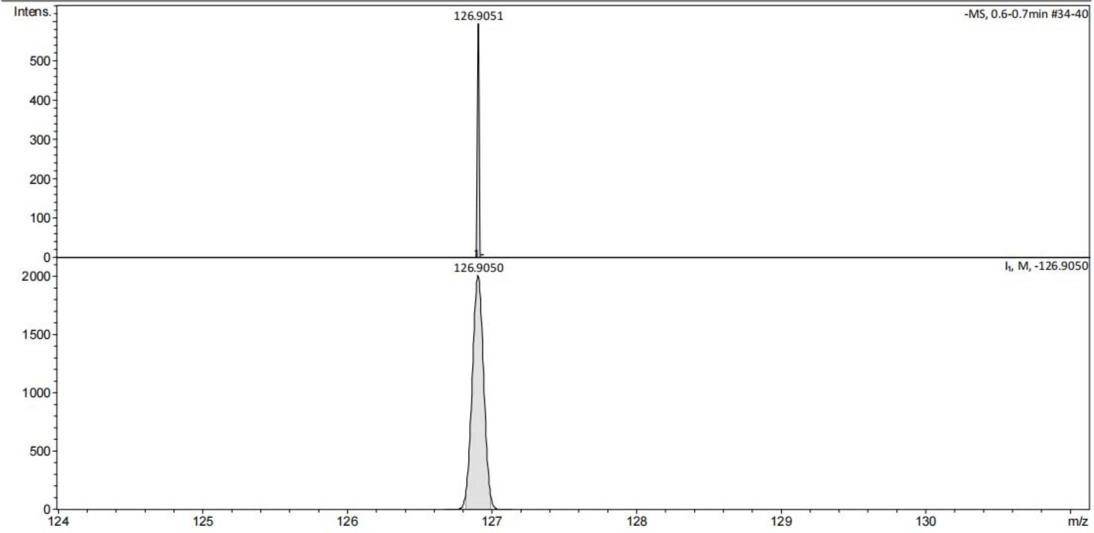

Bruker Compass DataAnalysis 4.1 printed: 5/1/2024 12:26:05 PM by: demo 1 of 1

Mass spec of SY 7

Display Report

Analysis Info

Analysis Name	D:\Data\Alans data June 2023\MBR_Yang 15787 (150623) YSF059.d	Acquisition Date	6/15/2023 10:10:28 AM
Method	AAlansVGD2tune090223_low.m	Operator	demo
Sample Name	Yang 15787 (150623) YSF059	Instrument	micrOTOF 8213750.10408
Comment	Yang 15787 (150623) YSF059		

Acquisition Parameter

Source Type	ESI	Ion Polarity	Positive	Set Nebulizer	0.4 Bar
Focus	Not active			Set Dry Heater	200 °C
Scan Begin	50 m/z	Set Capillary	3500 V	Set Dry Gas	6.0 l/min
Scan End	3000 m/z	Set End Plate Offset	-500 V	Set Divert Valve	Waste

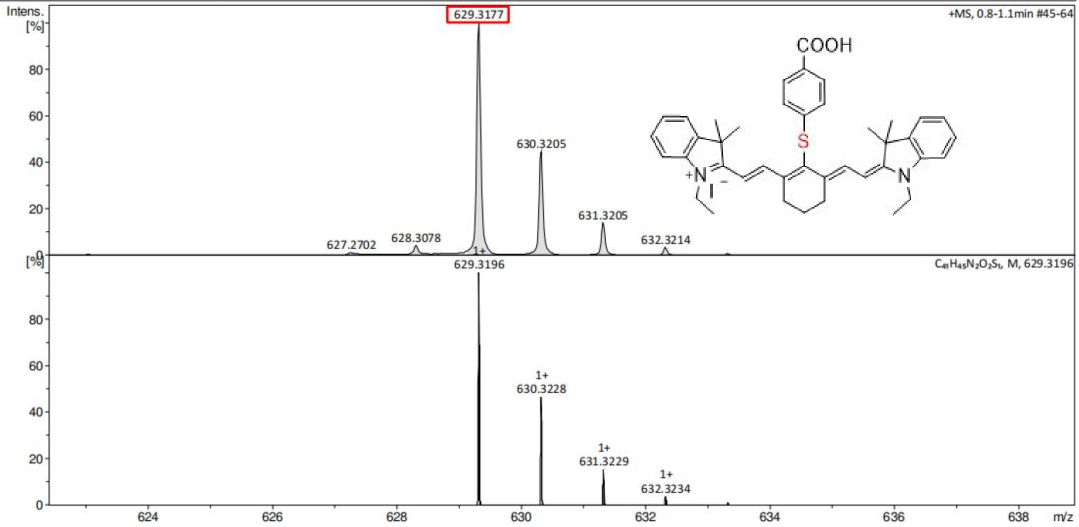

Display Report

Analysis Info

Analysis Name	D:\Data\Alans data June 2023\MBR_Yang 15787 (150623) YSF059 NEG.d	Acquisition Date	6/15/2023 10:32:58 AM
Method	AAlansVGD2tune090223_low.m	Operator	demo
Sample Name	Yang 15787 (150623) YSF059 NEG	Instrument	micrOTOF 8213750.10408
Comment	Yang 15787 (150623) YSF059 NEG		

Acquisition Parameter

Source Type	ESI	Ion Polarity	Negative	Set Nebulizer	0.4 Bar
Focus	Not active			Set Dry Heater	200 °C
Scan Begin	50 m/z	Set Capillary	3000 V	Set Dry Gas	6.0 l/min
Scan End	3000 m/z	Set End Plate Offset	-500 V	Set Divert Valve	Waste

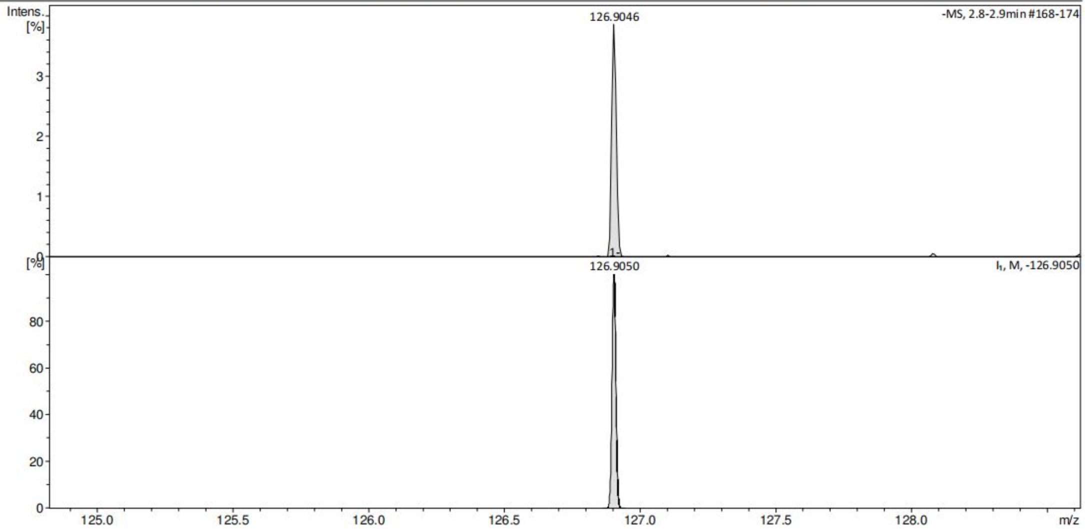

Mass spec of SY 8

Display Report

Analysis Info

Analysis Name	D:\Data\Alans data March 2023\MBR_Yang 15257 (240323) YSF055.d	Acquisition Date	3/24/2023 12:44:12 PM
Method	AAlansVGD2tune090223_low.m	Operator	Bruker UK
Sample Name	Yang 15257 (240323) YSF055	Instrument	micrOTOF 8213750.10408
Comment	Yang 15257 (240323) YSF055		

Acquisition Parameter

Source Type	ESI	Ion Polarity	Positive	Set Nebulizer	0.4 Bar
Focus	Not active			Set Dry Heater	230 °C
Scan Begin	50 m/z	Set Capillary	3500 V	Set Dry Gas	5.0 l/min
Scan End	3000 m/z	Set End Plate Offset	-500 V	Set Divert Valve	Waste

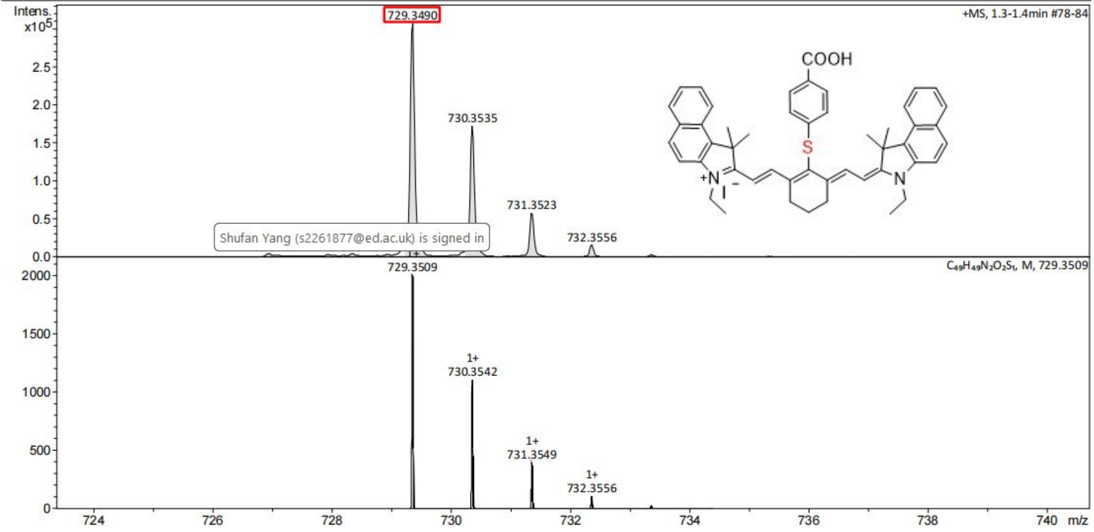

Bruker Compass DataAnalysis 4.1 printed: 3/24/2023 12:49:48 PM by: Bruker UK 1 of 1

Display Report

Analysis Info

Analysis Name	D:\Data\Alans data March 2023\MBR_Yang 15257 (240323) YSF055 NEG.d	Acquisition Date	3/24/2023 12:53:42 PM
Method	AAlansVGD2tune090223_low.m	Operator	Bruker UK
Sample Name	Yang 15257 (240323) YSF055 NEG	Instrument	micrOTOF 8213750.10408
Comment	Yang 15257 (240323) YSF055 NEG		

Acquisition Parameter

Source Type	ESI	Ion Polarity	Negative	Set Nebulizer	1.0 Bar
Focus	Not active			Set Dry Heater	230 °C
Scan Begin	50 m/z	Set Capillary	3000 V	Set Dry Gas	6.0 l/min
Scan End	3000 m/z	Set End Plate Offset	-500 V	Set Divert Valve	Waste

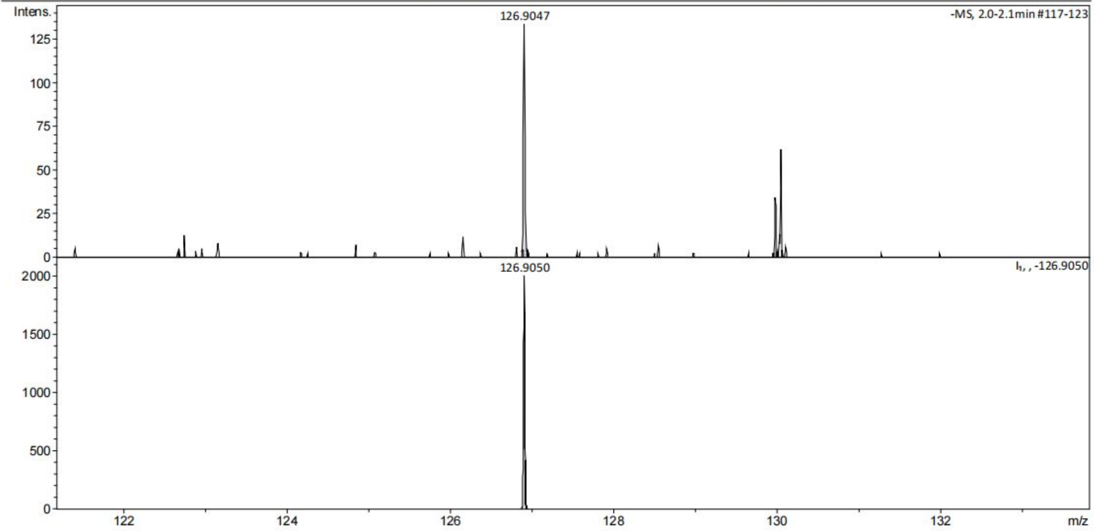

Bruker Compass DataAnalysis 4.1 printed: 3/24/2023 1:01:25 PM by: Bruker UK 1 of 1

7. Non-normalised UV–vis spectra of the SY 1 – SY 8

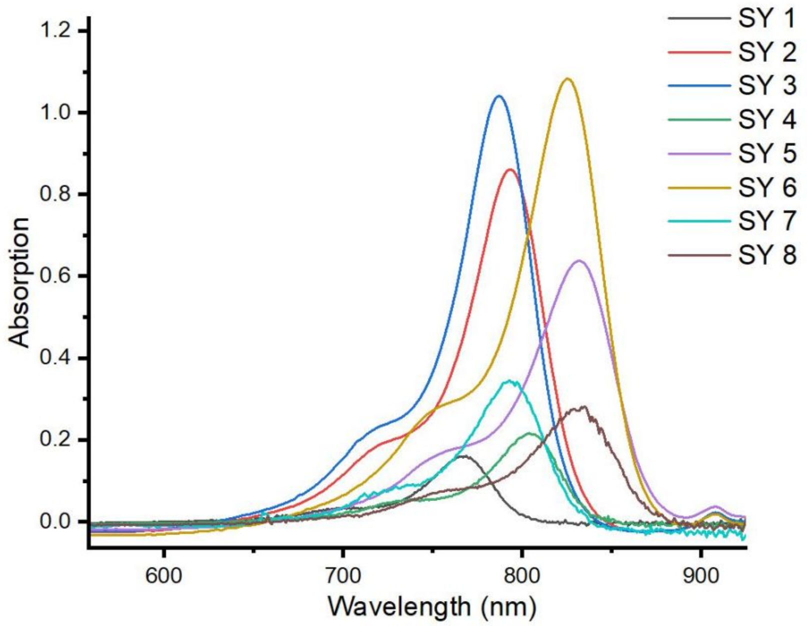

8. References

1. Frisch, M. J.; Trucks, G. W.; Schlegel, H. B.; Scuseria, G. E.; Robb, M. A.; Cheeseman, J. R.; Scalmani, G.; Barone, V.; Petersson, G. A.; Nakatsuji, H.; Li, X.; Caricato, M.; Marenich, A. V.; Bloino, J.; Janesko, B. G.; Gomperts, R.; Mennucci, B.; Hratchian, H. P.; Ortiz, J. V.; Izmaylov, A. F.; Sonnenberg, J. L.; Williams-Young, D.; Ding, F.; Lipparini, F.; Egidi, F.; Goings, J.; Peng, B.; Petrone, A.; Henderson, T.; Ranasinghe, D.; Zakrzewski, V. G.; Gao, J.; Rega, N.; Zheng, G.; Liang, W.; Hada, M.; Ehara, M.; Toyota, K.; Fukuda, R.; Hasegawa, J.; Ishida, M.; Nakajima, T.; Honda, Y.; Kitao, O.; Nakai, H.; Vreven, T.; Throssell, K.; Montgomery, J. A., Jr.; Peralta, J. E.; Ogliaro, F.; Bearpark, M. J.; Heyd, J. J.; Brothers, E. N.; Kudin, K. N.; Staroverov, V. N.; Keith, T. A.; Kobayashi, R.; Normand, J.; Raghavachari, K.; Rendell, A. P.; Burant, J. C.; Iyengar, S. S.; Tomasi, J.; Cossi, M.; Millam, J. M.; Klene, M.; Adamo, C.; Cammi, R.; Ochterski, J. W.; Martin, R. L.; Morokuma, K.; Farkas, O.; Foresman, J. B.; Fox, D. J. **Gaussian 16, Revision B.01**; Gaussian, Inc.: Wallingford CT, 2016.
2. Becke, A. D. Density-functional thermochemistry. III. The role of exact exchange. *J. Chem. Phys.* **1993,** *98,* 5648–5652.
3. Lee, C.; Yang, W.; Parr, R. G. Development of the Colle–Salvetti correlation-energy formula into a functional of the electron density. *Phys. Rev. B* **1988,** *37,* 785–789.
4. Stephens, P. J.; Devlin, F. J.; Chabalowski, C. F.; Frisch, M. J. Ab initio calculation of vibrational absorption and circular dichroism spectra using density functional force fields. *J. Phys. Chem.* **1994,** *98,* 11623–11627.
5. McLean, A. D.; Chandler, G. S. Contracted Gaussian basis sets for molecular calculations. I. Second row atoms, Z = 11–18. *J. Chem. Phys.* **1980,** *72,* 5639–5648.
6. Krishnan, R.; Binkley, J. S.; Seeger, R.; Pople, J. A. Self-consistent molecular orbital methods. XX. A basis set for correlated wave functions. *J. Chem. Phys.* **1980,** *72,* 650–654.
7. Tomasi, J.; Mennucci, B.; Cammi, R. Quantum mechanical continuum solvation models. *Chem. Rev.* **2005,** *105,* 2999–3093.
8. Stukowski, A. Visualization and analysis of atomistic simulation data with OVITO—The Open Visualization Tool. *Model. Simul. Mater. Sci. Eng.* **2010,** *18,* 015012.
9. Collot, M.; Kreder, R.; Tatarets, A. L.; Patsenker, L. D.; Mély, Y.; Klymchenko, A. S. Bright fluorogenic squaraines with tuned cell entry for selective imaging of plasma membrane vs endoplasmic reticulum. *Chem. Commun.* **2015,** *51,* 17136–17139.
10. Véron, A. C.; Zhang, H.; Linden, A.; Nüesch, F.; Heier, J.; Hany, R.; Geiger, T. NIR-absorbing heptamethine dyes with tailor-made counterions for application in light to energy conversion. *Org. Lett.* **2014,** *16,* 1044–1047.
11. Mokbel, H.; Noirbent, G.; Gigmes, D.; Dumur, F.; Lalevée, J. Towards new NIR dyes for free radical photopolymerization processes. *Beilstein J. Org. Chem.* **2021,** *17,* 2067–2076.
12. Ghann, W.; Kang, H.; Emerson, E.; Oh, J.; Chavez-Gil, T.; Nesbitt, F.; Williams, R.; Uddin, J. Photophysical properties of near-IR cyanine dyes and their application as photosensitizers in dye-sensitized solar cells. *Inorg. Chim. Acta* **2017,** *467,* 123–131.
